# Silver-catalyzed synthesis of β-fluorovinylphosphonates by phosphonofluorination of aromatic alkynes

**DOI:** 10.3762/bjoc.16.258

**Published:** 2020-12-18

**Authors:** Yajing Zhang, Qingshan Tian, Guozhu Zhang, Dayong Zhang

**Affiliations:** 1 School of Science, China Pharmaceutical University, 24 Tongjiaxiang, Nanjing 210000, P. R. China; 2 State Key Laboratory of Organometallic Chemistry, Shanghai Institute of Organic Chemistry, Chinese Academy of Sciences, 345 Lingling Road, Shanghai 200032, P. R. China

**Keywords:** β-fluorovinylphosphonates, fluorine atom transfer, P-centered radical, silver catalysis, three-component reaction

## Abstract

A silver-catalyzed three-component reaction involving alkynes, Selectfluor^®^, and diethyl phosphite was employed for the one-pot formation of C(sp^2^)–F and C(sp^2^)–P bonds to provide an efficient access to β-fluorovinylphosphonates in a highly regio- and stereoselective manner under mild reaction conditions. This reaction is operationally simple and offers an excellent functional group tolerance as well as a broad substrate scope that includes both terminal and internal alkynes. The reaction proceeded through the oxidative generation of a P-centered radical and subsequent fluorine atom transfer.

## Introduction

As one of the most important topics in organic chemistry, the introduction of fluorine and phosphorus atoms into double bonds is an attractive approach for the synthesis of a variety of valuable organic compounds [1–7]. Although progress has been achieved in the formation of C(sp^2^)–F bonds from various substrates [8–13], new catalytic reactions to introduce fluorine and phosphorus are seldom reported.

**Scheme 1:**
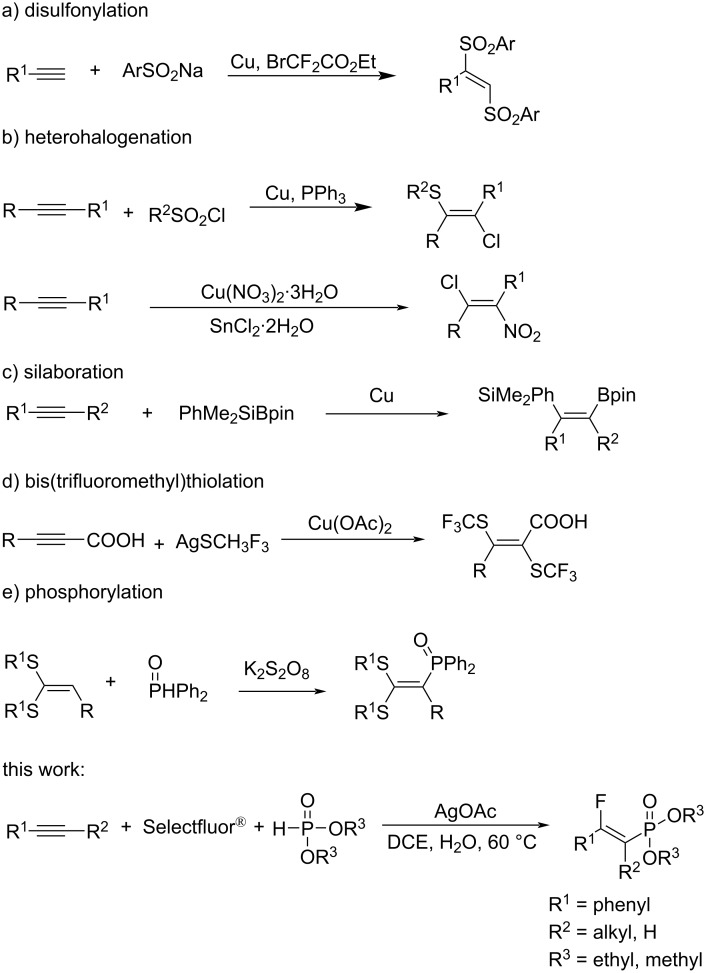
Metal-catalyzed difunctionalization of unsaturated carbon–carbon bonds.

Among the strategies for constructing diverse alkenes containing two-heteroatom bonds, such as disulfonylation [14–16], heterohalogenation [17–20], bis(trifluoromethyl)thiolation [21], and phosphorylation [22], the direct heterodifunctionalization of alkynes using three-component reactions is the most rapid and convenient one (Scheme 1). Although studies on alkyne difunctionalization are ongoing [23], the successful attachment of a fluorine atom to the resulting alkene through transition metal catalysis remains a challenge. In particular, the phosphonofluorination of alkynes for the introduction of two strong electron-withdrawing groups into double bonds has not yet been reported. With our continuous interest in, and inspiration from the well-established transition metal-catalyzed radical difunctionalization of unsaturated carbon–carbon bonds, specifically the work of Li’s group and others concerning the silver-catalyzed phosphonofluorination of alkenes [24–26], we herein present a general silver-catalyzed regio- and stereoselective phosphonofluorination of alkynes using Selectfluor^®^ and phosphonates as reactants (Scheme 1). This new silver-catalyzed approach to fluorinated vinylphosphonates from simple and commercially available alkynes proceeds under mild conditions with high stereoselectivity, and thus enabling the rapid construction of molecular complexity.

## Results and Discussion

We investigated the reaction of phenylacetylene (**1a**), Selectfluor^®^, and diethyl phosphite to afford β-fluorovinylphosphonates under mild conditions. First, we examined the catalytic activity of various transition metal catalysts, including Au, Ag, Cu, Rh, and Pd complexes (Supporting Information File 1). Among these metal salts, silver salts, especially AgOAc, were the most effective catalysts for generating the desired product **2a** (Table 1, entries 1–8). The influence of different reaction temperatures on the reaction outcome was also investigated. At room temperature, the product **2a** was obtained in 72% yield (Table 1, entry 8). Increasing the reaction temperature to 60 °C further improved the yield to 79% (Table 1, entry 9), although only a low yield was obtained when the reaction was performed at 100 °C (Table 1, entry 10). The reaction outcome was greatly affected by the solvent used. We carried out this reaction in DCE as the only solvent, and no product was obtained (Table 1, entry 15). The use of EtOAc and H_2_O or of THF and H_2_O, instead of DCE and H_2_O, afforded the monofluoroalkene **2a** in only a low yield (Table 1, entries 11 and 12), whereas the product **2a** was not obtained when using acetonitrile and H_2_O or when using DMF (Table 1, entries 13 and 14). Similarly, the desired product was not obtained in the absence of a silver catalyst (Table 1, entry 16). The extensive screening of these reaction parameters revealed that the treatment of the alkynes **1** with Selectfluor^®^, diethyl phosphite, and silver acetate as the catalyst as well as DCE/H_2_O 1:1 as the solvent at 60 °C afforded the optimal result.

**Table 1:** Optimization of the reaction conditions.

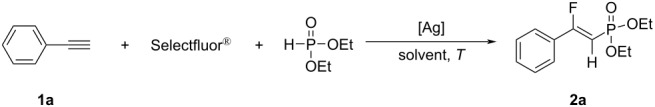				

entry^a^	catalyst	solvent	*T* (°C)	yield (%)^b^

1	Ag_2_CO_3_	DCE, H_2_O	rt	38
2	AgF	DCE, H_2_O	rt	24
3	AgOTf	DCE, H_2_O	rt	34
4	AgSbF_6_	DCE, H_2_O	rt	38
5	AgNO_3_	DCE, H_2_O	rt	58
6	AgNO_3_	DCE, H_2_O, AcOH	rt	62
7	AgNO_3_	DCE, H_2_O, TFA	rt	63
8	AgOAc	DCE, H_2_O	rt	72
9	AgOAc	DCE, H_2_O	60	79
10	AgOAc	DCE, H_2_O	100	20
11	AgOAc	EtOAc, H_2_O	60	33
12	AgOAc	THF, H_2_O	60	40
13	AgOAc	CH_3_CN, H_2_O	60	—
14	AgOAc	DMF	60	—
15	AgOAc	DCE	60	—
16	—	DCE, H_2_O	60	—

^a^Reaction conditions: **1a** (0.2 mmol), silver salt (0.02 mmol), diethyl phosphite (0.4 mmol), and Selectfluor^®^ (0.4 mmol) were stirred in the reaction solvent (1 mL:1 mL) at 60 °C for 24 h under N_2_. ^b^Isolated yield.

**Scheme 2:**
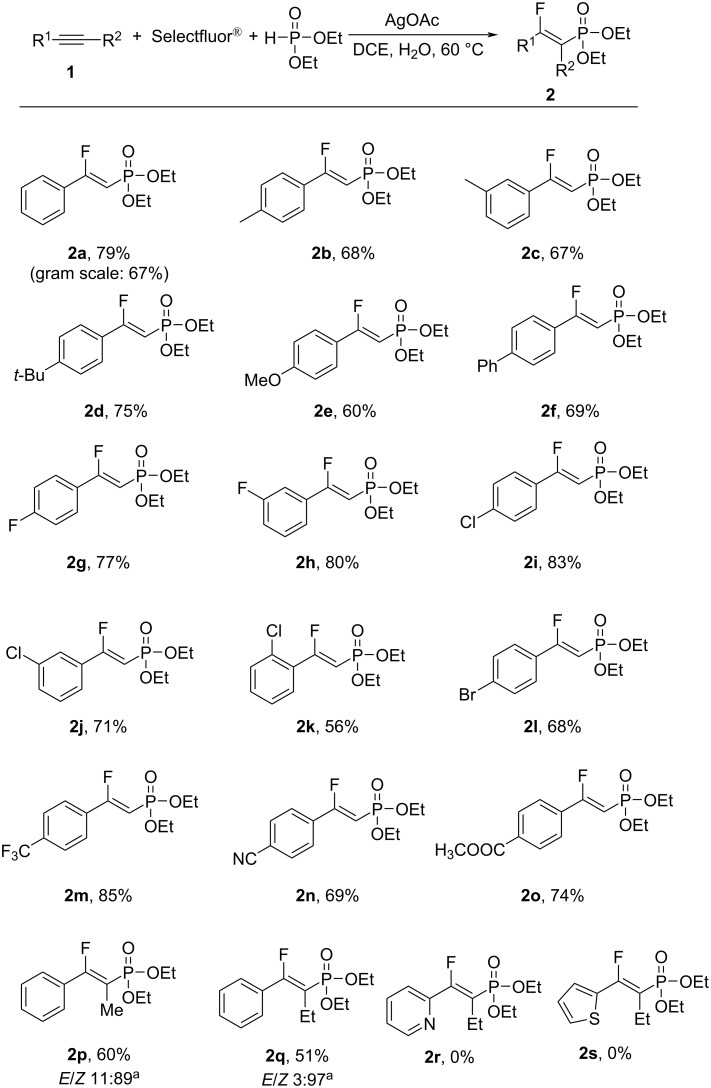
Substrate scope for the synthesis of the β-fluorovinylphosphonates **2** using diethyl phosphite. Reaction conditions: aromatic alkyne **1** (0.2 mmol), AgOAc (0.02 mmol), diethyl phosphite (0.4 mmol), and Selectfluor^®^ (0.4 mmol) were stirred in DCE/H_2_O 1 mL:1 mL at 60 °C for 24 h under N_2_. The yields of the isolated products are shown. ^a^The *E*/*Z* ratio was determined by ^1^H NMR analysis of the reaction mixture after the reaction reached completion.

**Scheme 3:**
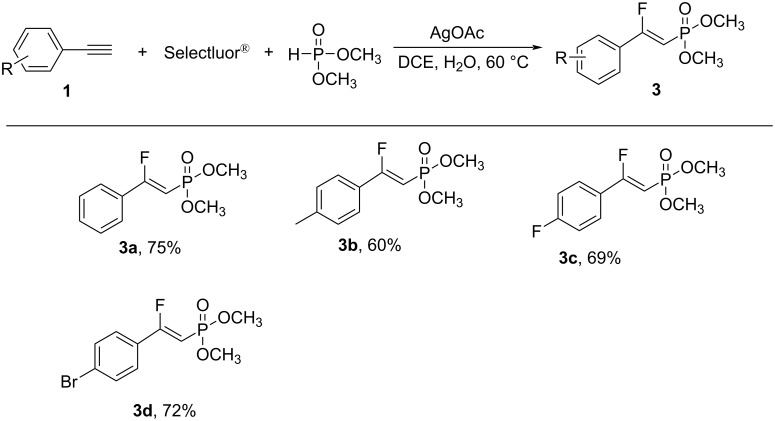
Substrate scope for the synthesis of the β-fluorovinylphosphonates **3** using dimethyl phosphite. Reaction conditions: aromatic alkyne **1** (0.2 mmol), AgOAc (0.02 mmol), dimethyl phosphite (0.4 mmol), and Selectfluor^®^ (0.4 mmol) were stirred in DCE/H_2_O 1 mL:1 mL at 60 °C for 24 h under N_2_. The yields of the isolated products are shown.

The substrate scope of the reaction was investigated under the optimized reaction conditions. As shown in Scheme 2, various β-fluorovinylphosphonates could be conveniently and efficiently obtained using the developed method. Aromatic alkynes bearing various functional groups, including both electron-rich and electron-deficient moieties at the *para*- and *meta*-positions generally reacted smoothly to afford the desired products **2a**–**o** in moderate to good yield. An aromatic alkyne possessing a Cl substituent at the *ortho*-position also reacted smoothly to provide the corresponding product **2k** in 56% yield. Substrates containing a range of functional groups, including halides (F, Cl, and Br), CF_3_, and even ester groups, were amenable to this transformation. The reaction was also compatible with a CN group to afford the corresponding product **2n** in 69% yield. Heteroaromatic alkynes, such as 2-ethynylpyridine and 2-ethynylthiophene, did not undergo the reaction to form **2r** and **2s**. Internal aromatic alkynes were tolerated and generated the desired products **2p** and **2q** in moderate yield. Aliphatic alkynes, such as (prop-2-yn-1-yl)benzene and (but-3-yn-1-yl)benzene, afforded the corresponding products in an extremely low yield. Another H-phosphonate, namely dimethyl phosphite, was a suitable substrate for this transformation and provided the products **3** in good yield (Scheme 3).

**Scheme 4:**
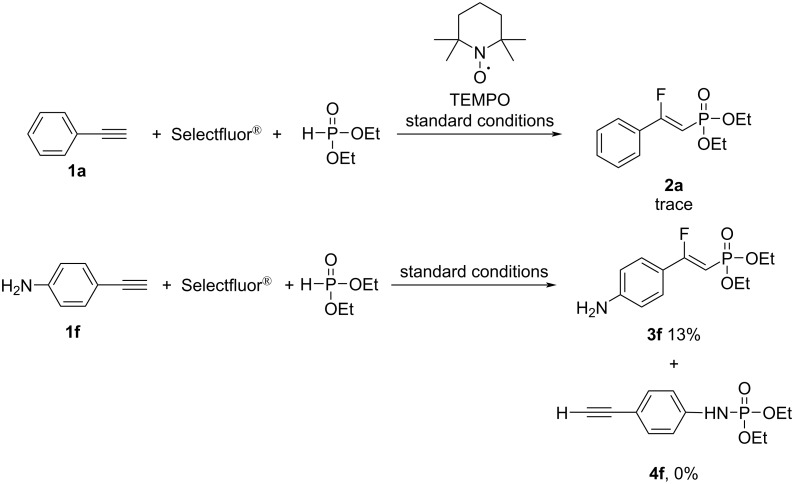
Radical-trapping experiments.

The well-known radical-trapping reagent 2,2,6,6-tetramethylpiperidine-*N*-oxyl (TEMPO) was used to gain an insight into the reaction mechanism. As illustrated in Scheme 4, the addition of 1 equiv TEMPO suppressed the reaction. In addition, 4-ethynylaniline (**1f**) reacted with Selectfluor^®^ and dimethyl phosphite under the optimized conditions to afford the expected product in 13% yield, and no competitive electrophilic compound **4f** was observed. These findings imply that the transformation may involve a radical process rather than an ionic process.

**Scheme 5:**
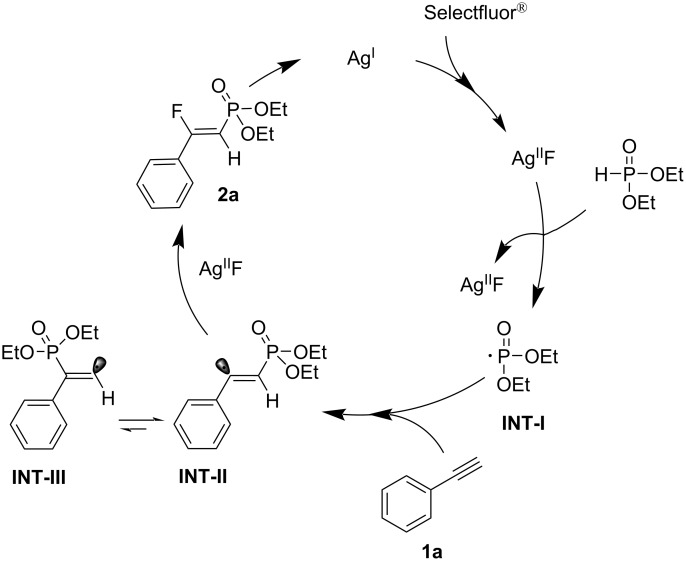
Proposed mechanism for the silver-catalyzed phosphonofluorination of alkynes.

On the basis of previous reports [23–26], we propose a radical mechanism involving the silver promoter, as illustrated in Scheme 5. In this mechanism, Ag(I) was oxidized by selectflour to generate Ag(III)F. Subsequently, ethyl phosphite is oxidized by Ag(III)F to generate a P-centered radical (**INT-I**) and Ag(II)F. The electrophilic phosphonyl radical addition to the triple bond of **1a** generates the vinyl-free radical **INT-II**, which is subsequently trapped by AgF(II) to afford the corresponding product **2a**. The vinyl radical **INT-II** stabilizes the unpaired electron through resonance, and **INT-II** is more stable than **INT-III** [27–28]. Finally, we obtained the *Z*-configured product with respect to the aryl and phosphonyl groups.

**Scheme 6:**
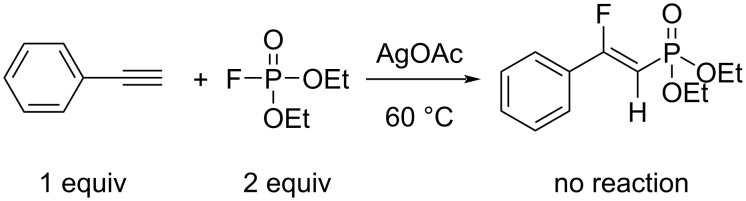
Attempted use of a suspected phosphonofluorination intermediate to synthesize a β-fluorovinylphosphonate.

We also performed control experiments under standard conditions to determine whether diethyl fluorophosphonate is a key intermediate in the reaction with aromatic alkynes. Based on a method by Gupta et al. [29], we obtained diethyl fluorophosphonate. Subsequently, the diethyl fluorophosphonate did not react with an aromatic alkyne in the desired phosphonofluorination so that no product was obtained (Scheme 6) [30].

## Conclusion

In conclusion, a novel method for the synthesis of β-fluorophosphonates through the direct phosphonofluorination of alkynes with Selectfluor^®^ and H-phosphonates under mild reaction conditions has been successfully developed. Owing to its broad substrate scope and excellent functional group tolerance, this simple protocol may represent a general, one-step approach for the preparation of β-fluorophosphonate frameworks for the use in medicinal chemistry.

## Supporting Information

File 1 Experimental procedures, full characterization of products, and NMR spectra.
