# Supplementary material for: Silver-catalyzed synthesis of β-fluorovinylphosphonates by phosphonofluorination of aromatic alkynes
Source: Beilstein J Org Chem. 2020 Dec 18;16:3086–92. doi: 10.3762/bjoc.16.258 (PMC7753110; doi:10.3762/bjoc.16.258)

BEILSTEIN JOURNAL OF ORGANIC CHEMISTRY

Supporting Information

for

Silver-catalyzed synthesis of β-fluorovinylphosphonates by phosphonofluorination of aromatic alkynes

Yajing Zhang, Qingshan Tian, Guozhu Zhang and Dayong Zhang

Beilstein J. Org. Chem. **2020,** *16,* 3086–3092. doi:10.3762/bjoc.16.258

Experimental procedures, full characterization of products, and NMR spectra

License and Terms: This is a supporting information file under the terms of the Creative Commons Attribution License (https://creativecommons.org/licenses/by/4.0). Please note that the reuse, redistribution and reproduction in particular requires that the author(s) and source are credited and that individual graphics may be subject to special legal provisions.
The license is subject to the *Beilstein Journal of Organic Chemistry* terms and conditions: (https://www.beilstein-journals.org/bjoc/terms)

Contents

1 Experimental details……..S1

1.1 Optimization of the reaction conditions...S1

2 General information S2

2.1 Gram-scale reaction S9

3 References...S9

4 Copies of NMR spectra...S10

1 Experimental Details

1.1 Optimization of the reaction conditions

Catalyst screening

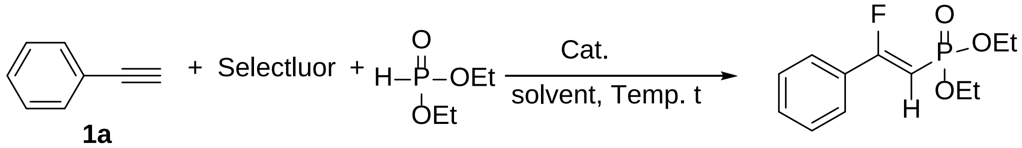

1a

2a

entry	catalyst	yield (%)[a]
1	CuI	trace
2	$AuCl_3$	9%
3	$(MeCN)_2PdCl_2$	no reaction
4	$[Rh(OH)(COD)]_2$	no reaction
5	$Cu(MeCN)_4PF_4$	3%
6	CuOAc	trace

[a]Yields determined by ^1H NMR analysis with an internal standard (diethyl phthalate).

2 General Information

All reagents were obtained from commercial sources and used without further purification. Thin-layer chromatography (TLC) was performed using 60 mesh silica gel plates visualized with short-wavelength UV light (254 nm). ^{1}H NMR spectra were obtained at 400 MHz and recorded relative to the tetramethylsilane signal (0 ppm) or residual proton solvent. ^{13}C NMR spectra were obtained at 100 MHz, and chemical shifts were recorded relative to the solvent resonance ($CDCl_3$, 77.16ppm). Data for ^{1}H NMR are reported as follows: chemical shift (δ, ppm) and multiplicity (s = singlet, d = doublet, t = triplet, q = quartet, m = multiplet or unresolved, br = broad singlet, coupling constant (s) in Hz, integration).

General procedure for the silver-catalyzed fluorophosphorylation of alkynes

In a vial, **1a** (20.4 mg, 0.2 mmol), Selectfluor® (141.7 mg, 0.4 mmol), diethyl phosphonate (55.2 mg, 0.4 mmol), and silver acetate (3.3 mg, 0.02 mmol) were dissolved in DEC/H_2O 1 mL:1 mL. The resulting mixture was stirred under N_2 atmosphere at 60 °C for 24 h. After completion, the reaction was quenched, and the suspension was extracted with CH_2Cl_2. Volatile solvent was evaporated in vacuo, and the residual oil was purified by column chromatography on silica gel with a gradient eluent of petroleum ether and ethyl acetate to afford the fluorine product **2a**. Note: A single *Z*-isomer of the product **2a** was established by comparison with previous reports [1,2].

Diethyl (*Z*)-(2-fluoro-2-phenylvinyl)phosphonate (2a)

The reaction was conducted on a 0.2 mmol scale following the general procedure. The desired product was purified by column chromatography (PE/EA 10:1) to give a colorless oil (40.9 mg, 79%).

^{1}H NMR (400 MHz, $CDCl_3$) δ 7.83-7.81 (m, 2H), 7.51-7.42 (m, 3H), 5.51 (dd, *J* = 25.6, 3.3 Hz, 1H), 4.05-3.95 (m, 4H), 1.20-1.17 (m, 6H); ^{13}C NMR (101 MHz, $CDCl_3$) δ 169.8 (dd, *J* = 272.8, 26.0 Hz), 131.5, 130.4 (d, *J* = 28.7 Hz), 128.83 128.78, 128.2, 95.2 (dd, *J* = 202.3, 22.5 Hz), 62.3, 62.2, 16.23, 16.16; ^{19}F NMR (376 MHz, $CDCl_3$) δ -66.9 (dd, *J* = 72.9, 25.6 Hz, 1F); ^{31}P NMR (162 MHz, $CDCl_3$) δ 15.5 (d, *J* = 72.7 Hz, 1P); HR-MS *m/z*: Calcd for $C_{12}H_{17}FO_3P$ $[M + H]^+$ calcd. 259.0905 found 259.0896

Diethyl (*Z*)-(2-fluoro-2-(*p*-tolyl)vinyl)phosphonate (2b)

The reaction was conducted on a 0.2 mmol scale following the general procedure. The desired product was purified by column chromatography (PE/EA 10:1) to give a colorless oil (37.0 mg, 68%).

^{1}H NMR (400 MHz, $CDCl_3$) δ 7.72 (d, *J* = 8.0 Hz, 2H), 7.23 (d, *J* = 8.0 Hz, 2H), 5.44 (dd, *J* = 26.0, 3.6 Hz, 1H), 4.05-3.96 (m, 4H), 2.39 (s, 3H), 1.20 (t, J = 7.0 Hz, 6H);

^{13}C NMR (101 MHz, $CDCl_3$) δ 170.1 (dd, *J* = 272.0, 26.3Hz), 142.0, 128.9, 128.7 (d, *J* =6.0 Hz), 127.5 (d, *J* = 29.4 Hz), 94.3 (dd, *J* = 203.1, 23.2 Hz), 62.2, 62.1, 21.7, 16.24, 16.17; ^{19}F (376 MHz, $CDCl_3$) δ -67.0 (dd, *J* = 73.7, 25.9 Hz, 1F); ^{31}P (162 MHz, $CDCl_3$) δ 15.9 (d, *J* = 72.7 Hz, 1P); HR-MS *m/z*: Calcd for $C_{13}H_{19}FO_3P$ $[M + H]^+$ calcd. 273.1061 found 273.1053

Diethyl (*Z*)-(2-fluoro-2-(*m*-tolyl)vinyl)phosphonate (2c)

The reaction was conducted on a 0.2 mmol scale following the general procedure. The desired product was purified by column chromatography (PE:EA=10:1) to give a colorless oil (36.4 mg, 67%).

^1H NMR (400 MHz, $CDCl_3$) δ 7.63-7.61 (m, 2H), 7.34-7.30 (m, 2H), 5.48 (dd, *J* = 25.2, 3.2 Hz, 1H), 4.05-3.95 (m, 4H), 2.39 (s, 3H), 1.19 (t, *J* = 7.0 Hz, 6H); ^{13}C NMR (101 MHz, $CDCl_3$) δ170.1 (dd, *J* = 272.9, 26.1 Hz), 137.9, 132.3, 130.4 (d, *J* = 28.9 Hz), 129.2 (d, *J* = 5.8Hz), 128.1, 126.1 (d, *J* = 6.2 Hz), 95.1 (dd, *J* = 203.3, 22.6 Hz), 62.22, 62.16, 21.5, 16.25, 16.19; ^{19}F (376 MHz, $CDCl_3$) δ -66.6 (dd, *J* = 72.9, 25.2 Hz, 1F); ^{31}P (162 MHz, $CDCl_3$) δ 15.7 (d, *J* = 73.2 Hz, 1P); HR-MS *m/z*: Calcd for $C_{13}H_{19}FO_3P$ $[M + H]^+$ calcd. 273.1061 found 273.1050

Diethyl (*Z*)-(2-(4-(*tert*-butyl)phenyl)-2-fluorovinyl)phosphonate (2d)

The reaction was conducted on a 0.2 mmol scale following the general procedure. The desired product was purified by column chromatography (PE/EA 10:1) to give a colorless oil (47.1 mg, 75%).

^1H NMR (400 MHz, $CDCl_3$) δ 7.77-7.74 (m, 2H), 7.46-7.44 (m, 2H), 5.45 (dd, *J* = 25.6, 3.2 Hz, 1H), 4.05-3.96 (m, 4H), 1.33 (s, 9H), 1.19 (t, *J* = 7.0 Hz, 6H); ^{13}C NMR (101 MHz, $CDCl_3$) δ 170.1 (dd, *J* = 271.8, 26.2 Hz), 155.1, 128.6 (d, *J* = 6.1 Hz), 127.4 (dd, *J* = 28.8, 1.4 Hz), 125.2, 94.2 (dd, *J* = 202.6, 23.0 Hz), 62.2, 62.1, 35.1, 31.2, 16.2, 16.1; ^{19}F (376 MHz, $CDCl_3$) δ -67.2 (dd, *J* = 73.3, 25.6 Hz, 1F); ^{31}P (162 MHz, $CDCl_3$) δ 15.8 (d, *J* = 73.4 Hz, 1P); HR-MS *m/z*: Calcd for $C_{16}H_{25}FO_3P$ $[M + H]^+$ calcd. 315.1531 found 315.1543

Diethyl (*Z*)-(2-fluoro-2-(4-methoxyphenyl)vinyl)phosphonate (2e)

The reaction was conducted on a 0.2 mmol scale following the general procedure. The desired product was purified by column chromatography (PE/EA 5:1) to give a colorless oil (34.6 mg, 60%).

^1H NMR (400 MHz, $CDCl_3$) δ 7.81 (d, *J* = 8.8 Hz, 2H), 6.93 (d, *J* = 8.8 Hz, 2H), 5.38 (dd, *J* = 26.0, 3.2 Hz, 1H), 4.05-3.98 (m, 4H), 3.85 (s, 3H), 1.22 (t, *J* = 7.0 Hz, 6H); ^{13}C NMR (101 MHz, $CDCl_3$) δ 169.8 (dd, J = 270.5, 26.5Hz), 162.1, 130.6 (d, *J* = 6.7 Hz), 122.6 (dd, *J* = 29.2, 1.4 Hz), 113.6, 92.9 (dd, *J* = 202.9, 23.7 Hz), 62.2, 62.1, 55.5, 16.3, 16.2; ^{19}F (376 MHz, $CDCl_3$) δ -67.4 (dd, *J* = 74.1, 26.3 Hz, 1F); ^{31}P (162

MHz, $CDCl_3$) δ 16.3 (d, *J* = 74.0 Hz, 1P); HR-MS *m/z*: Calcd for $C_{13}H_{19}FO_4P$ $[M + H]^+$ calcd. 289.1010 found 289.1001

Diethyl (*Z*)-(2-([1,1'-biphenyl]-4-yl)-2-fluorovinyl)phosphonate (2f)

The reaction was conducted on a 0.2 mmol scale following the general procedure. The desired product was purified by column chromatography (PE/EA 5:1) to give a colorless oil (46.1 mg, 69%).

^{1}H NMR (400 MHz, $CDCl_3$) δ 7.92 (d, *J* = 8.0 Hz, 2H), 7.68-7.61 (m, 4H), 7.49-7.45 (m, 2H), 7.41-7.37 (m, 1H), 5.52 (dd, *J* = 26.0, 3.2 Hz, 1H), 4.08-4.00 (m, 4H), 1.22 (t, *J* = 7.0 Hz, 6H); ^{13}C NMR (101 MHz, $CDCl_3$) δ 169.6 (dd, J = 271.4, 26.1Hz), 144.2, 140.1, 129.3 (d, *J* = 6.3 Hz), 129.1, 128.2, 127.3, 126.9, 95.0 (dd, *J* = 202.4, 22.8 Hz), 62.31, 62.25, 16.3, 16.2; ^{19}F (376 MHz, $CDCl_3$) δ -67.8 (dd, *J* = 72.9, 25.9 Hz, 1F); ^{31}P (162 MHz, $CDCl_3$) δ 15.6 (d, *J* = 72.9 Hz, 1P); HR-MS *m/z*: Calcd for $C_{18}H_{21}FO_3P$ $[M + H]^+$ calcd. 335.1218 found 335.1209

Diethyl (*Z*)-(2-fluoro-2-(4-fluorophenyl)vinyl)phosphonate (2g)

The reaction was conducted on a 0.2 mmol scale following the general procedure. The desired product was purified by column chromatography (PE/EA 10:1) to give a colorless oil (42.5 mg, 77%).

^{1}H NMR (400 MHz, $CDCl_3$) δ7.87-7.84 (m, 2H), 7.14-7.10 (m, 2H), 5.49 (dd, *J* = 25.6, 2.8 Hz, 1H), 4.06-3.97 (m, 4H), 1.22 (t, *J* = 7.0 Hz, 6H); ^{13}C NMR (101 MHz, $CDCl_3$) δ 168.7(dd, *J* = 271.6, 26.0Hz), 164.6 (dd, *J* = 253.9, 1.1Hz), 131.2 (ddd, *J* = 8.8, 6.3, 0.8 Hz), 126.6 (ddd, J = 29.5, 3.2, 1.6Hz), 115.5 (d, *J* = 22.1 Hz), 95.2 (dd, *J* = 202.3, 22.6Hz), 62.3, 62.2, 16.3, 16.2; ^{19}F (376 MHz, $CDCl_3$) -107.43- -107.36 (m, 1F), δ -66.8 (dd, *J* = 72.6, 25.6 Hz, 1F); ^{31}P (162 MHz, $CDCl_3$) δ 15.3 (d, *J* = 72.6 Hz, 1P); HR-MS *m/z*: Calcd for $C_{12}H_{16}F_2O_3P$ $[M + H]^+$calcd. 277.0811 found 277.0800

Diethyl (*Z*)-(2-fluoro-2-(3-fluorophenyl)vinyl)phosphonate (2h)

The reaction was conducted on a 0.2 mmol scale following the general procedure. The desired product was purified by column chromatography (PE/EA 10:1) to give a colorless oil (44.1 mg, 80%).

^{1}H NMR (400 MHz, $CDCl_3$) δ7.66 (d, *J* = 8.0 Hz, 1H), 7.54 (dt, *J* = 11.6, 1.6 Hz, 1H), 7.44-7.39(m, 1H), 7.19 (td, *J* = 8.4, 2.4 Hz, 1H), 5.56 (dd, *J* = 25.6, 2.8 Hz, 1H), 4.08-3.99 (m, 4H), 1.22 (t, *J* = 7.2 Hz, 6H); ^{13}C NMR (101 MHz, $CDCl_3$) 168.1 (ddd, *J* = 272.1, 25.5, 2.5Hz), 162.2 (d, *J* = 247.8 Hz), 132.3 (ddd, *J* = 29.7, 8.2, 1.5 Hz), 130.8(d, *J* = 8.8 Hz), 129.9 (d, *J* = 8.1Hz), 124.8 (q, *J* = 2.3 Hz), 118.5 (d, *J* =21.2 Hz), 115.8 (dd, *J* = 327.2, 21.6 Hz), 115.8 (dd, *J* = 24.0, 6.0 Hz), 96.4 (dd, *J* = 202.1, 21.9Hz), 62.4, 62.3, 16.3, 16.2; ^{19}F (376 MHz, $CDCl_3$) -112.27-112.20 (m, 1F), δ -67.9 (dd, *J* = 71.8, 25.6 Hz, 1F); ^{31}P (162 MHz, $CDCl_3$) δ 14.7 (d, *J* = 71.9 Hz, 1P);

HR-MS *m/z*: Calcd for $C_{12}H_{16}F_2O_3P$ [M + H] $^+$calcd. 277.0811 found 277.0796

Diethyl (*Z*)-(2-(4-chlorophenyl)-2-fluorovinyl)phosphonate (2i)

The reaction was conducted on a 0.2 mmol scale following the general procedure. The desired product was purified by column chromatography (PE/EA 10:1) to give a colorless oil (48.5 mg, 83%).

^{1}H NMR (400 MHz, $CDCl_3$) δ7.79 (d, *J* = 8.4 Hz, 2H), 7.41 (d, *J* = 8.4 Hz, 2H), 5.52 (dd, *J* = 26.0, 2.8 Hz, 1H), 4.07-3.98 (m, 4H), 1.23 (t, *J* = 7.0 Hz, 6H); ^{13}C NMR (101 MHz, $CDCl_3$) δ 168.6 (dd, *J* = 271.6, 25.9 Hz), 137.8, 130.2 (dd, *J* = 6.1, 0.8 Hz), 128.8 (dd, *J* = 29.5, 1.7 Hz), 128.6, 95.8 (dd, *J* = 202.0, 22.2 Hz), 62.4, 62.3, 16.3, 16.2; ^{19}F (376 MHz, $CDCl_3$) δ -67.9 (dd, *J* = 71.8, 25.6 Hz, 1F); ^{31}P (162 MHz, $CDCl_3$) δ 15.0 (d, *J* = 72.1Hz, 1P); HR-MS *m/z*: Calcd for $C_{12}H_{16}ClFO_3P$ [M + H] $^+$calcd. 293.0515 found 293.0521

Diethyl (*Z*)-(2-(3-chlorophenyl)-2-fluorovinyl)phosphonate (2j)

The reaction was conducted on a 0.2 mmol scale following the general procedure. The desired product was purified by column chromatography (PE/EA 10:1) to give a colorless oil (41.5 mg, 71%).

^{1}H NMR (400 MHz, $CDCl_3$) δ7.79-7.74 (m, 2H), 7.47-7.45 (m, 1H), 7.40-7.36(m, 1H), 5.56 (dd, *J* = 25.6, 2.0 Hz, 1H), 4.07-3.97 (m, 4H), 1.23 (t, *J* = 7.0 Hz, 6H); ^{13}C NMR (101 MHz, $CDCl_3$) δ 168.1 (dd, *J* = 272.6, 25.4 Hz), 134.3, 132.0 (dd, *J* = 29.4, 1.3 Hz), 131.5, 129.6, 127.9 (dd, *J* = 160.5, 6.1Hz), 96.6 (dd, *J* = 202.1, 21.7 Hz), 62.4, 62.4, 16.3, 16.2; ^{19}F (376 MHz, $CDCl_3$) δ -68.1 (dd, *J* = 71.4, 25.6 Hz, 1F); ^{31}P (162 MHz, $CDCl_3$) δ 14.7 (d, *J* = 71.6Hz, 1P); HR-MS *m/z*: Calcd for $C_{12}H_{16}ClFO_3P$ [M + H] $^+$calcd. 293.0515 found 293.0505

Diethyl (*Z*)-(2-(2-chlorophenyl)-2-fluorovinyl)phosphonate (2k)

The reaction was conducted on a 0.2 mmol scale following the general procedure. The desired product was purified by column chromatography (PE/EA 10:1) to give a colorless oil (32.7 mg, 56%).

^{1}H NMR (400 MHz, $CDCl_3$) δ7.57-7.56 (m, 1H), 7.46-7.40 (m, 2H), 7.35-7.31(m, 1H), 5.68 (dd, *J* = 20.8, 5.2 Hz, 1H), 4.02-3.86 (m, 4H), 1.17 (t, *J* = 7.2 Hz, 6H); ^{13}C NMR (101 MHz, $CDCl_3$) δ 168.1 (dd, *J* = 279.0, 24.8Hz), 133.3, 132.4, 132.1 (d, *J* = 2.3 Hz), 130.5 (dd, *J* = 25.6, 1.7 Hz), 129.6 (d, *J* = 1.9 Hz), 99.4 (dd, *J* = 200.5, 19.7 Hz), 62.14, 62.10, 16.22, 16.15; ^{19}F (376 MHz, $CDCl_3$) δ -69.1 (dd, *J* = 66.6, 21.1 Hz, 1F); ^{31}P (162 MHz, $CDCl_3$) δ 13.7 (d, *J* = 66.7 Hz, 1P); HR-MS *m/z*: Calcd for $C_{12}H_{16}ClFO_3P$ [M + H] $^+$calcd. 293.0515 found 293.0507

Diethyl (*Z*)-(2-(4-bromophenyl)-2-fluorovinyl)phosphonate (2l)

The reaction was conducted on a 0.2 mmol scale following the general procedure. The

desired product was purified by column chromatography (PE/EA 10:1) to give a colorless oil (45.7 mg, 68%).

^{1}H NMR (400 MHz, $CDCl_3$) δ7.72 (d, *J* = 8.8 Hz, 2H), 7.57 (d, *J* = 8.8 Hz, 2H), 5.53 (dd, *J* = 25.6, 2.8 Hz, 1H), 4.07-3.98 (m, 4H), 1.23 (t, *J* = 7.0 Hz, 6H); ^{13}C NMR (101 MHz, $CDCl_3$) δ 168.6 (dd, *J* = 271.7, 25.6 Hz), 131.6, 130.4 (dd, *J* = 6.1, 0.8Hz), 129.2 (dd, *J* = 29.5, 1.7 Hz), 126.2 (d, *J* = 1.3Hz), 96.0 (dd, *J* = 202.0, 22.2 Hz), 62.4, 62.3, 16.3, 16.2; ^{19}F (376 MHz, $CDCl_3$) δ -68.2 (dd, *J* = 71.8, 25.6 Hz, 1F); ^{31}P (162 MHz, $CDCl_3$) δ 15.0 (d, *J* = 72.1Hz, 1P)； HR-MS *m/z*: Calcd for $C_{12}H_{16}BrFO_3P$ [M + H]$^{+}$ calcd. 336.9926 found 236.9941

Diethyl (*Z*)-(2-fluoro-2-(4-(trifluoromethyl)phenyl)vinyl)phosphonate (2m)

The reaction was conducted on a 0.2 mmol scale following the general procedure. The desired product was purified by column chromatography (PE/EA 10:1) to give a colorless oil (55.4 mg, 85%).

^{1}H NMR (400 MHz, $CDCl_3$) δ7.96 (d, *J* = 8.4 Hz, 2H), 7.70 (d, *J* = 8.0 Hz, 2H), 5.63 (dd, *J* = 25.6, 2.4 Hz, 1H), 4.07-4.00(m, 4H), 1.22 (t, *J* = 7.2 Hz, 6H); ^{13}C NMR (101MHz, $CDCl_3$) δ 168.0 (dd, *J* = 272.5 25.6 Hz), 133.4 (d, *J* = 66.4, 29.6 Hz), 129.3 (d, *J* = 5.6 Hz), 125.2 (q, *J* = 3.7 Hz), 123.7 (q, *J* = 273.8 Hz), 97.5 (dd, *J* = 201.6, 21.3 Hz), 62.5, 62.4, 16.3, 16.2; ^{19}F (376 MHz, $CDCl_3$) δ -63.2 (s, 1F), -68.2 (dd, *J* = 71.1, 25.6 Hz, 1F); ^{31}P (162 MHz, $CDCl_3$) δ 14.3 (d, *J* = 71.1Hz, 1P); HR-MS *m/z*: Calcd for $C_{13}H_{16}F_4O_3P$ [M + H]$^{+}$ calcd. 327.0779 found 327.0789

Diethyl (*Z*)-(2-(4-cyanophenyl)-2-fluorovinyl)phosphonate (2n)

The reaction was conducted on a 0.2 mmol scale following the general procedure. The desired product was purified by column chromatography (PE/EA 3:1) to give a colorless oil (39.1 mg, 69%).

^{1}H NMR (400 MHz, $CDCl_3$) δ 7.97 (d, *J* = 8.4 Hz, 2H), 7.73 (d, *J* = 8.0 Hz, 2H), 5.66 (dd, *J* = 25.6, 2.0 Hz, 1H), 4.07- 3.99 (m, 4H), 3.95 (s, 3H), 1.21 (t, *J* = 7.2 Hz, 6H); ^{13}C NMR (101 MHz, $CDCl_3$) δ 168.8 (d, *J* = 348.45 Hz), 134.1, 131.8, 129.3 (d, *J* = 5.05 Hz), 117.9, 114.8, 98.1 (dd, *J* = 201.0, 21.2 Hz), 62.42, 62.36, 16.14, 16.07; ^{19}F (376 MHz, $CDCl_3$) δ -69.6 (dd, *J* = 70.3, 25.6 Hz, 1F); ^{31}P (162 MHz, $CDCl_3$) δ 14.3 (d, *J* = 71.1Hz, 1P); HR-MS *m/z*: Calcd for $C_{13}H_{16}FNO_3P$ [M + H]$^{+}$calcd. 284.0857 found 2 284.0861

Methyl (*Z*)-4-(2-(diethoxyphosphoryl)-1-fluorovinyl)benzoate (2o)

The reaction was conducted on a 0.2 mmol scale following the general procedure. The desired product was purified by column chromatography (PE/EA 1:1) to give a colorless oil (46.8 mg, 74%).

^{1}H NMR (400 MHz, $CDCl_3$) δ 8.10 (d, *J* = 8.4 Hz, 2H), 7.91 (d, *J* = 8.4 Hz, 2H), 5.61

(dd, J = 25.6, 2.8 Hz, 1H), 4.08-4.01(m, 4H), 1.24 (t, J = 7.0 Hz, 6H); ^{13}C NMR (101 MHz, $CDCl_3$) δ 168.4 (dd, J = 272.5, 25.5 Hz), 166.3, 134.4 (dd, J = 28.0, 1.1 Hz), 132.6, 129.3, 128.9 (d, J = 5.8 Hz), 97.2 (dd, J = 202.0, 21.7 Hz), 62.4, 62.3, 52.5, 16.3, 16.2; ^{19}F (376 MHz, $CDCl_3$) δ -68.3 (dd, J = 71.4, 25.6 Hz, 1F); ^{31}P (162 MHz, $CDCl_3$) δ 14.5 (d, J = 71.4Hz, 1P); HR-MS *m/z*: Calcd for $C_{14}H_{19}FO_5P$ $[M + H]^+$ calcd. 317.0960 found 317.0957

Diethyl (*Z*)-(1-fluoro-1-phenylprop-1-en-2-yl)phosphonate (2p)

The reaction was conducted on a 0.2 mmol scale following the general procedure. The desired product was purified by column chromatography (PE/EA 3:1) to give a colorless oil (32.6 mg, 60%).

^{1}H NMR (400 MHz, $CDCl_3$) δ 7.60 (d, J = 7.2 Hz, 2H), 7.45-7.37 (m, 3H), 4.00-3.82 (m, 4H), 2.05-2.01 (m, 3H), 1.12 (td, J = 6.8, 1.2 Hz, 6H); ^{13}C NMR (101 MHz, $CDCl_3$) δ 165.2 (dd, J = 270.4, 33.5 Hz), 132.1 (d, J = 28.9 Hz), 130.4, 129.4, 129.3, 127.8, 104.8 (dd, J = 193.1, 17.8 Hz), 61.94, 61.88, 16.1, 16.0, 12.5 (dd, J = 9.9, 5.1 Hz); ^{19}F (376 MHz, $CDCl_3$) δ -67.8 (d, J = 54.1 Hz, 1F); ^{31}P (162 MHz, $CDCl_3$) δ 18.7 (d, J = 53.8 Hz, 1P); HR-MS *m/z*: Calcd for $C_{13}H_{19}FO_3P$ $[M + H]^+$calcd. 273.1061 found 273.1060

Diethyl (*Z*)-(1-fluoro-1-phenylbut-1-en-2-yl)phosphonate (2q)

The reaction was conducted on a 0.2 mmol scale following the general procedure. The desired product was purified by column chromatography (PE/EA 3:1) to give a colorless oil (29.2 mg, 51%).

^{1}H NMR (400 MHz, $CDCl_3$) δ 7.61 (d, J = 7.2 Hz, 2H), 7.43-7.39 (m, 3H), 3.99-3.84 (m, 4H), 2.52-2.46 (m, 2H), 1.22 (t, J = 7.4 Hz, 3H) 1.11 (t, J = 7.0 Hz, 6H); ^{13}C NMR (101 MHz, $CDCl_3$) δ165.7 (dd, J = 270.5, 34.4 Hz), 132.2 (dd, J = 28.9, 1.6 Hz), 130.4 (d, J = 2.1 Hz), 129.4 (dd, J = 3.8, 0.9 Hz), 127.8, 111.3 (dd, J = 190.0, 6.8 Hz), 61.9, 61.8, 21.0 (dd, J = 8.2, 5.2 Hz), 16.14, 16.07, 14.3; ^{19}F (376 MHz, $CDCl_3$) δ -70.4 (d, J = 55.6 Hz, 1F); ^{31}P (162 MHz, $CDCl_3$) δ 19.2 (d, J = 55.9 Hz, 1P); HR-MS *m/z*: Calcd for $C_{14}H_{21}FO_3P$ $[M + H]^+$calcd. 287.1218 found 287.1219

Dimethyl (*Z*)-(2-fluoro-2-phenylvinyl)phosphonate (3a)

The reaction was conducted on a 0.2 mmol scale following the general procedure. The desired product was purified by column chromatography (PE/EA 5:1) to give a colorless oil (34.5 mg, 75%).

^{1}H NMR (400 MHz, $CDCl_3$) δ 7.81 (d, J = 7.6 Hz, 2H), 7.52-7.42 (m, 3H), 5.48 (dd, J = 25.2, 2.0 Hz, 1H), 3.63 (d, J = 11.6Hz, 6H); ^{13}C NMR (101 MHz, $CDCl_3$) δ 170.4 (dd, J = 273.4, 26.6 Hz), 131.7, 130.2 (d, J = 28.8 Hz), 128.7 (d, J = 6.1 Hz), 128.3, 93.8 (dd, J = 203.7, 23.3 Hz), 52.72, 52.66; ^{19}F (376 MHz, $CDCl_3$) δ -65.8 (dd, J =

72.9, 25.2 Hz, 1F); ^{31}P (162 MHz, $CDCl_3$) δ 18.5 (d, *J* = 73.4Hz, 1P); HR-MS *m/z*: Calcd for $C_{10}H_{13}FO_3P$ $[M + H]^+$ calcd. 231.0592 found 231.0595

Dimethyl (*Z*)-(2-fluoro-2-(*p*-tolyl)vinyl) phosphonate (3b)

The reaction was conducted on a 0.2 mmol scale following the general procedure. The desired product was purified by column chromatography (PE/EA 5:1) to give a colorless oil (29.3 mg, 60%).

^{1}H NMR (400 MHz, $CDCl_3$) δ 7.71 (d, *J* = 8.4 Hz, 2H), δ 7.24 (d, *J* = 8.0 Hz, 2H), 5.42 (dd, *J* = 25.2, 3.2 Hz, 1H), 3.64 (d, *J* = 11.6Hz, 6H) 2.40 (s, 3H); ^{13}C NMR (101 MHz, $CDCl_3$) δ 170.6 (dd, *J* = 272.7, 26.8 Hz), 142.2, 129.0, 128.6 (d, *J* = 6.3 Hz), 127.3 (dd, *J* = 28.8, 1.5 Hz), 92.8 (dd, *J* = 204.0, 23.9 Hz), 52.7, 52.6, 21.7; ^{19}F (376 MHz, $CDCl_3$) δ -65.9 (dd, *J* = 74.1, 25.6 Hz, 1F); ^{31}P (162 MHz, $CDCl_3$) δ 18.9 (d, *J* = 74.0Hz, 1P); HR-MS *m/z*: Calcd for $C_{11}H_{15}FO_3P$ $[M + H]^+$calcd.245.0748 found 245.0743

Dimethyl (*Z*)-(2-fluoro-2-(4-fluorophenyl)vinyl) phosphonate (3c)

The reaction was conducted on a 0.2 mmol scale following the general procedure. The desired product was purified by column chromatography (PE/EA 5:1) to give a colorless oil (34.2 mg, 69%).

^{1}H NMR (400 MHz, $CDCl_3$) δ 7.66 (d, *J* = 7.6 Hz, 1H), 7.52 (d, *J* = 9.2 Hz, 1H), δ 7.45-7.40 (m, 1H), 7.22-7.18 (m, 1H), 5.57-5.51 (m, 1H), 3.67(d, *J* = 11.2Hz, 6H);^{13}C NMR (100 MHz, $CDCl_3$) δ168.6 (ddd, *J* =271.8, 25.9, 2.6 Hz), 162.3 (d, *J* = 248.1 Hz), 132.1 (ddd, *J* =29.3, 8.2, 1.6 Hz), 130.0 (d, *J* =8.2 Hz), 124.7(dd, *J* = 6.0, 3.1 Hz), 118.7(d, *J* = 21.2 Hz), 115.6 (dd, *J* = 24.0, 6.2 Hz), 95.1(dd, *J* =203.2, 22.6 Hz), 52.8, 52.7; ^{19}F (376 MHz, $CDCl_3$) δ-112.0 (m, 1F), -66.7 (dd, *J* = 72.2, 25.2 Hz, 1F); ^{31}P (162 MHz, $CDCl_3$) δ 17.1 (d, *J* = 72.3 Hz, 1P); HR-MS *m/z*: Calcd for $C_{10}H_{12}F_2O_3P$ $[M + H]^+$ calcd.249.0498 found 249.0501

Dimethyl (*Z*)-(2-(4-bromophenyl)-2-fluorovinyl)phosphonate (3d)

The reaction was conducted on a 0.2 mmol scale following the general procedure. The desired product was purified by column chromatography (PE/EA 5:1) to give a colorless oil (44.4 mg, 72%).

^{1}H NMR (400 MHz, $CDCl_3$) δ 7.96 (d, *J* = 8.4 Hz, 2H), δ 7.74 (d, *J* = 8.4 Hz, 2H), 5.64 (dd, *J* = 25.6, 2.0 Hz, 1H), 3.68(d, *J* = 11.6Hz, 6H); ^{13}C NMR (101 MHz, $CDCl_3$) δ 167.9 (dd, *J* = 272.9, 26.1 Hz), 134.2 (d, *J* = 29.4, 1.7 Hz), 132.1, 129.4 (d, *J* = 6.0, 0.7 Hz), 96.9 (dd, *J* = 202.1, 21.8Hz), 52.94, 52.88; ^{19}F (376 MHz, $CDCl_3$) δ -68.1 (dd, *J* = 71.1, 25.2 Hz, 1F); ^{31}P (162 MHz, $CDCl_3$) δ 16.8 (d, *J* = 71.1Hz, 1P); HR-MS *m/z*: Calcd for $C_{10}H_{12}BrFO_3P$ $[M + H]^+$calcd.308.9613 found 308.9625

2.1 Gram-scale reaction

In a vial, **1a** (816 mg, 8 mmol), Selectfluor® (5.6 g, 16 mmol), diethyl phosphonate (2.2 mg, 16 mmol), and silver acetate (132 mg, 0.8 mmol) were dissolved in DEC/H_2O 25 mL:25 mL. The resulting mixture was stirred under N_2 atmosphere at 60 °C for 48 h. After completion, the reaction was quenched and the residual oil was purified by column chromatography on silica gel, affording the product **2a** (1.4 g, yield 67%).

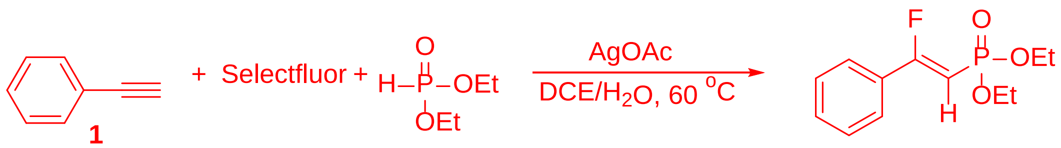

3 References

1. Okoromoba, O. -E.; Han, J. -B.; Hammond, G. B.; Xu, B. J. Am. Chem. Soc. **2014**, 136, 14381-14384

2. Xu, J.-J.; Burton, D.-J. J.Org.Chem. **2006**, 71, 3743-3747

4 Copies of NMR spectra

Diethyl (*Z*)-(2-fluoro-2-phenylvinyl)phosphonate (2a)

^{1}H NMR

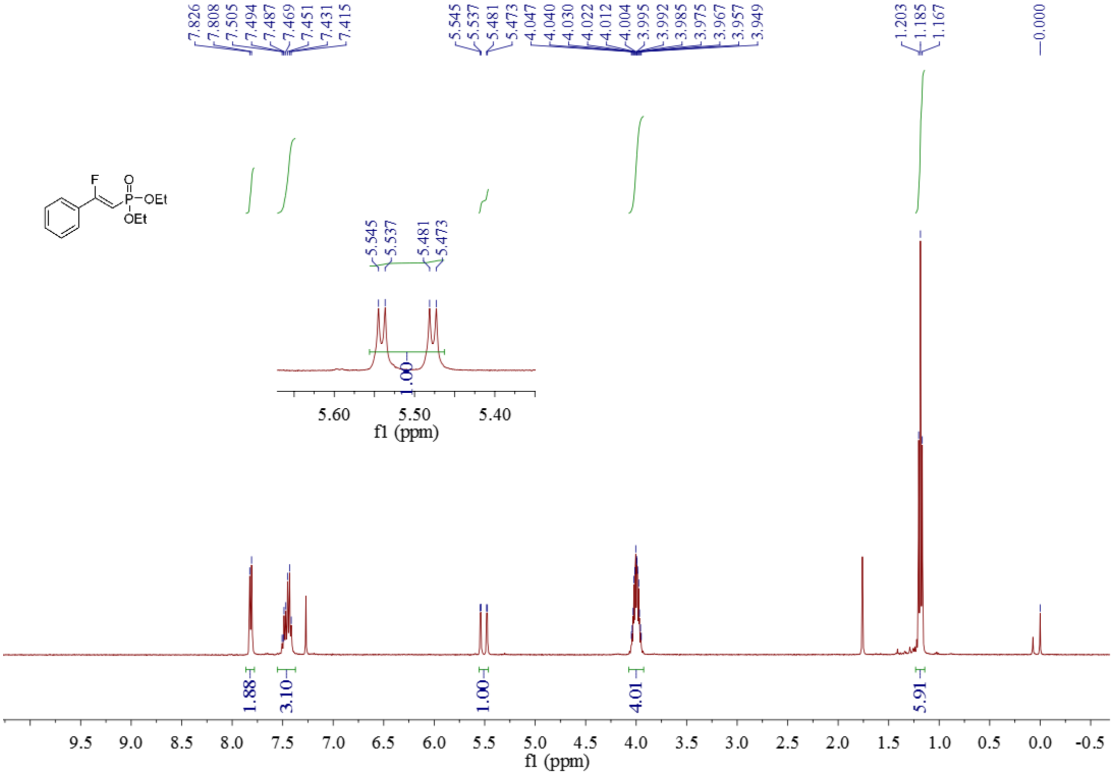

^{13}C NMR

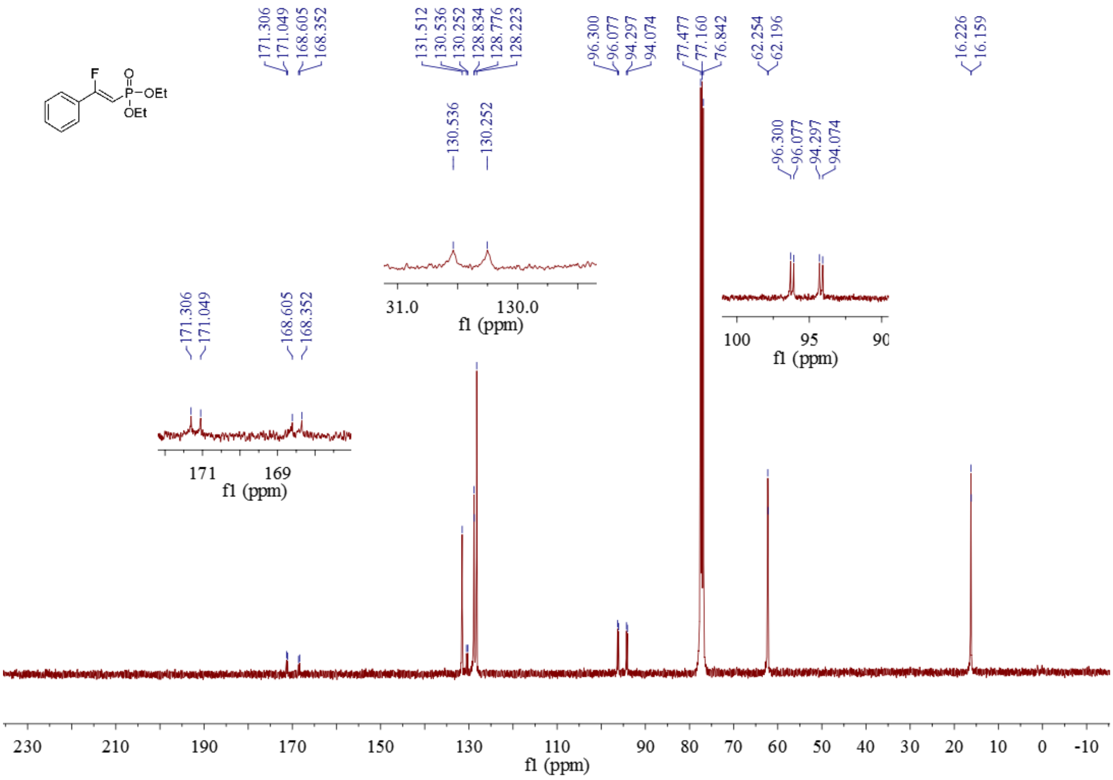

^{19}F NMR

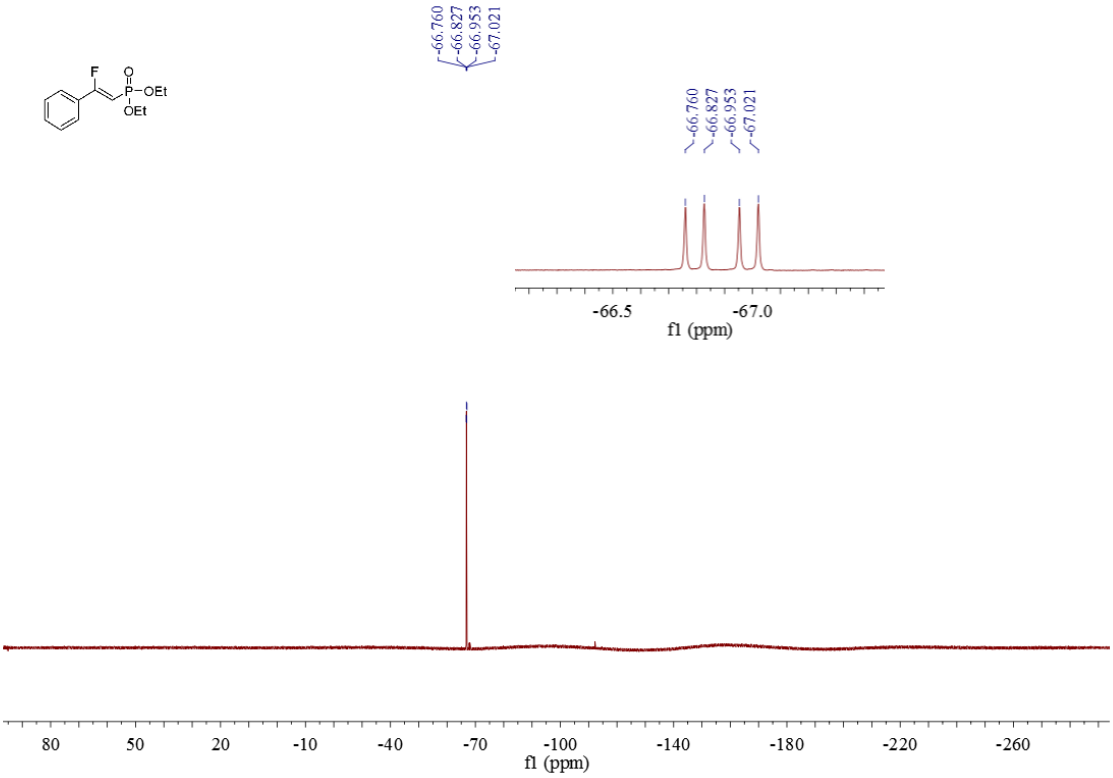

^{31}P NMR

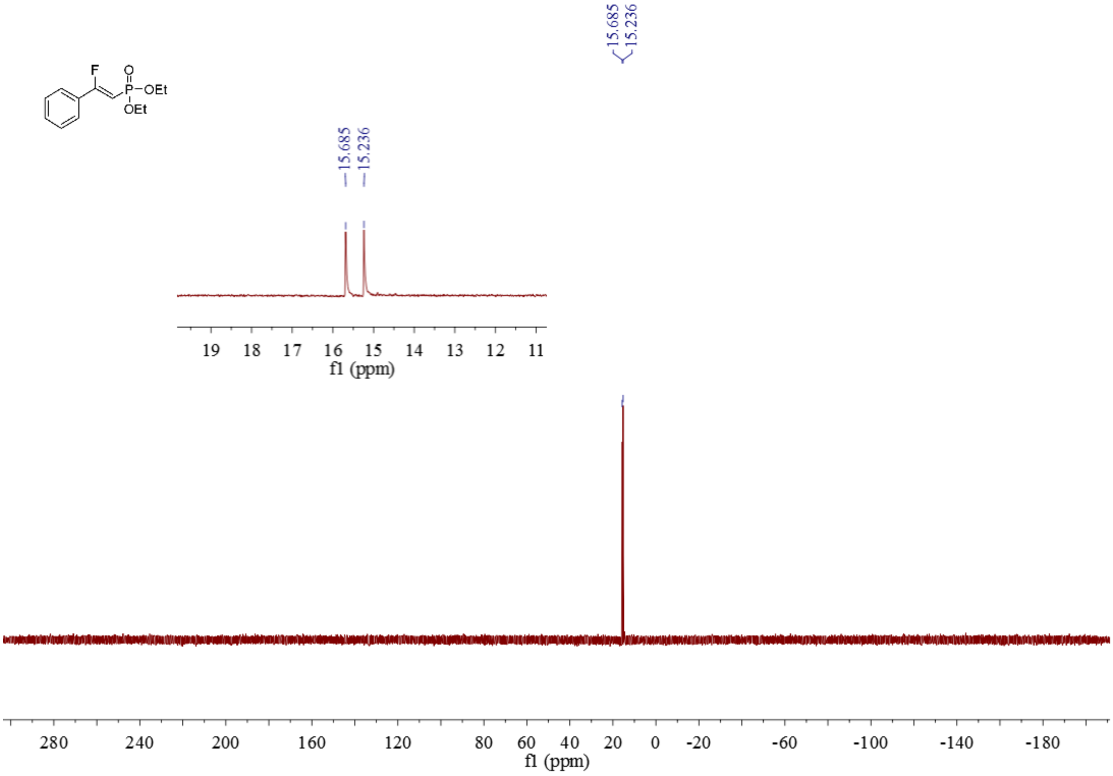

Diethyl (*Z*)-(2-fluoro-2-(*p*-tolyl)vinyl)phosphonate (2b)

^{1}H NMR

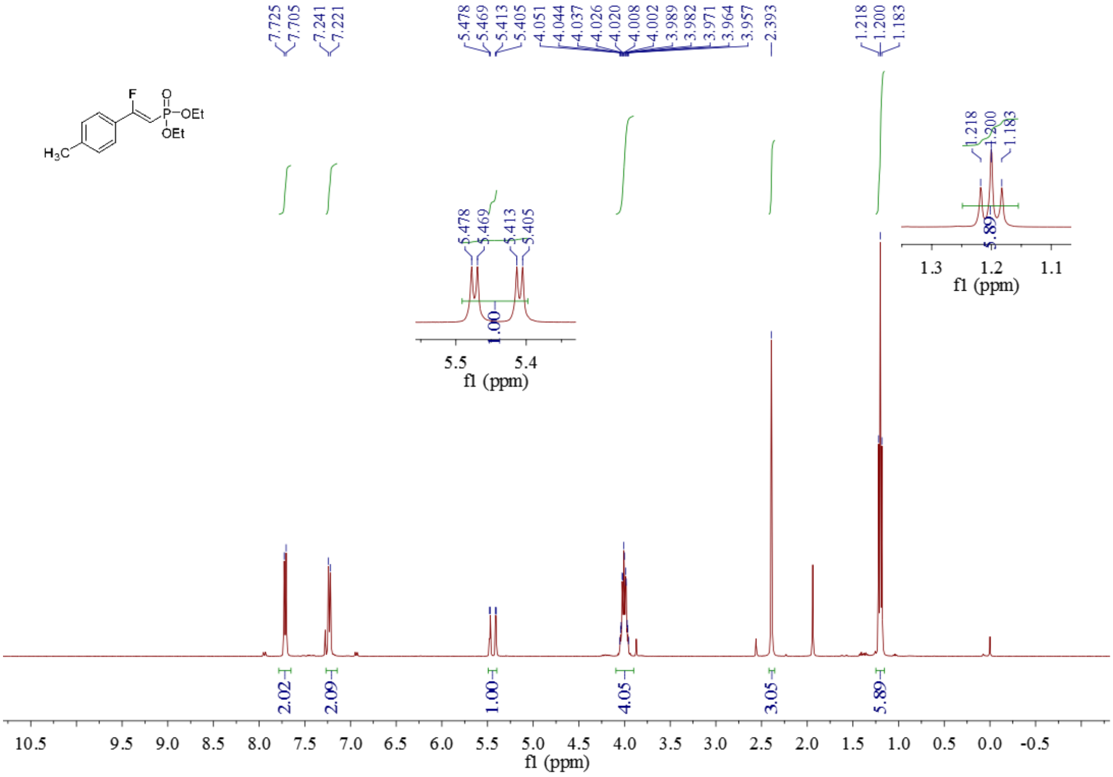

^{13}C NMR

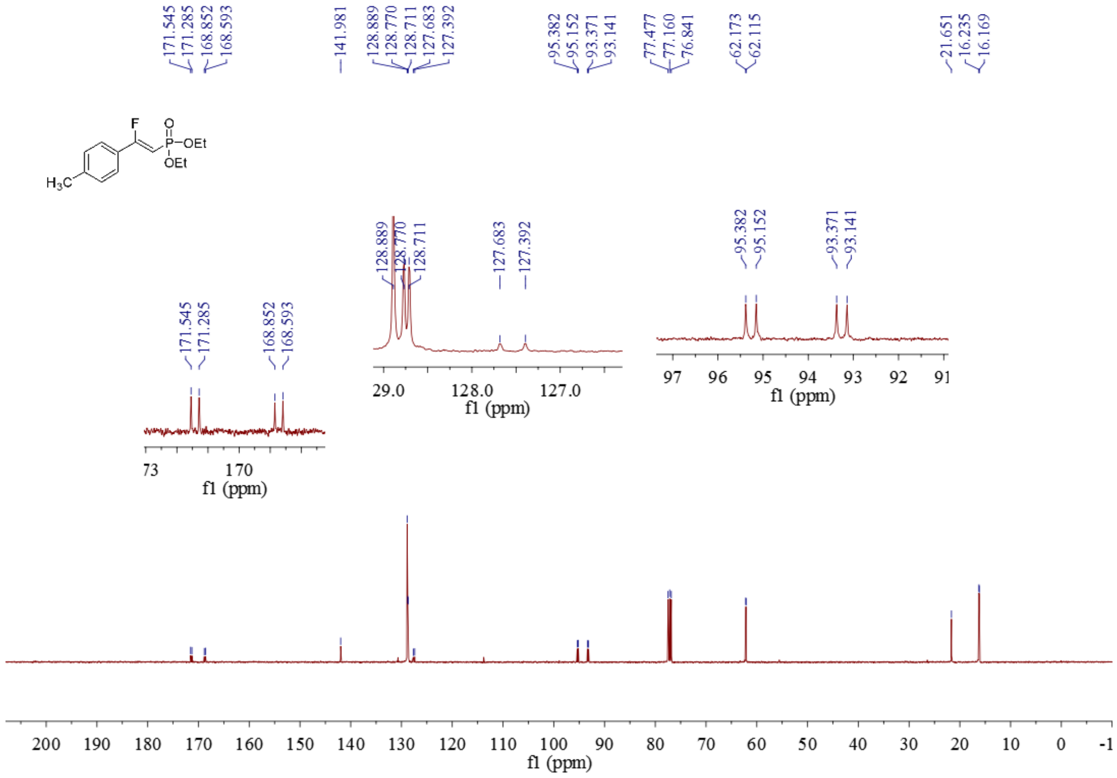

^{19}F NMR

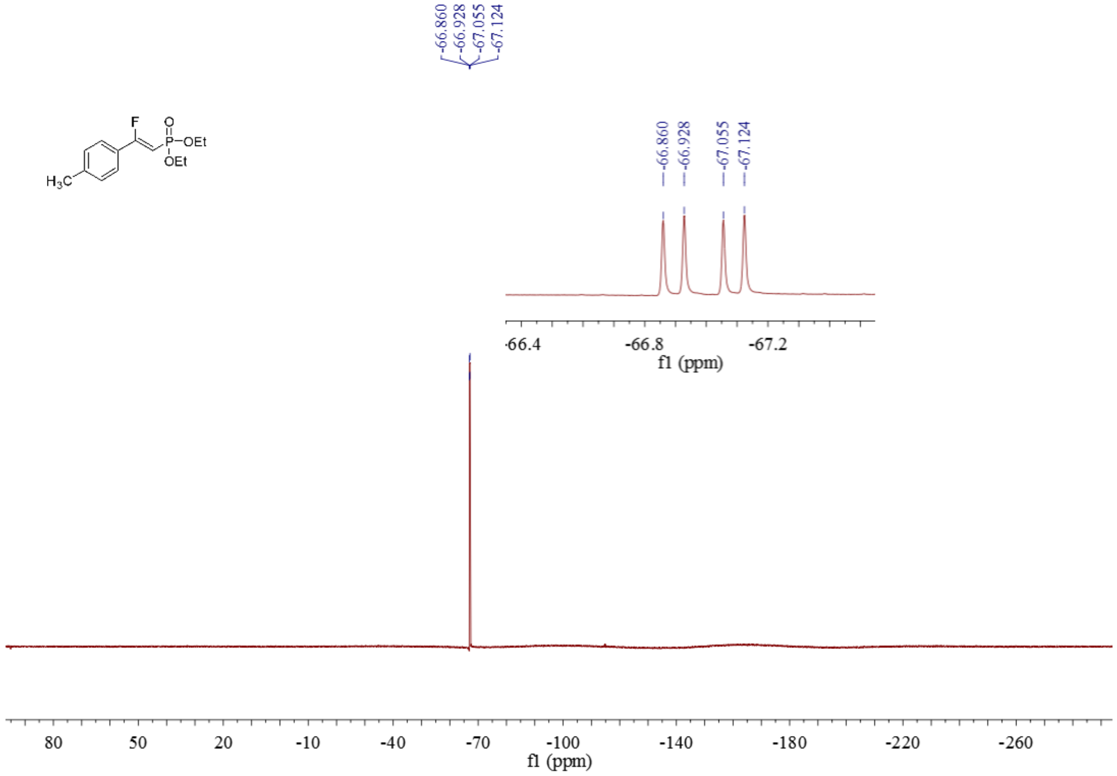

^{31}P NMR

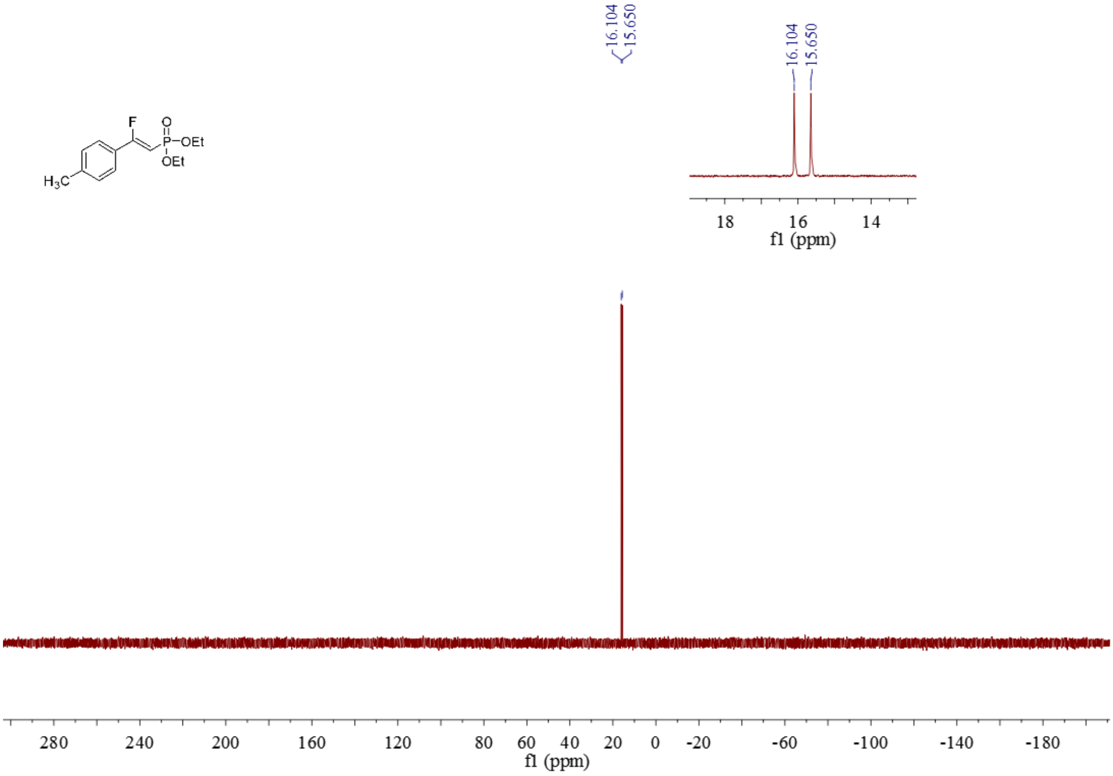

Diethyl (*Z*)-(2-fluoro-2-(*m*-tolyl)vinyl)phosphonate (2c)

^{1}H NMR

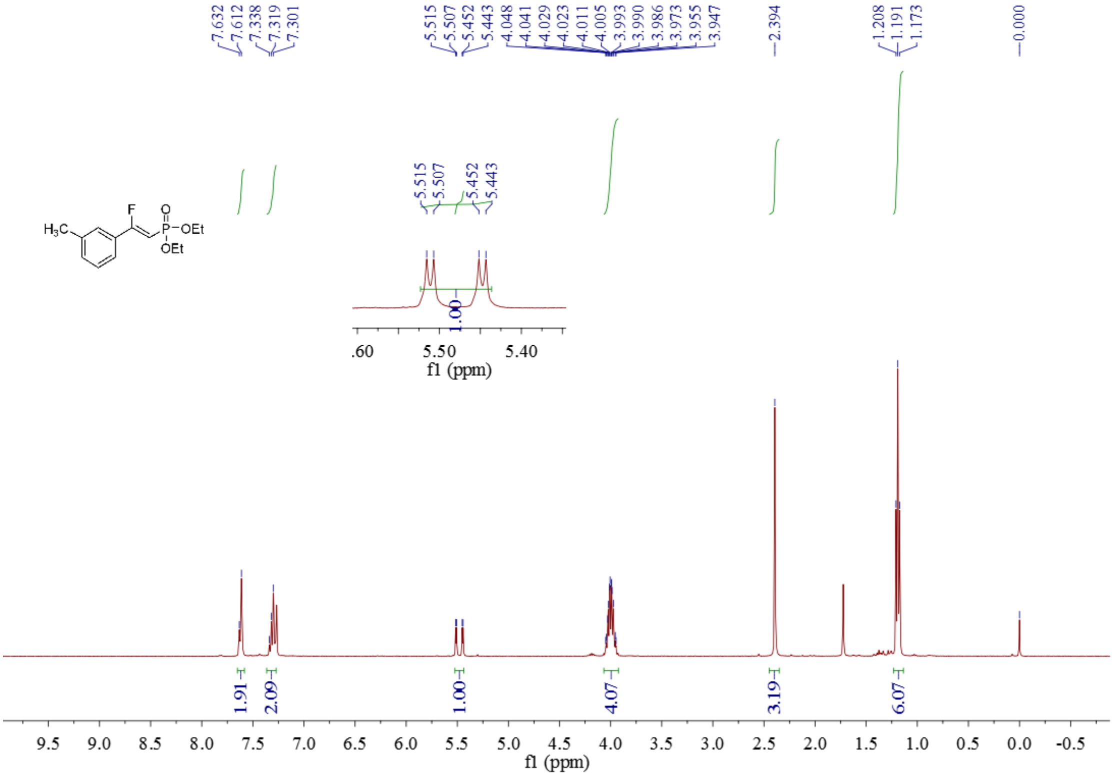

^{13}C NMR

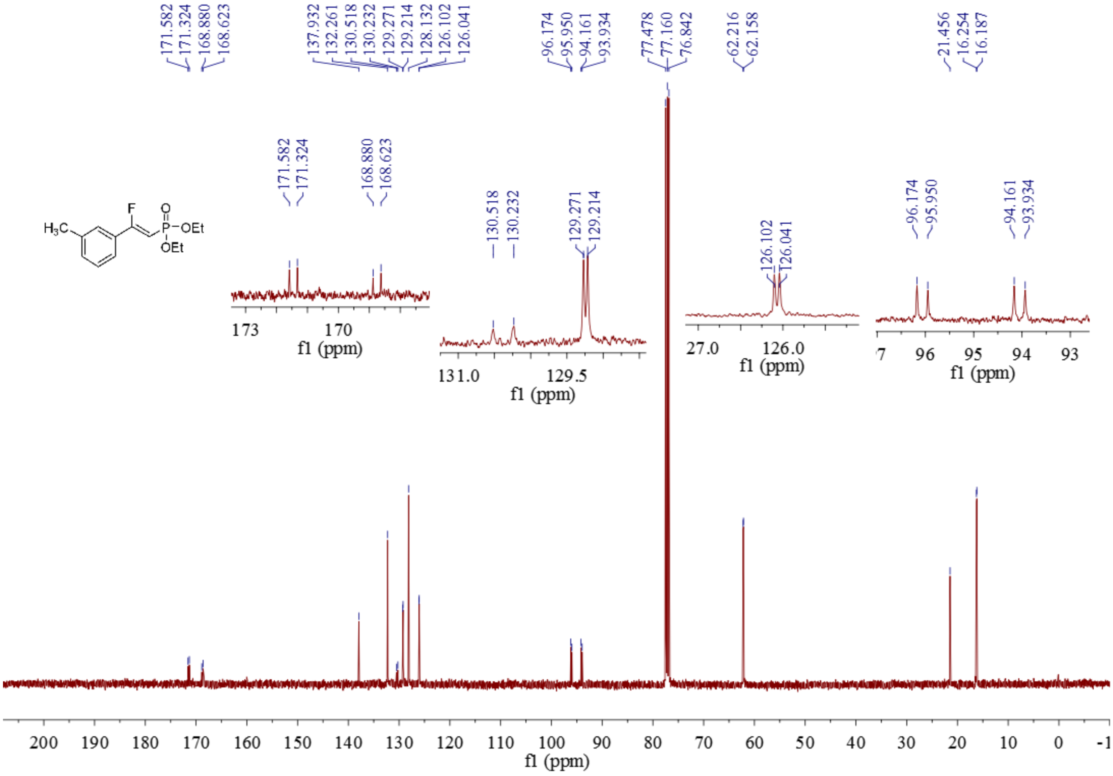

^{19}F NMR

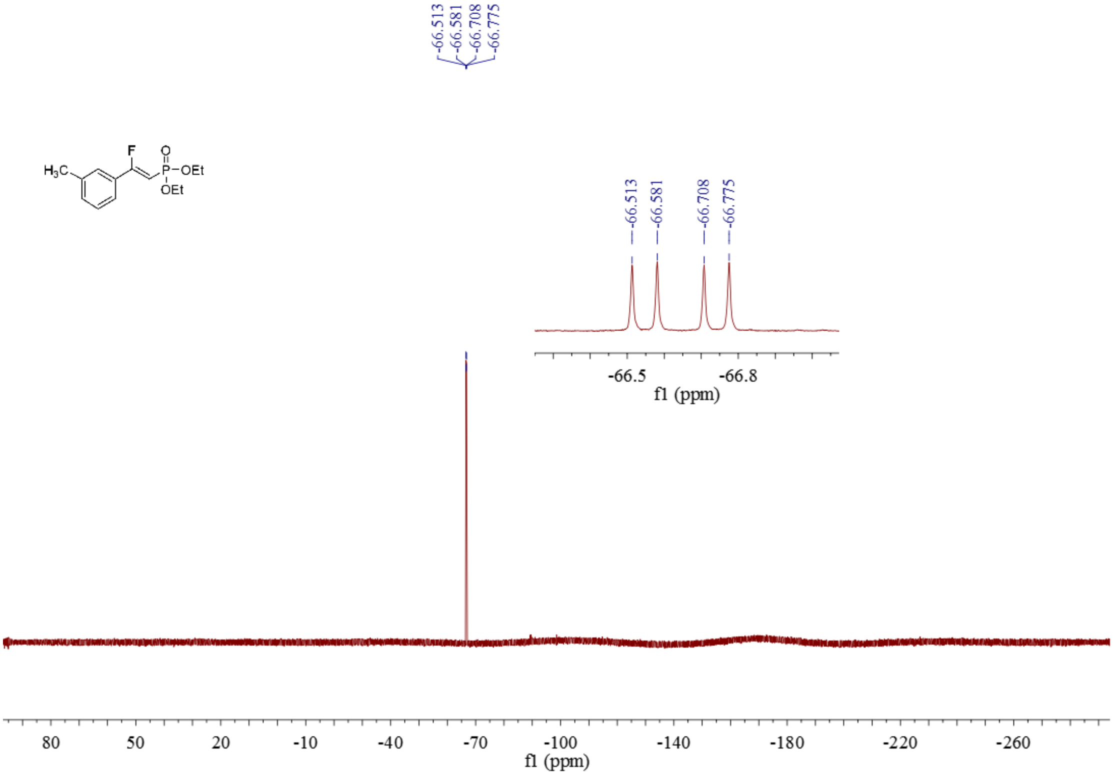

^{31}P NMR

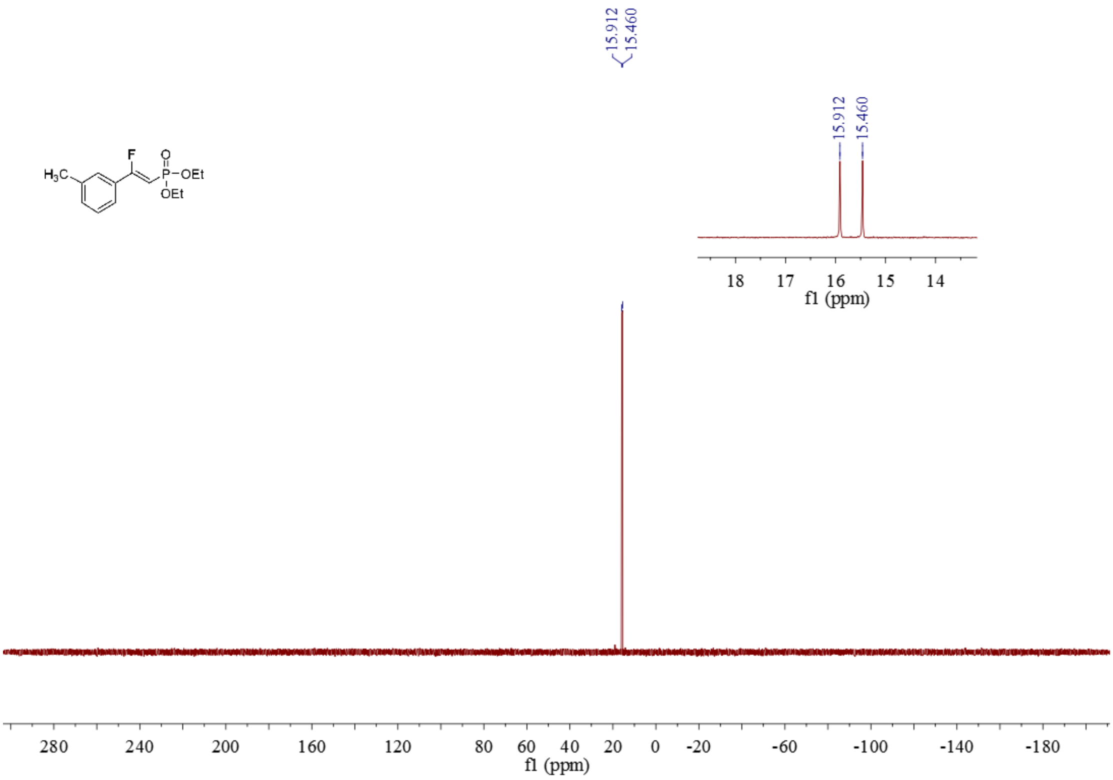

Diethyl (*Z*)-(2-(4-(tert-butyl)phenyl)-2-fluorovinyl)phosphonate (2d)

^{1}H NMR

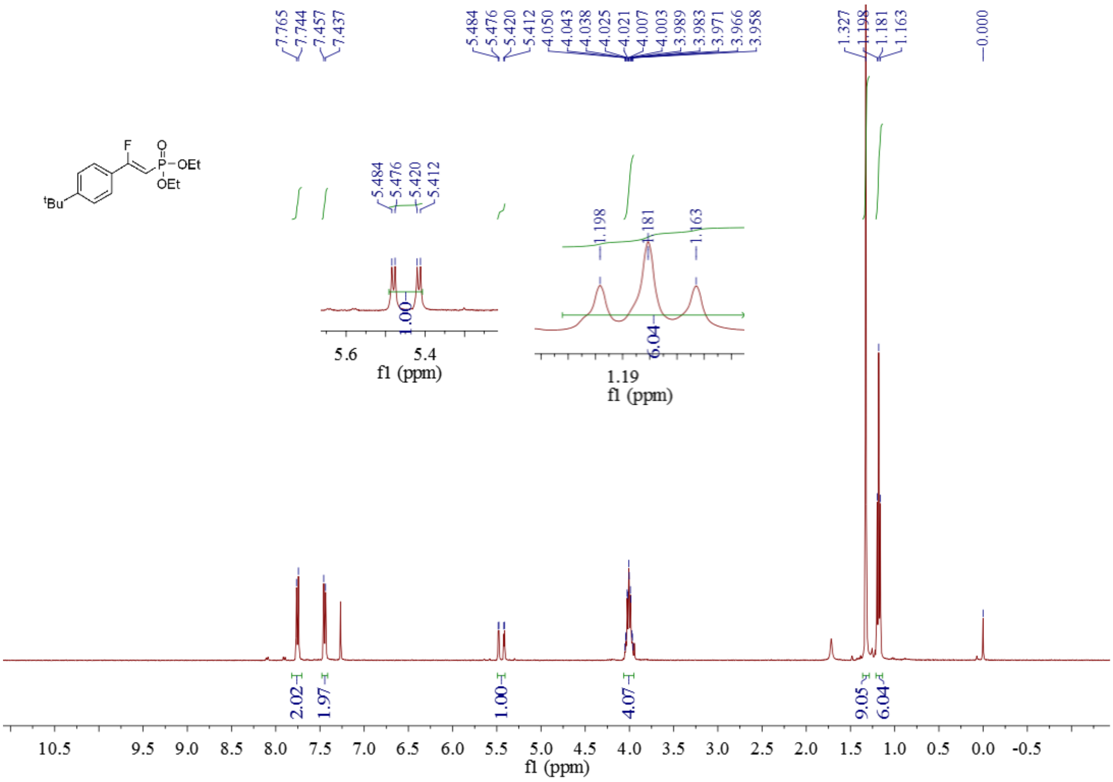

^{13}C NMR

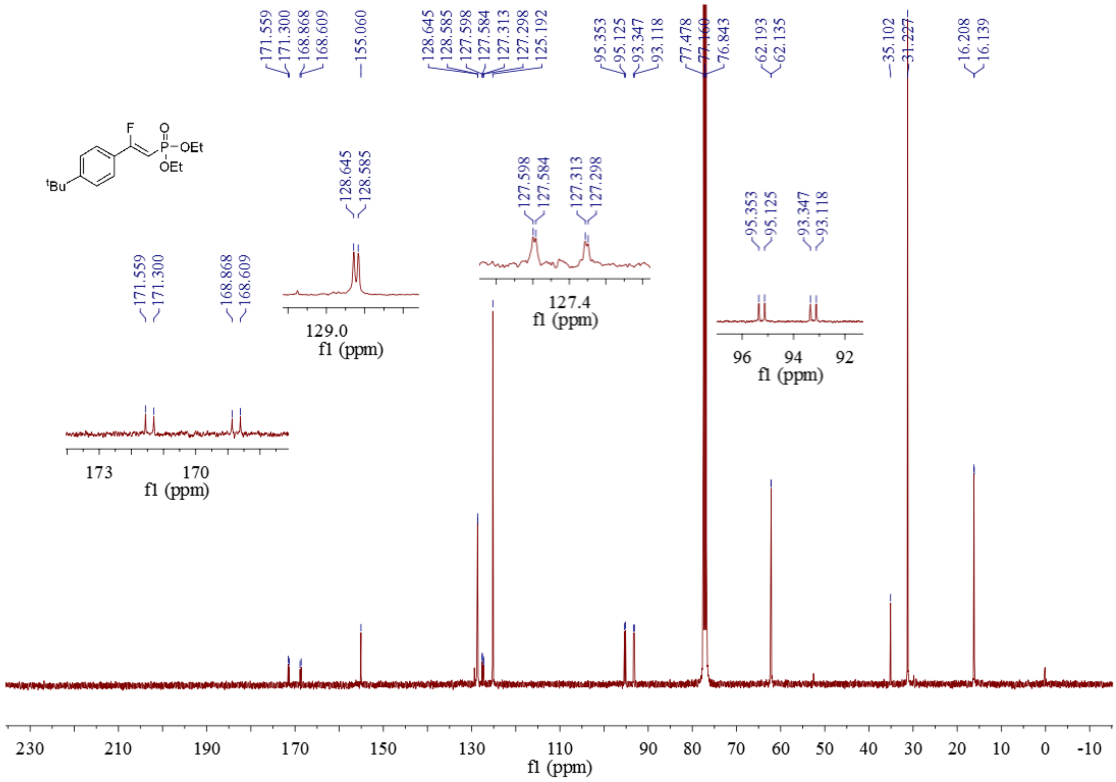

^{19}F NMR

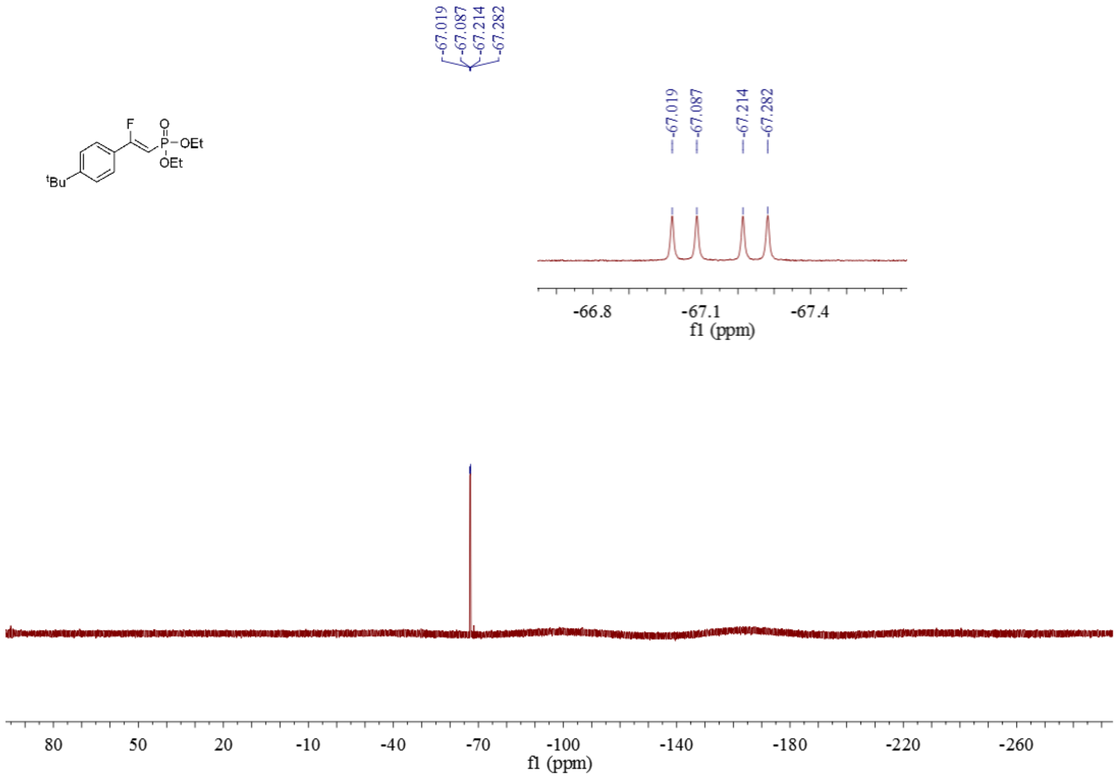

^{31}P NMR

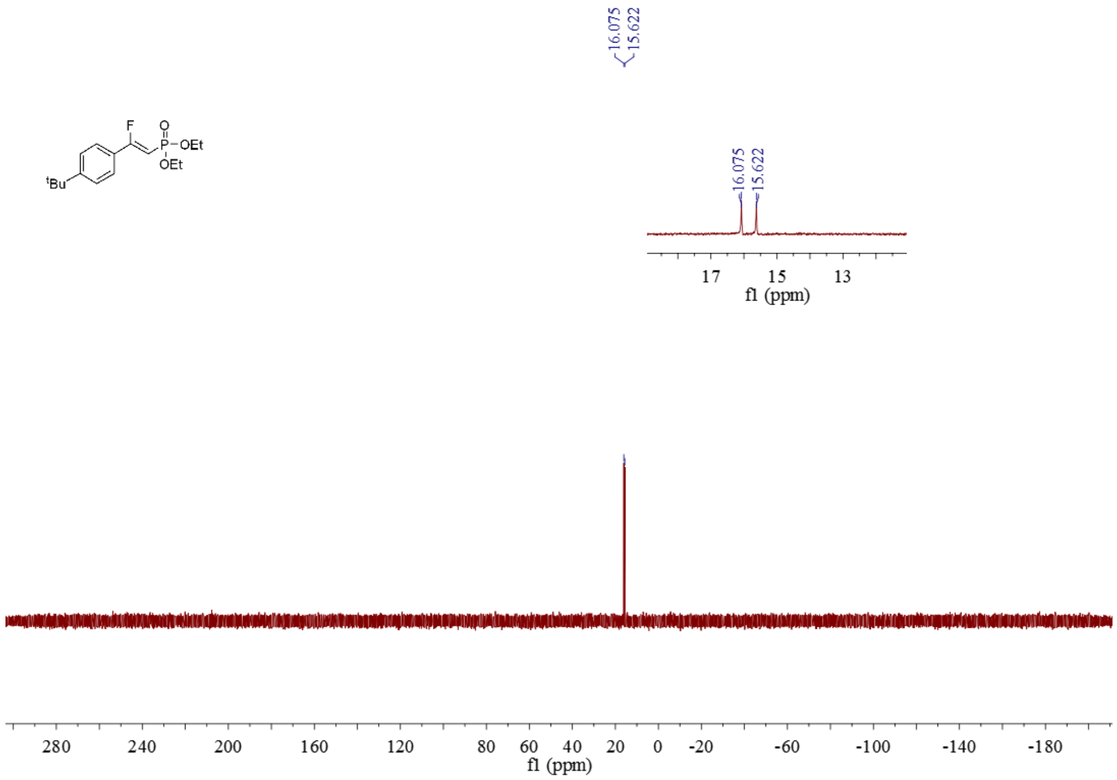

Diethyl (*Z*)-(2-fluoro-2-(4-methoxyphenyl)vinyl)phosphonate (2e)

^{1}H NMR

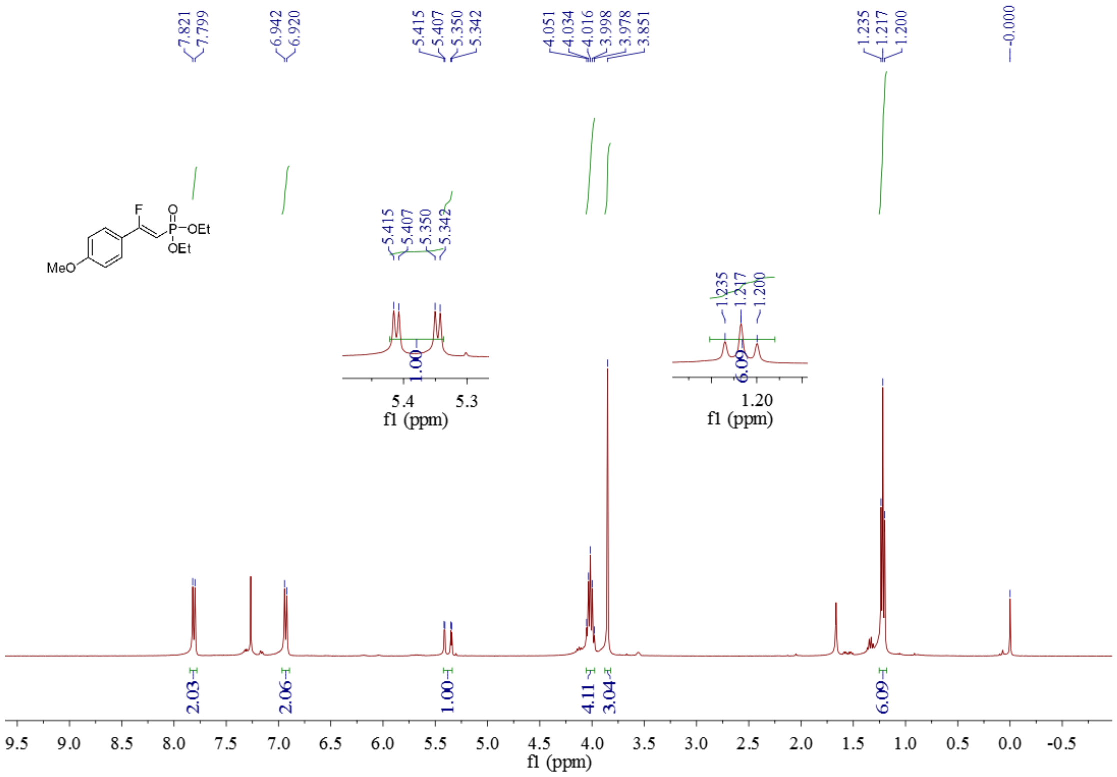

^{13}C NMR

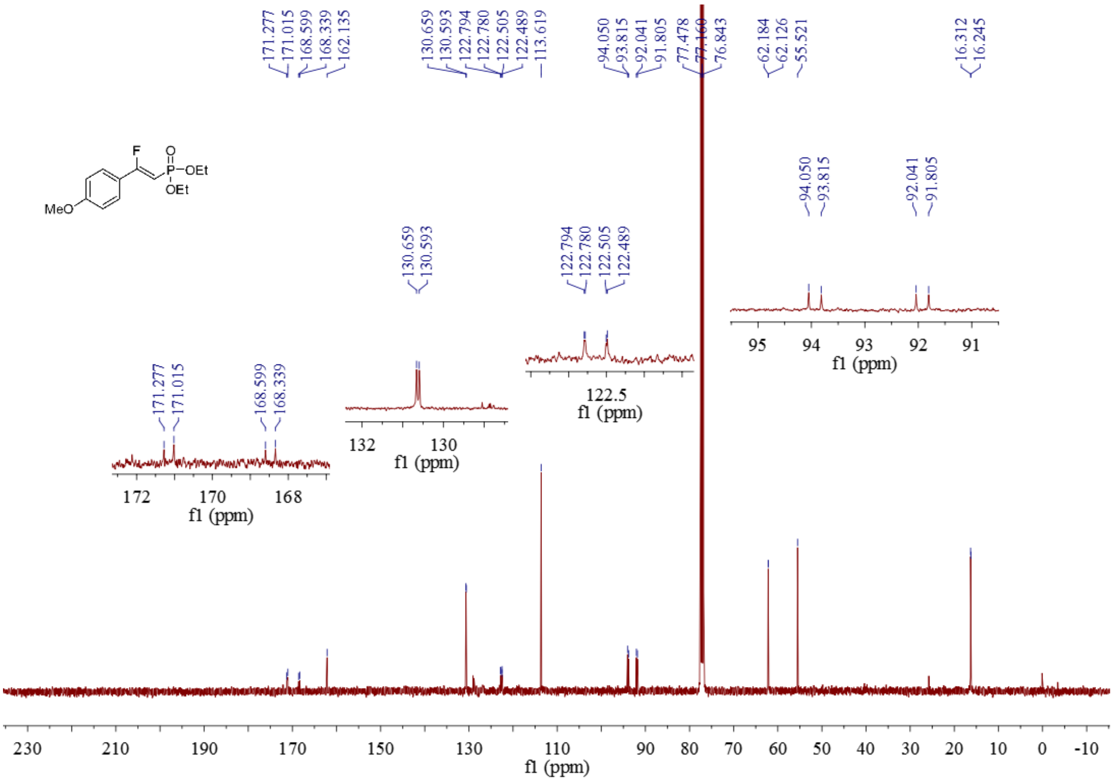

^{19}F NMR

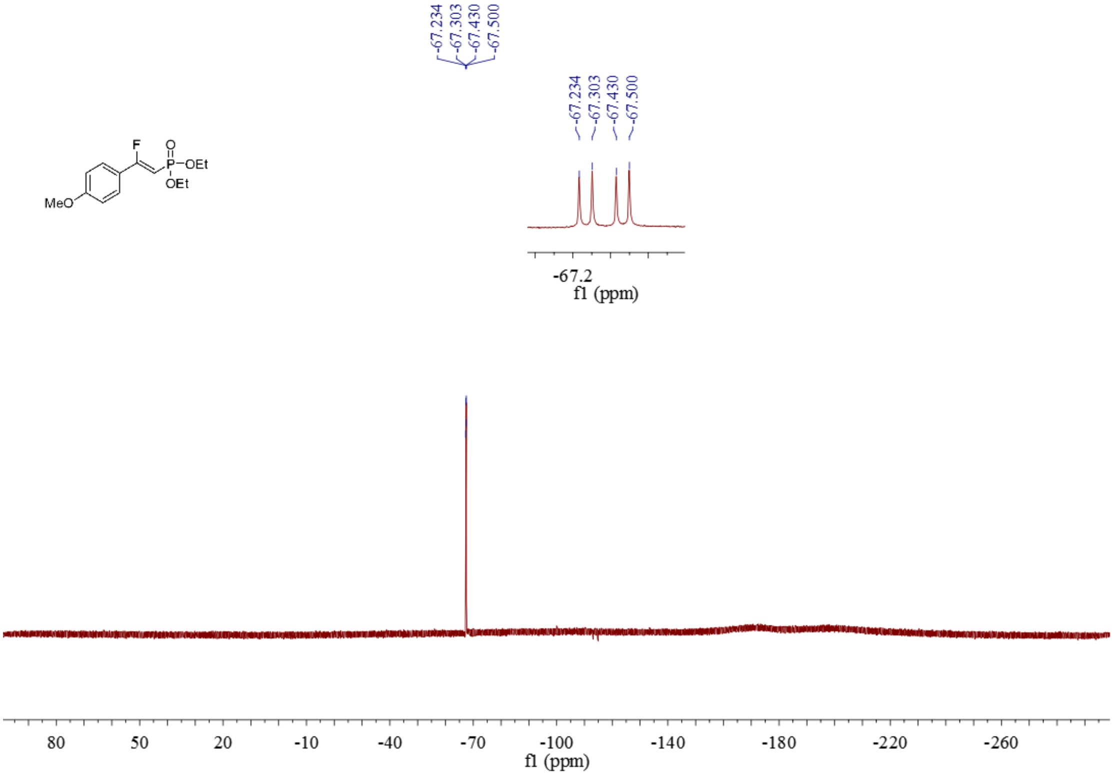

^{31}P NMR

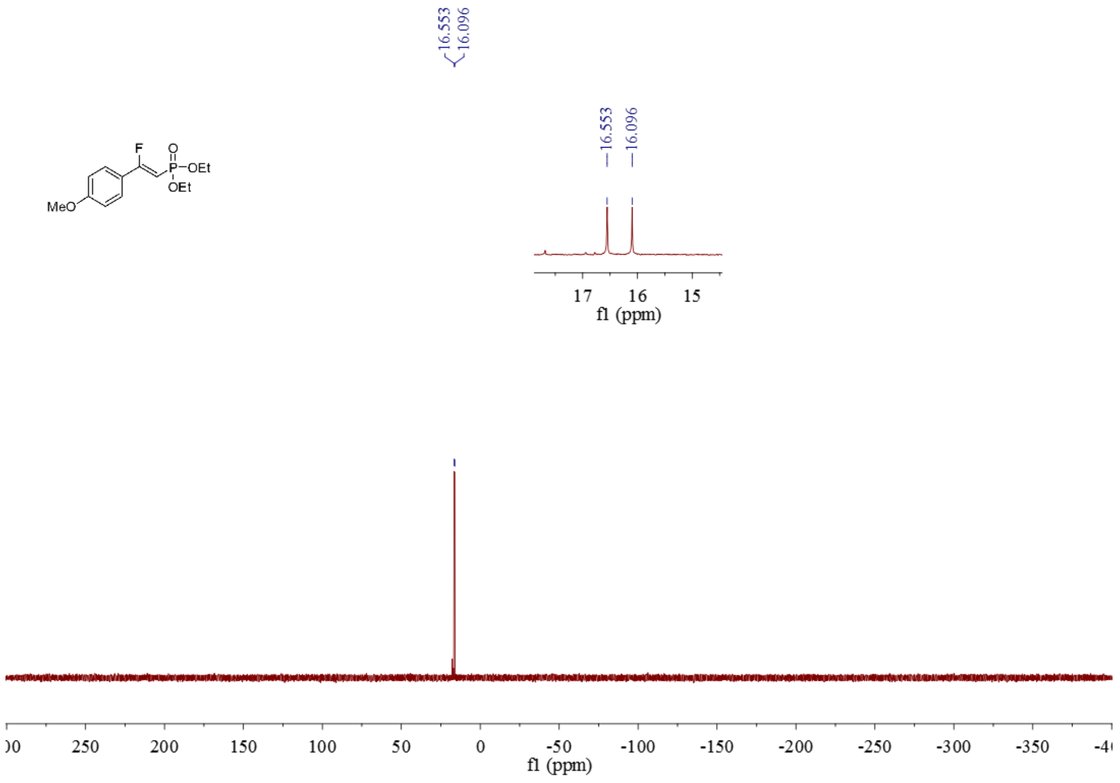

Diethyl (*Z*)-(2-([1,1'-biphenyl]-4-yl)-2-fluorovinyl)phosphonate (2f)

^{1}H NMR

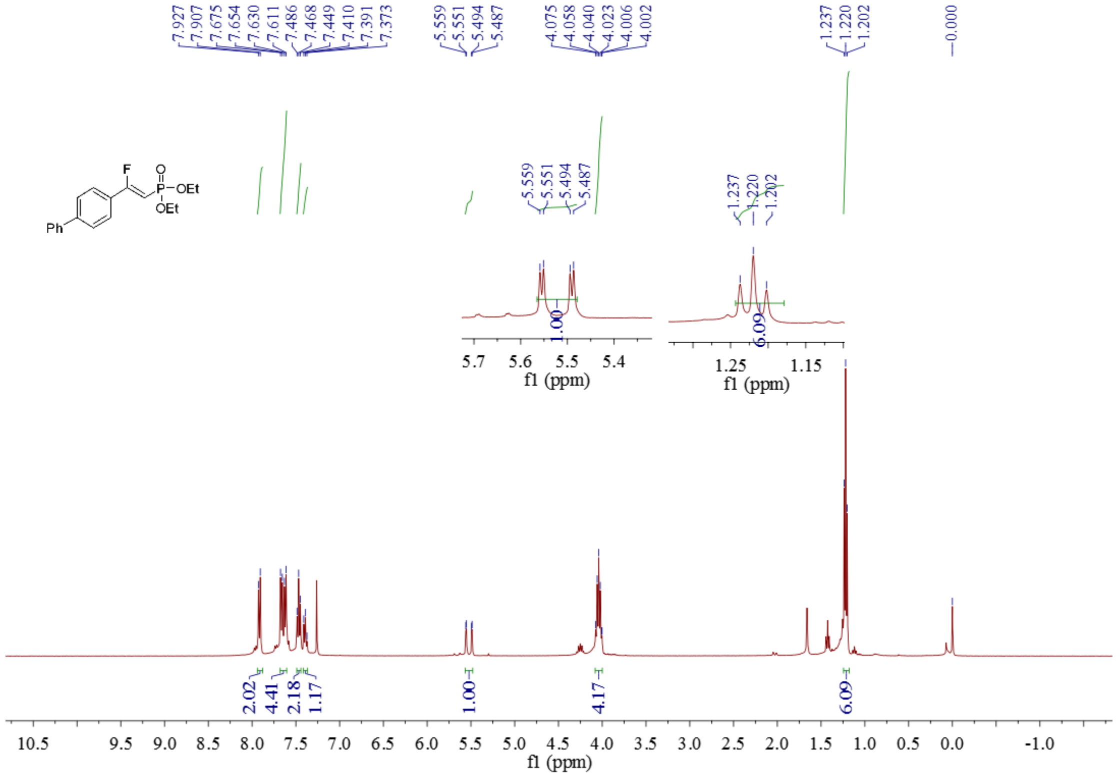

^{13}C NMR

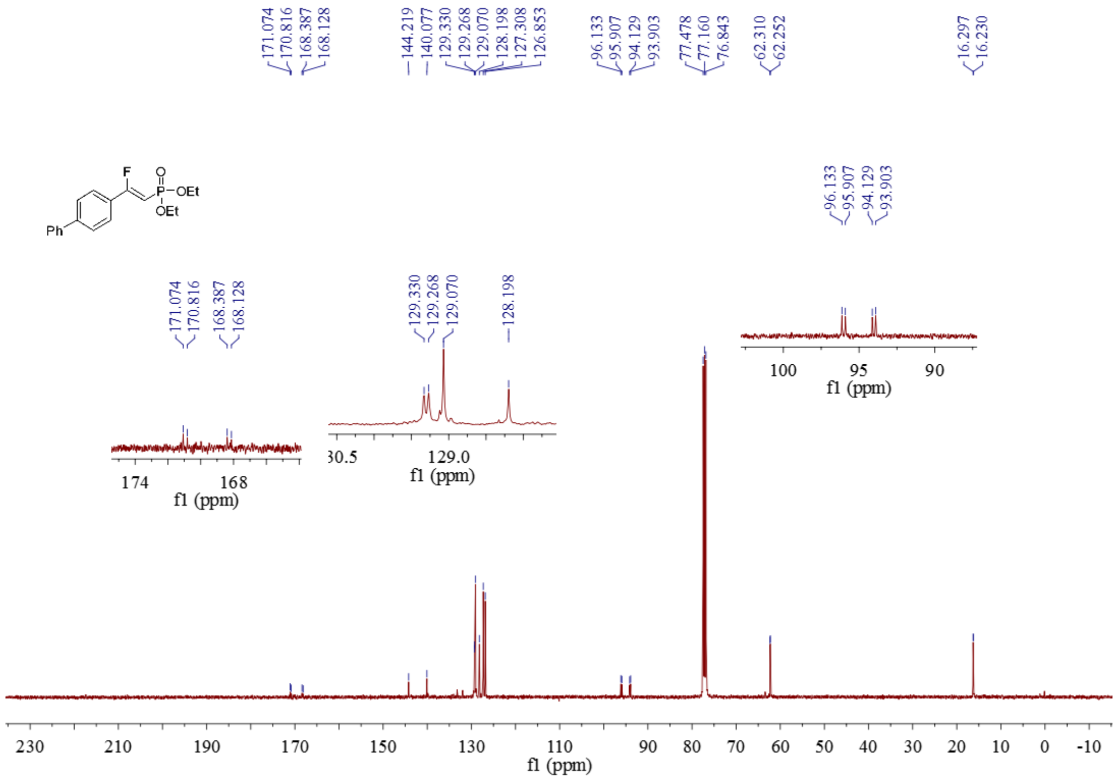

^{19}F NMR

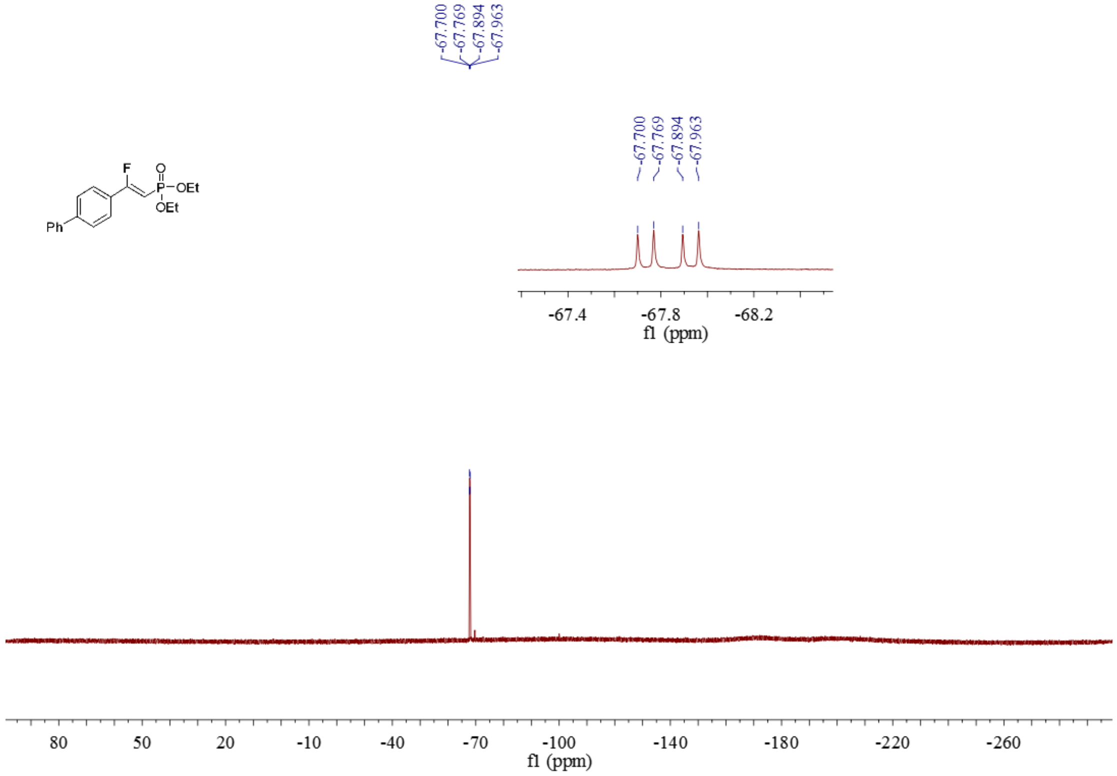

^{31}P NMR

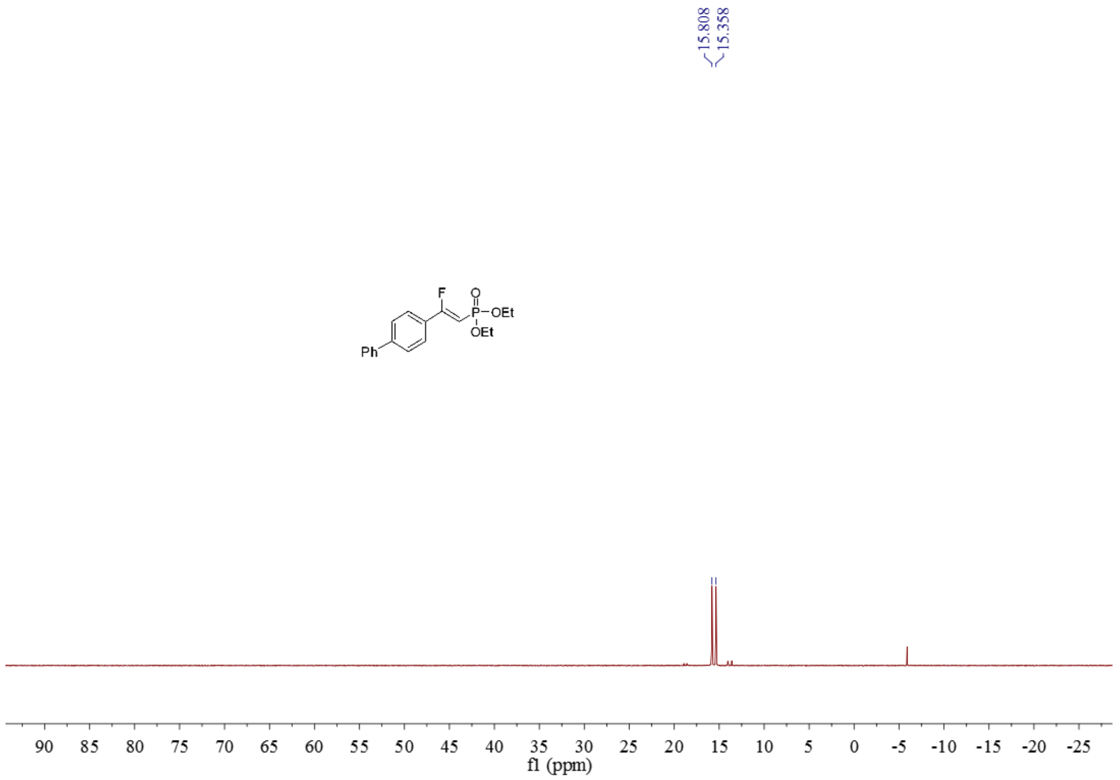

Diethyl (*Z*)-(2-fluoro-2-(4-fluorophenyl)vinyl)phosphonate (2g)

^{1}H NMR

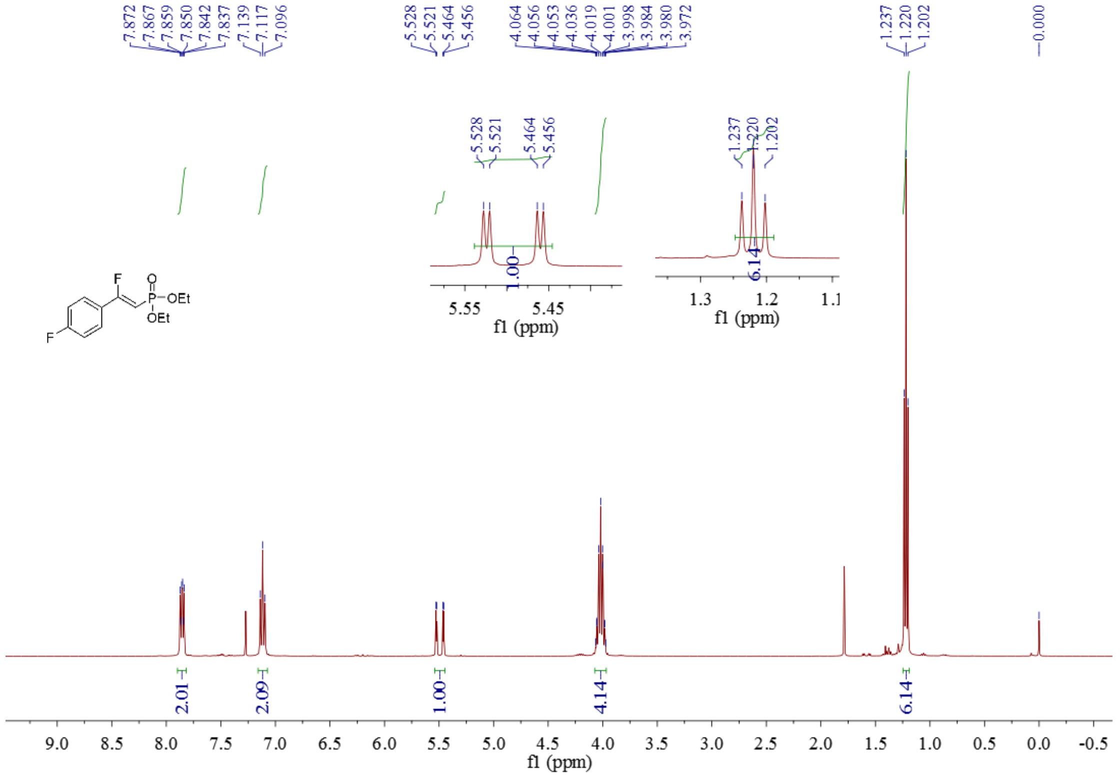

^{13}C NMR

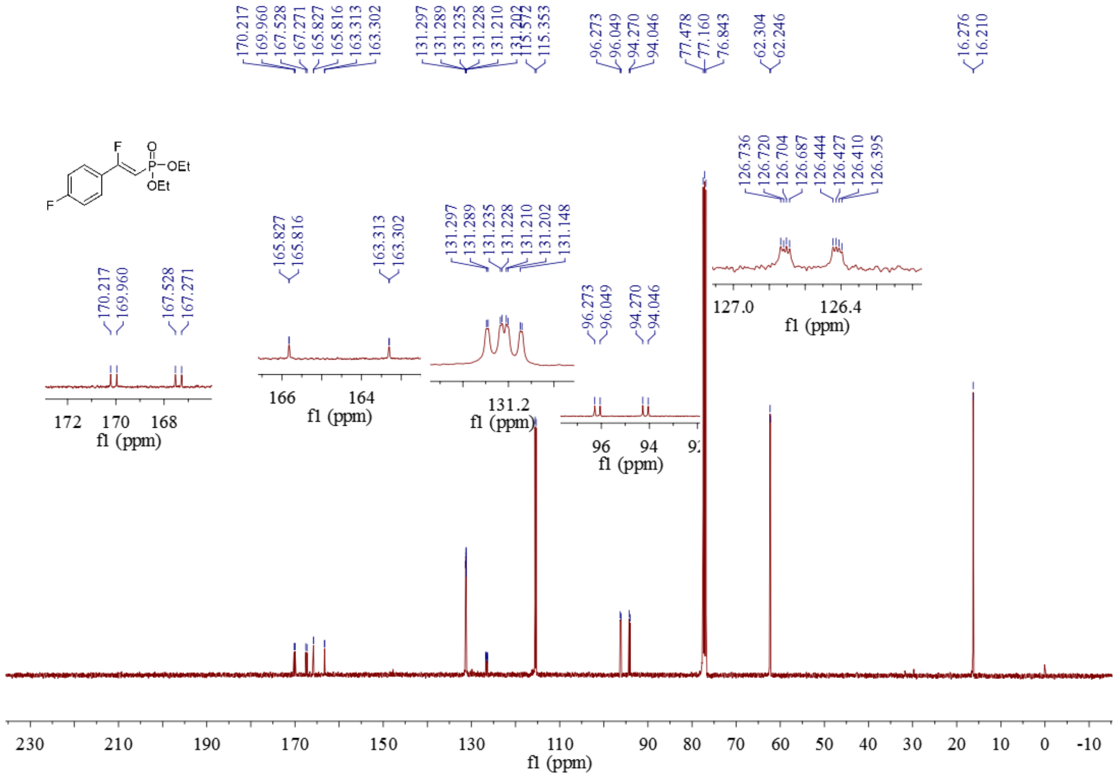

^{19}F NMR

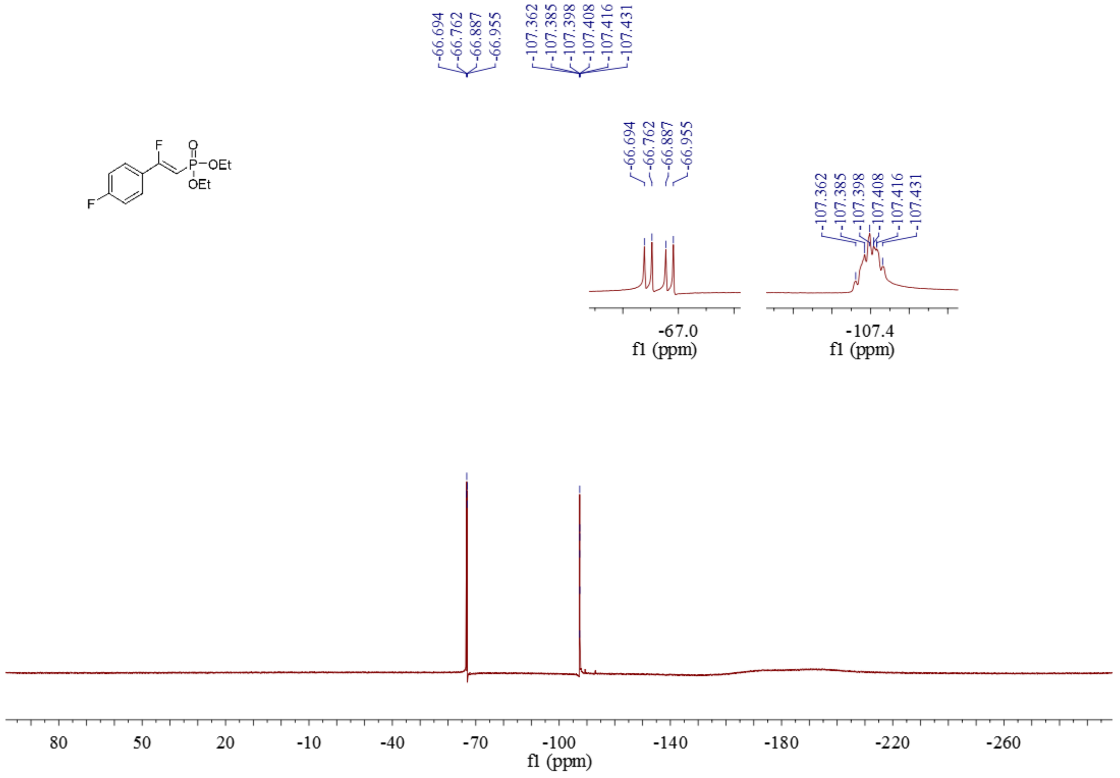

^{31}P NMR

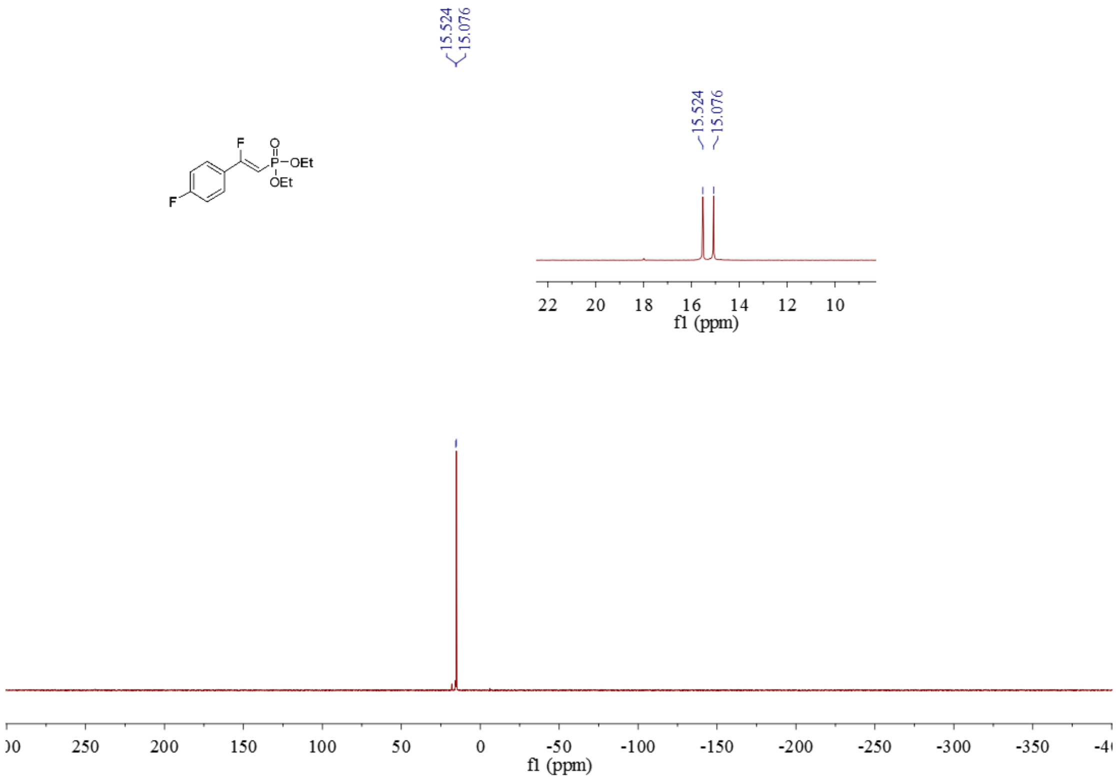

Diethyl (*Z*)-(2-fluoro-2-(3-fluorophenyl)vinyl)phosphonate (2h)

^{1}H NMR

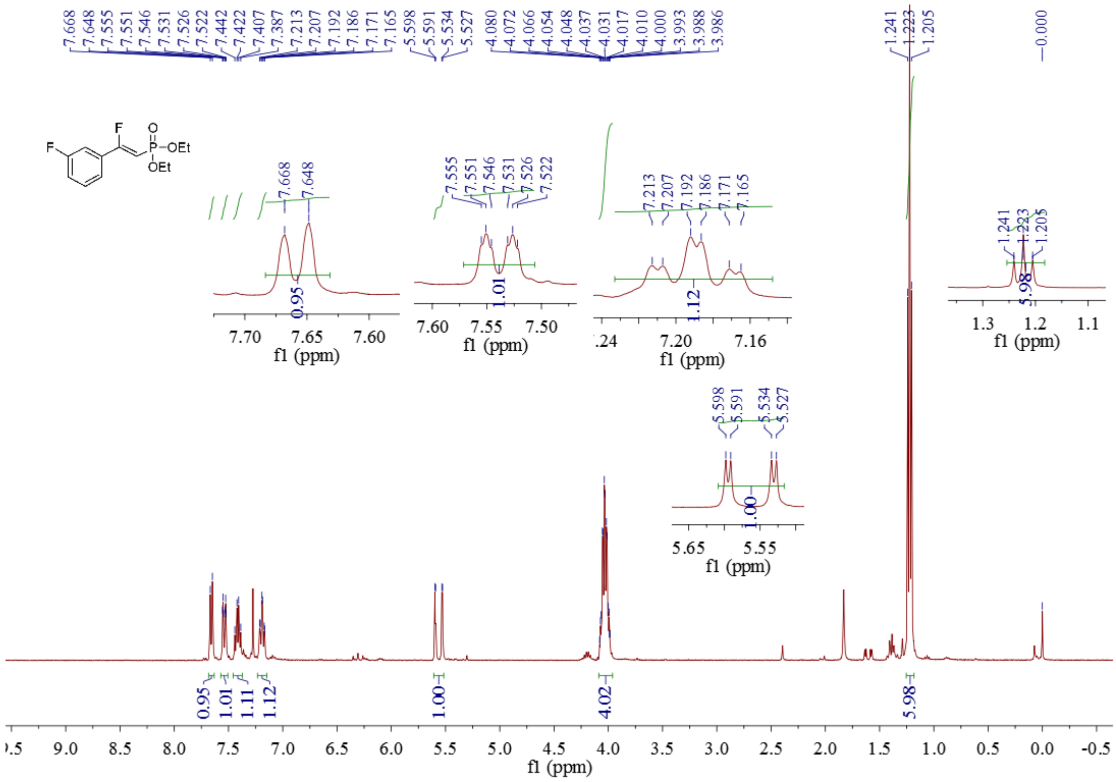

^{13}C NMR

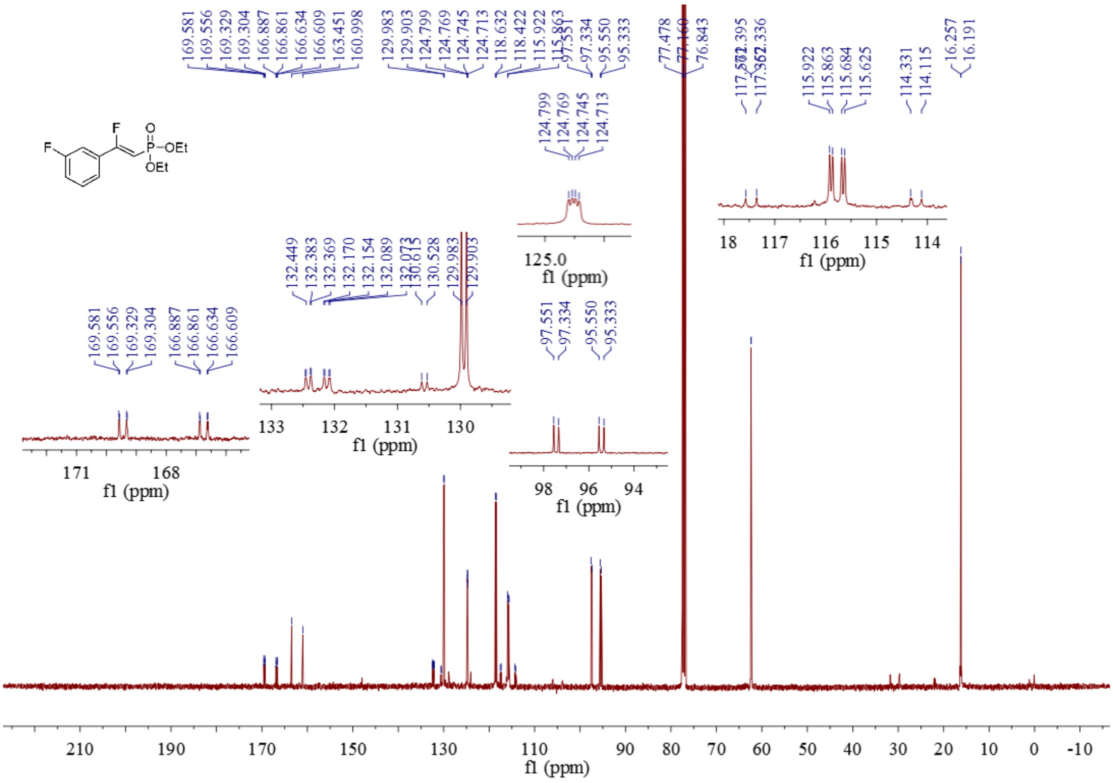

^{19}F NMR

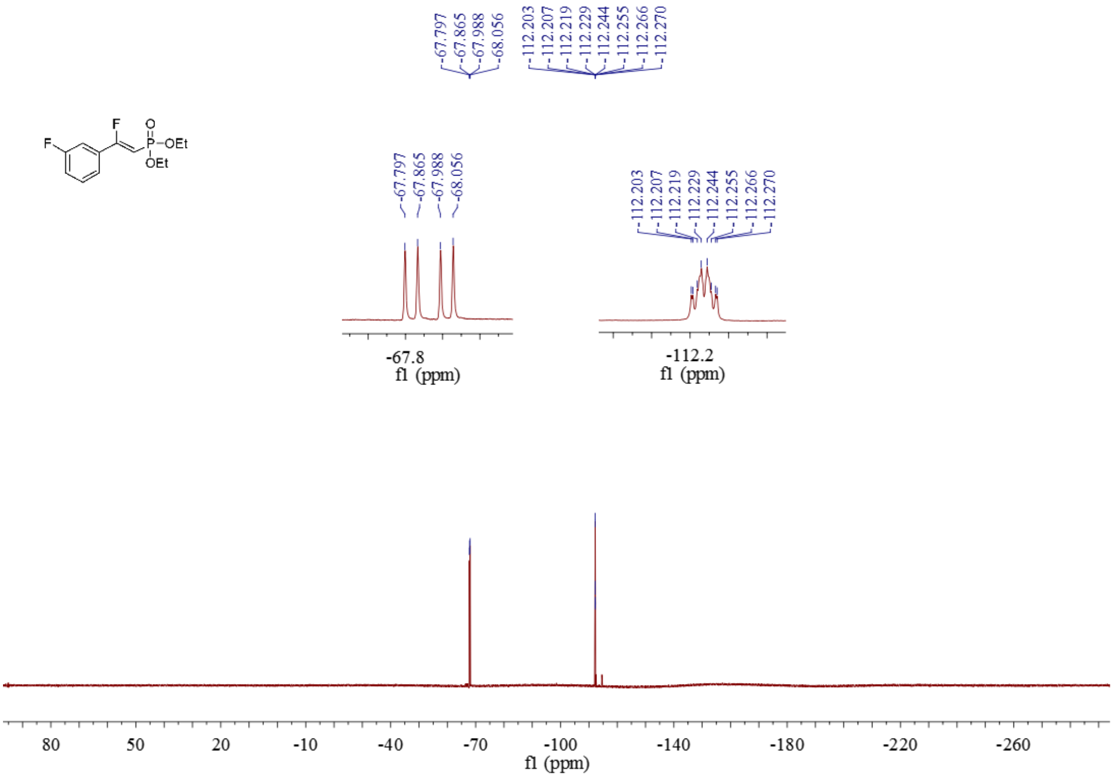

^{31}P NMR

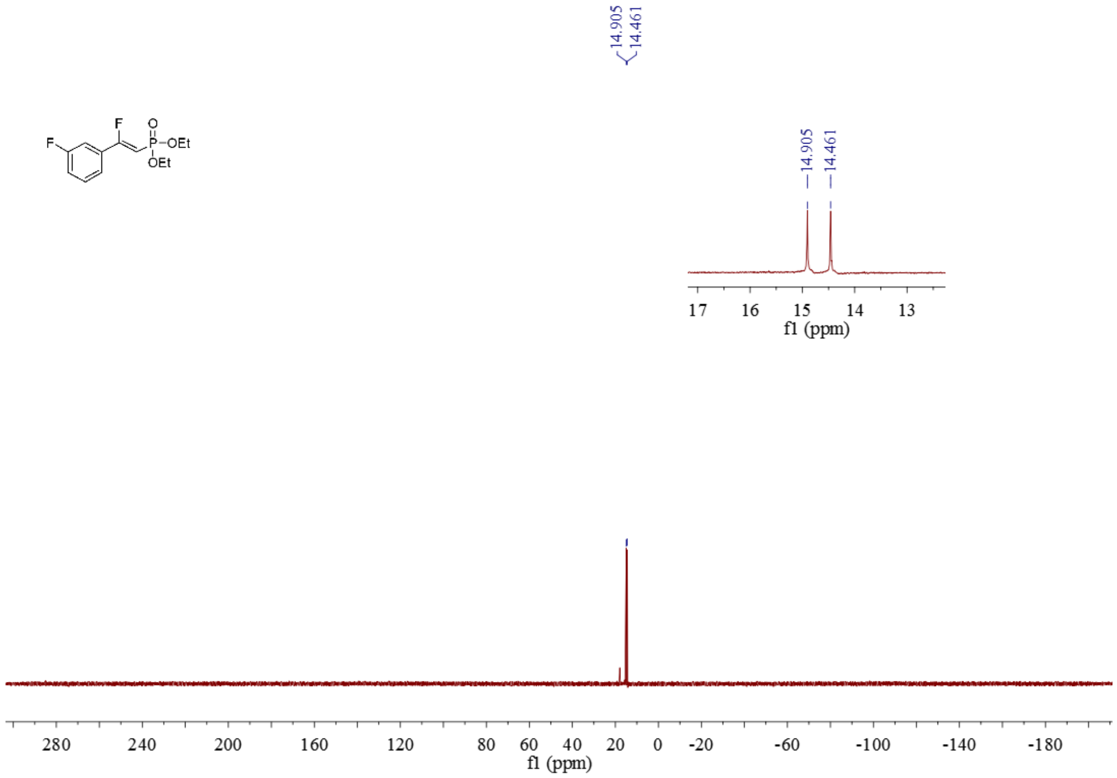

Diethyl (*Z*)-(2-(4-chlorophenyl)-2-fluorovinyl)phosphonate (2i)

^{1}H NMR

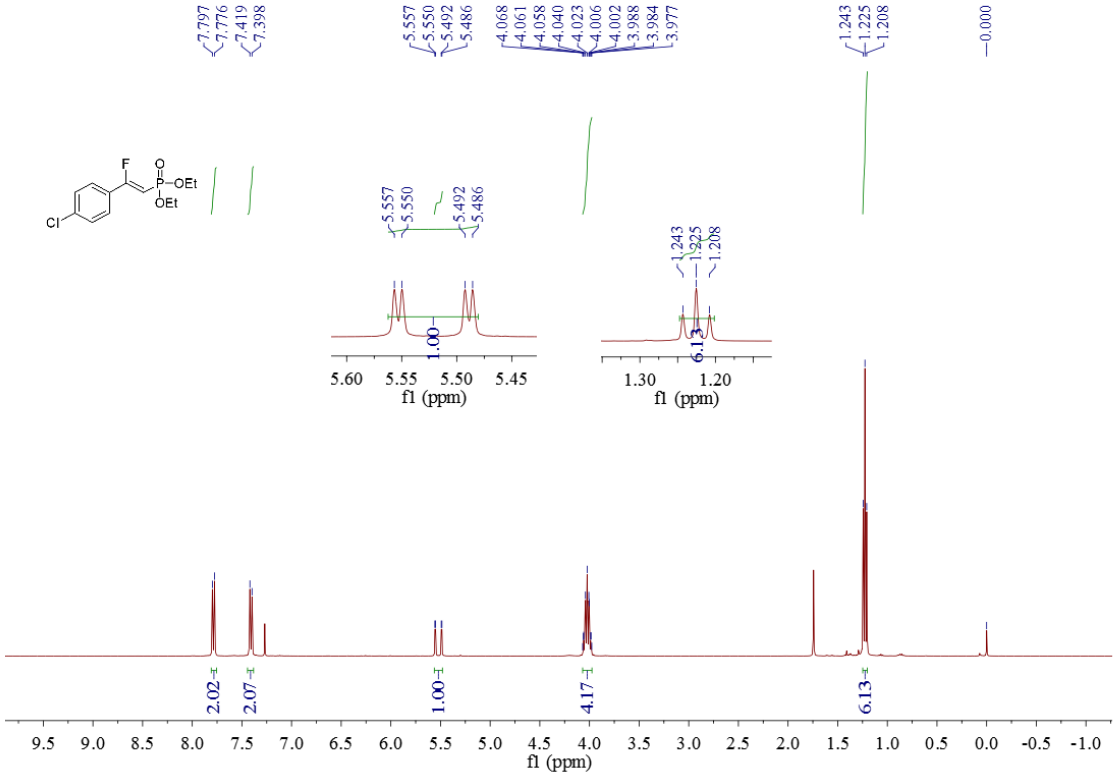

^{13}C NMR

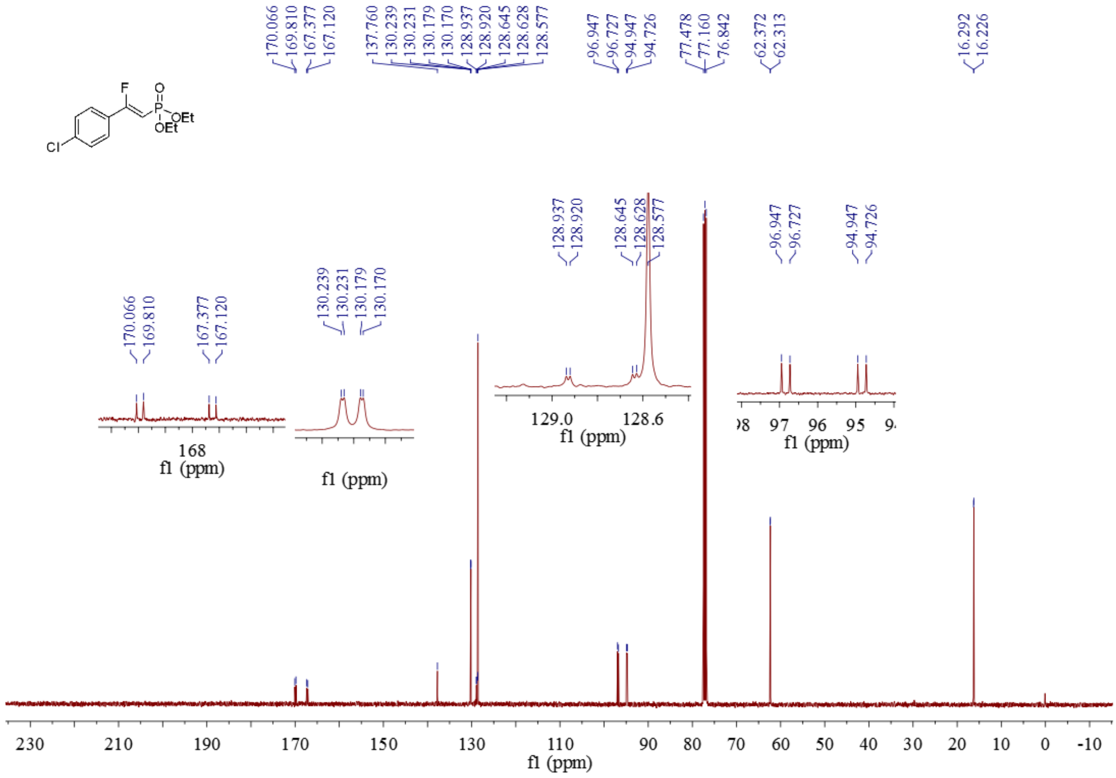

^{19}F NMR

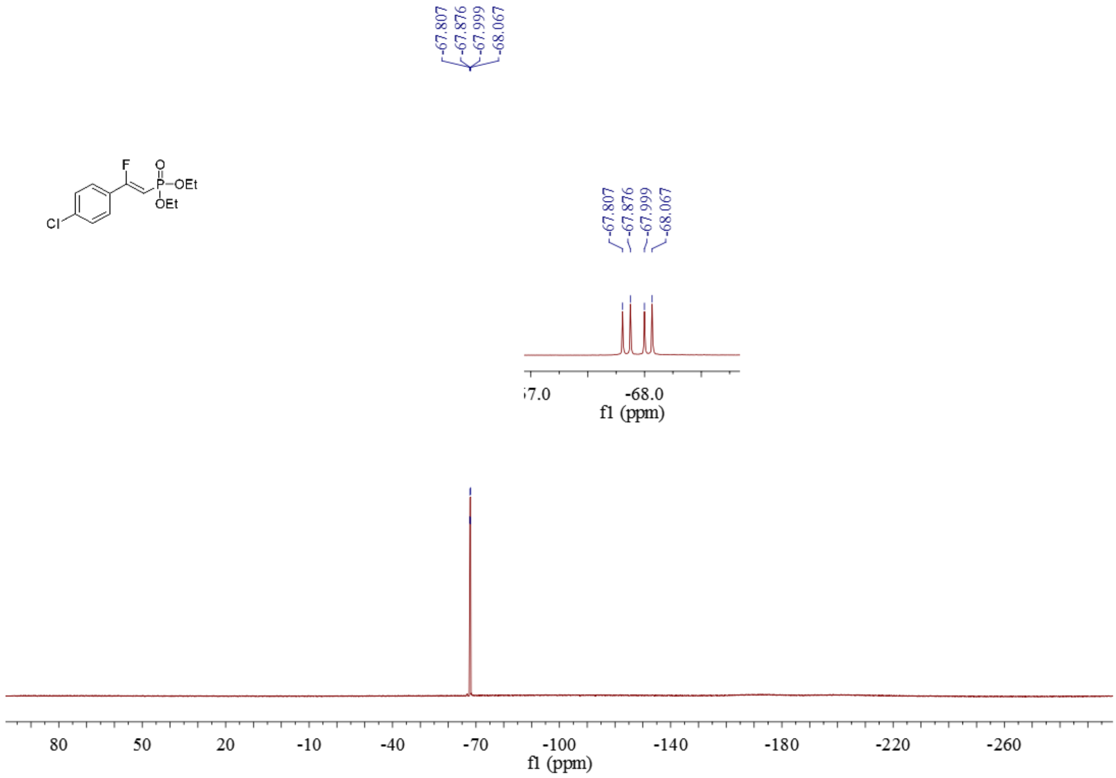

^{31}P NMR

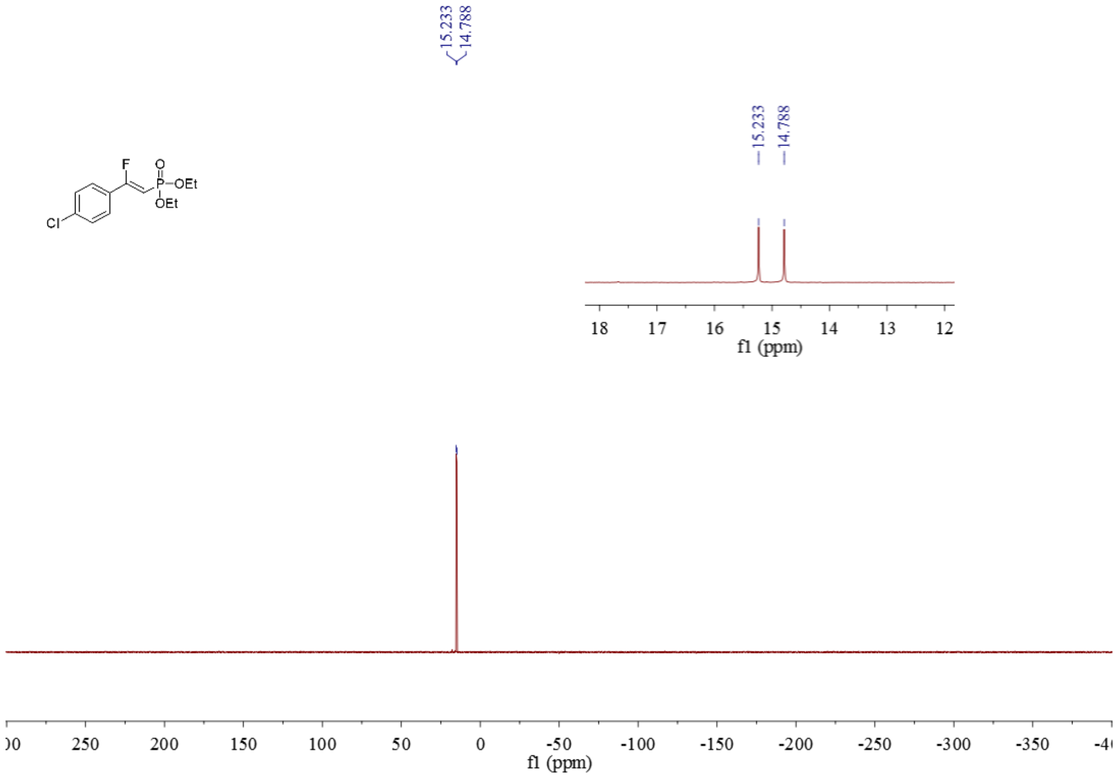

Diethyl (*Z*)-(2-(3-chlorophenyl)-2-fluorovinyl)phosphonate (2j)

^{1}H NMR

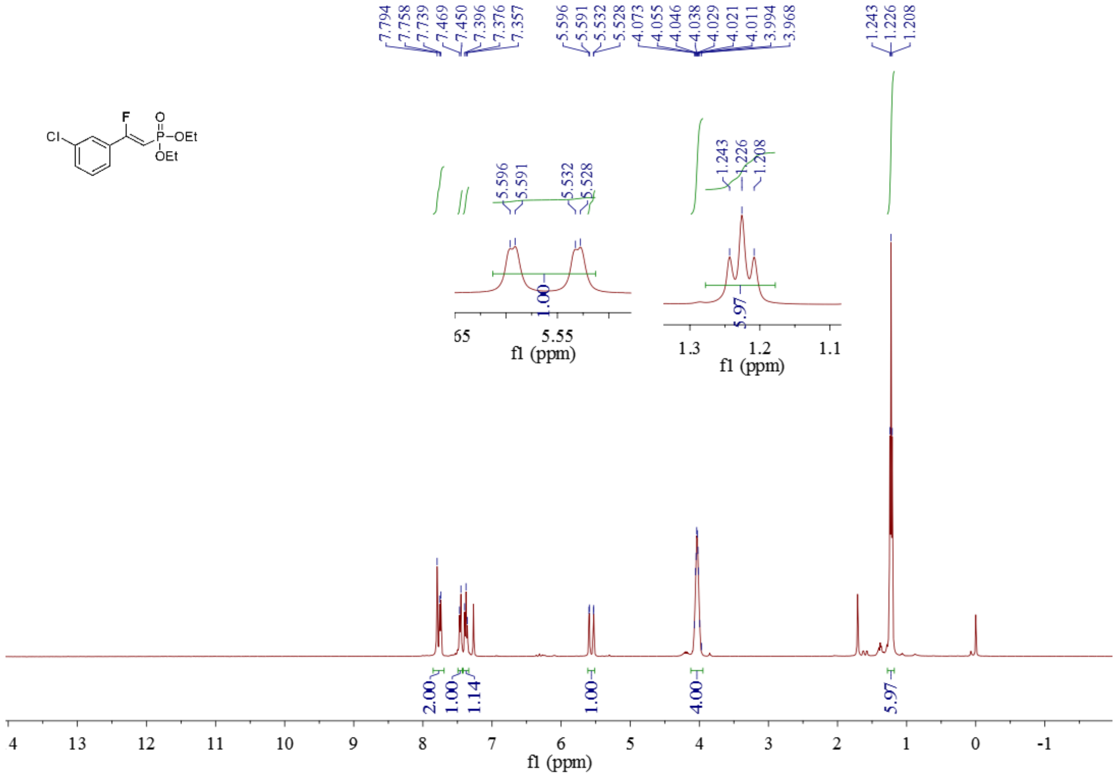

^{13}C NMR

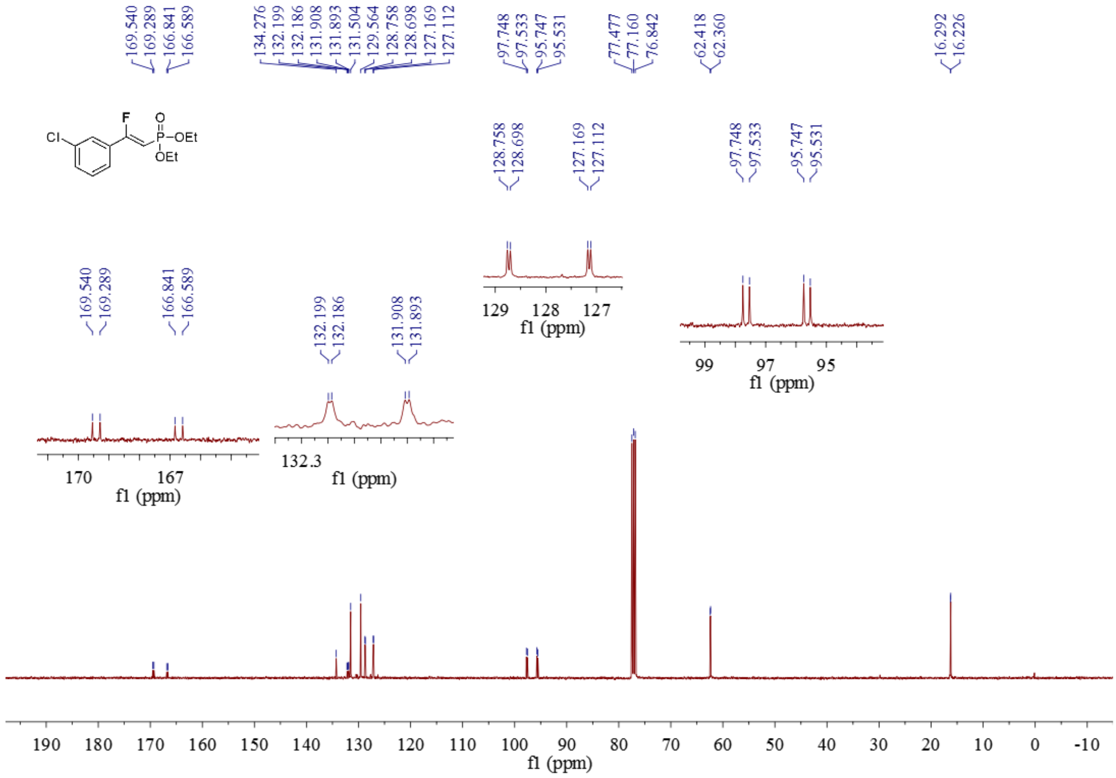

^{19}F NMR

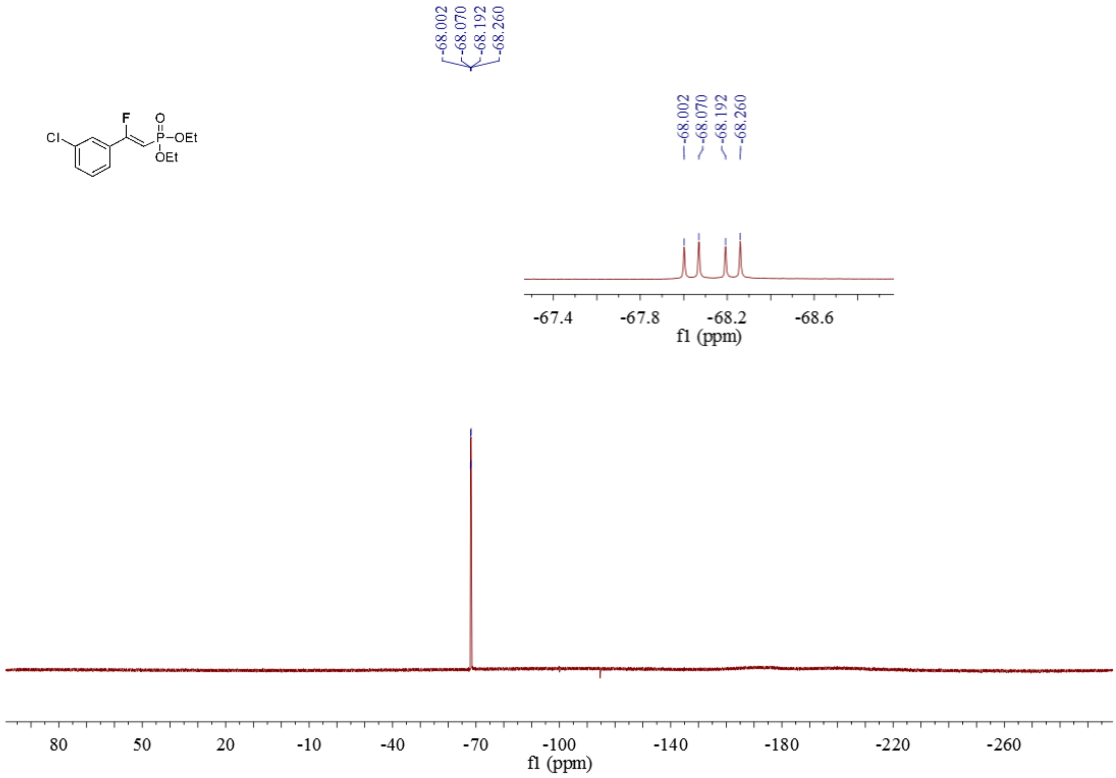

^{31}P NMR

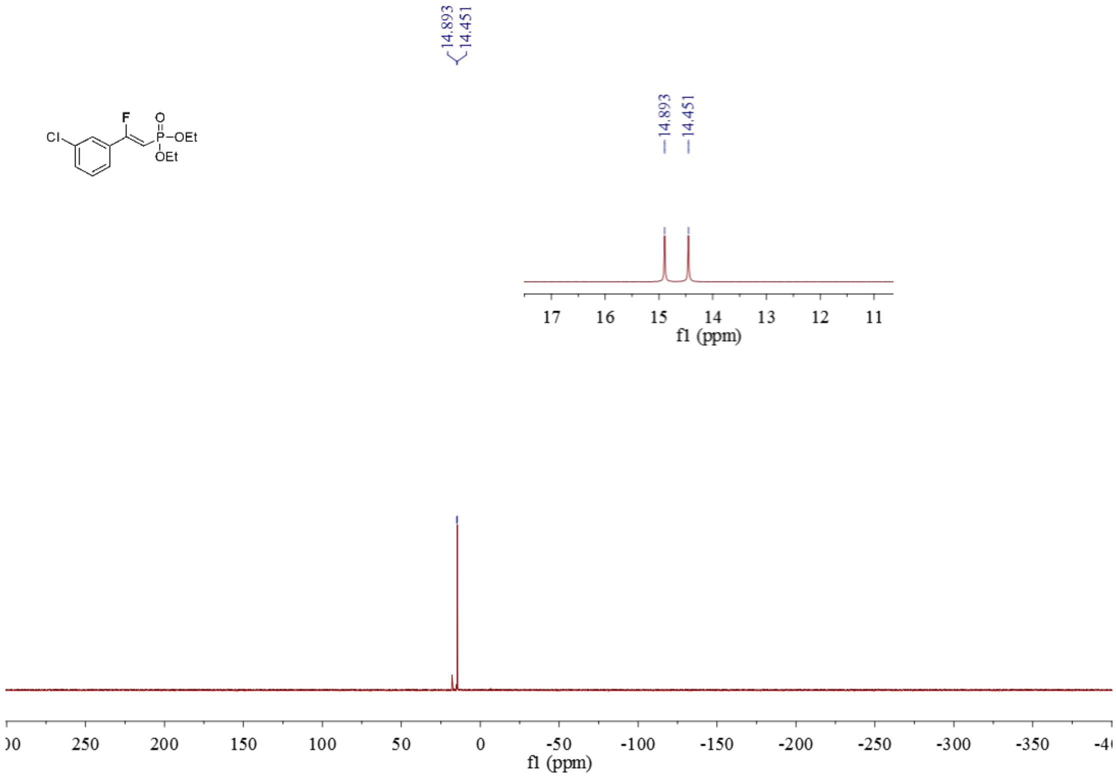

Diethyl (*Z*)-(2-(2-chlorophenyl)-2-fluorovinyl)phosphonate (2k)

^{1}H NMR

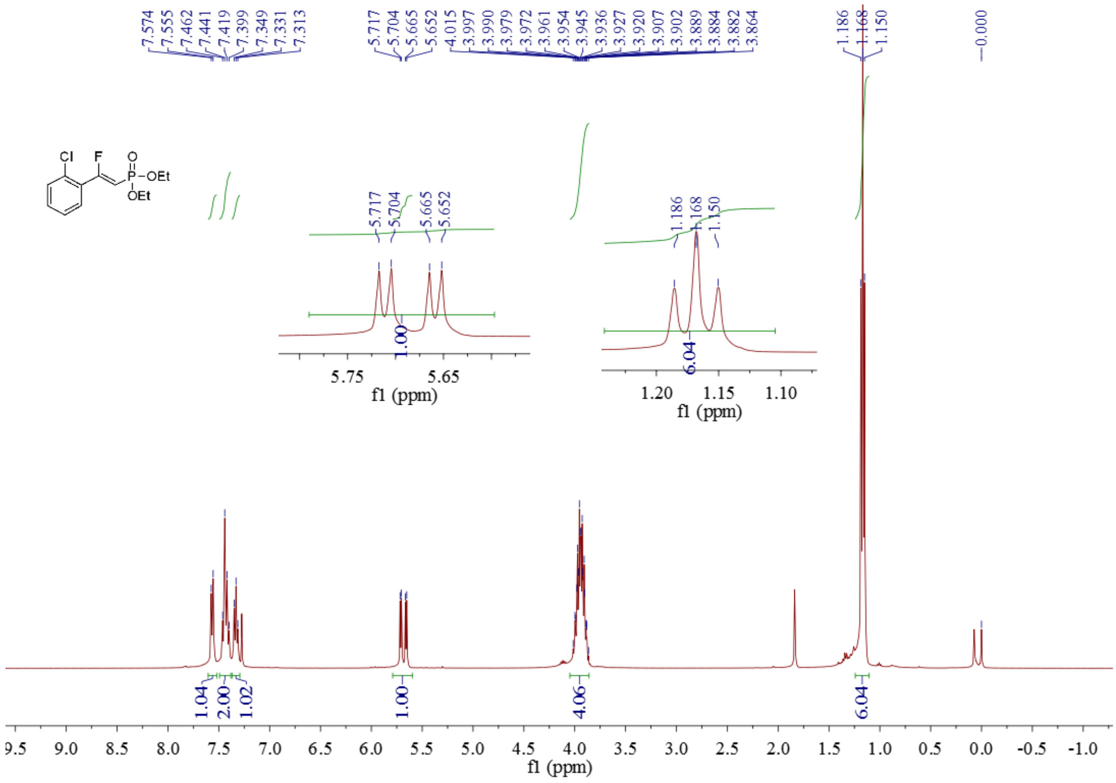

^{13}C NMR

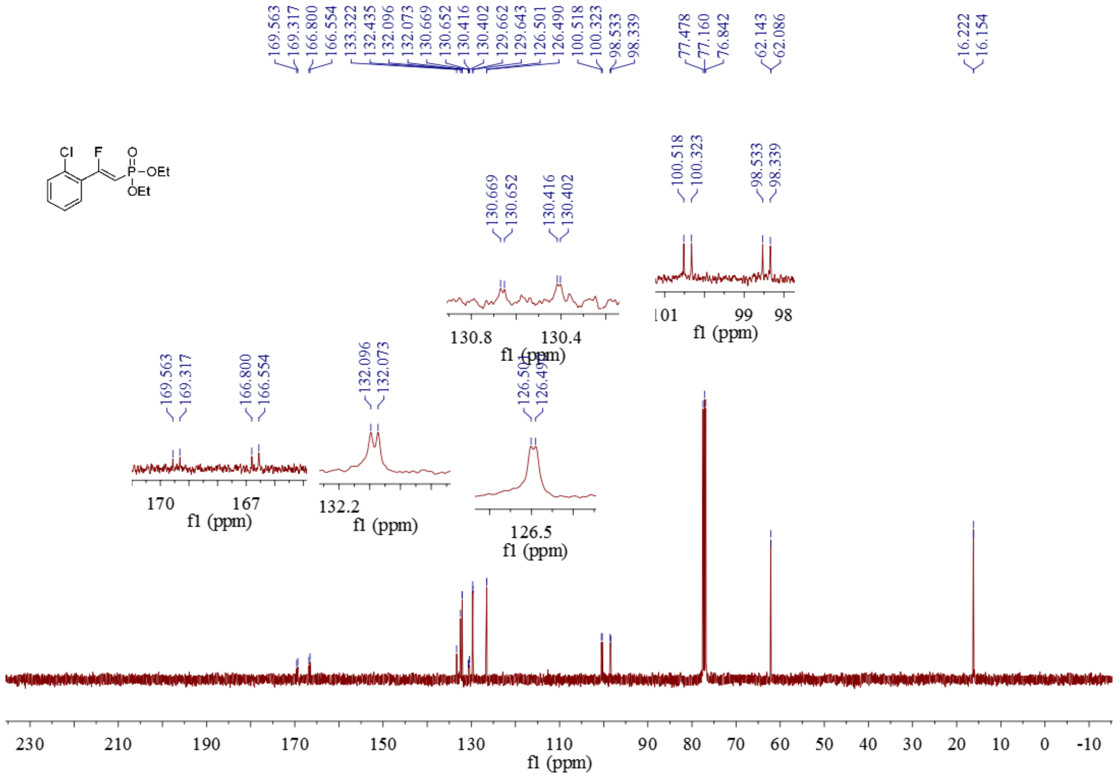

^{19}F NMR

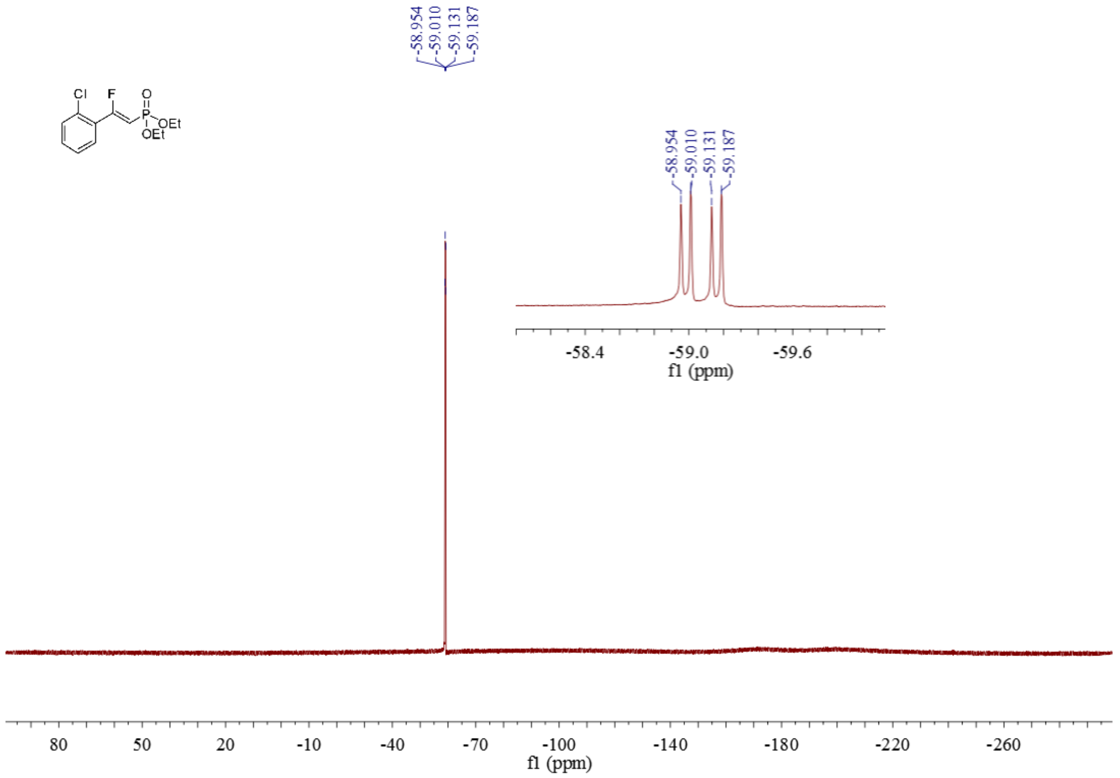

^{31}P NMR

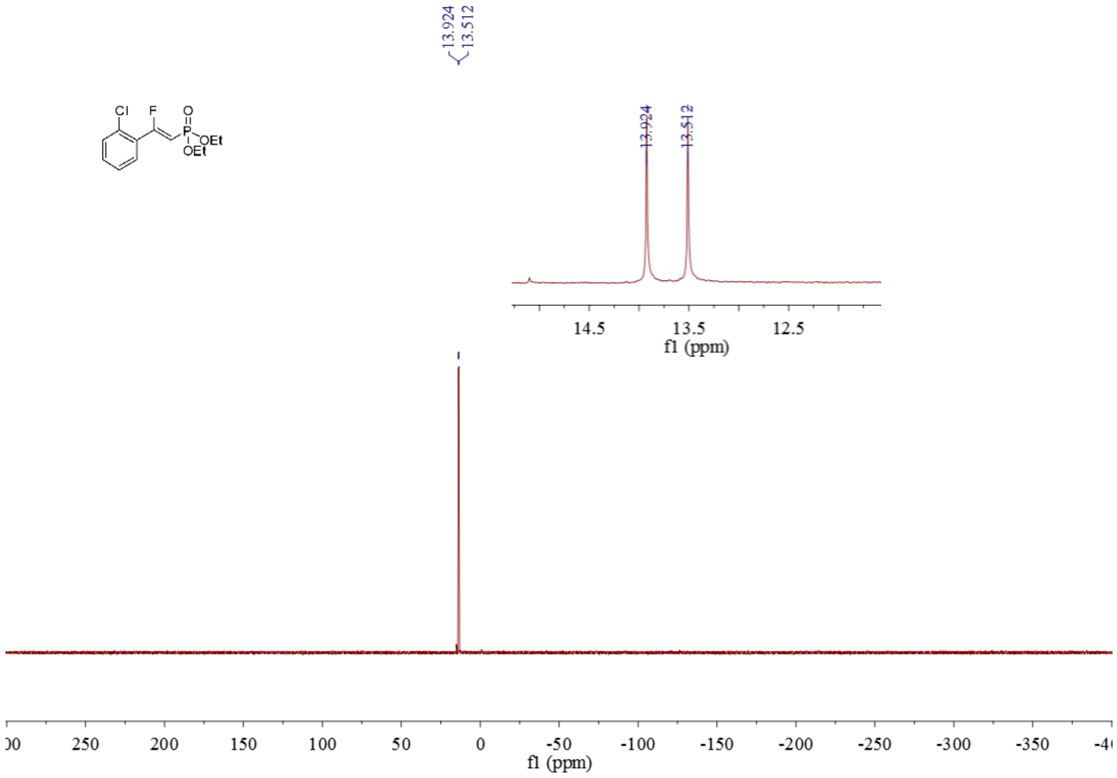

Diethyl (*Z*)-(2-(4-bromophenyl)-2-fluorovinyl)phosphonate (2l)

^{1}H NMR

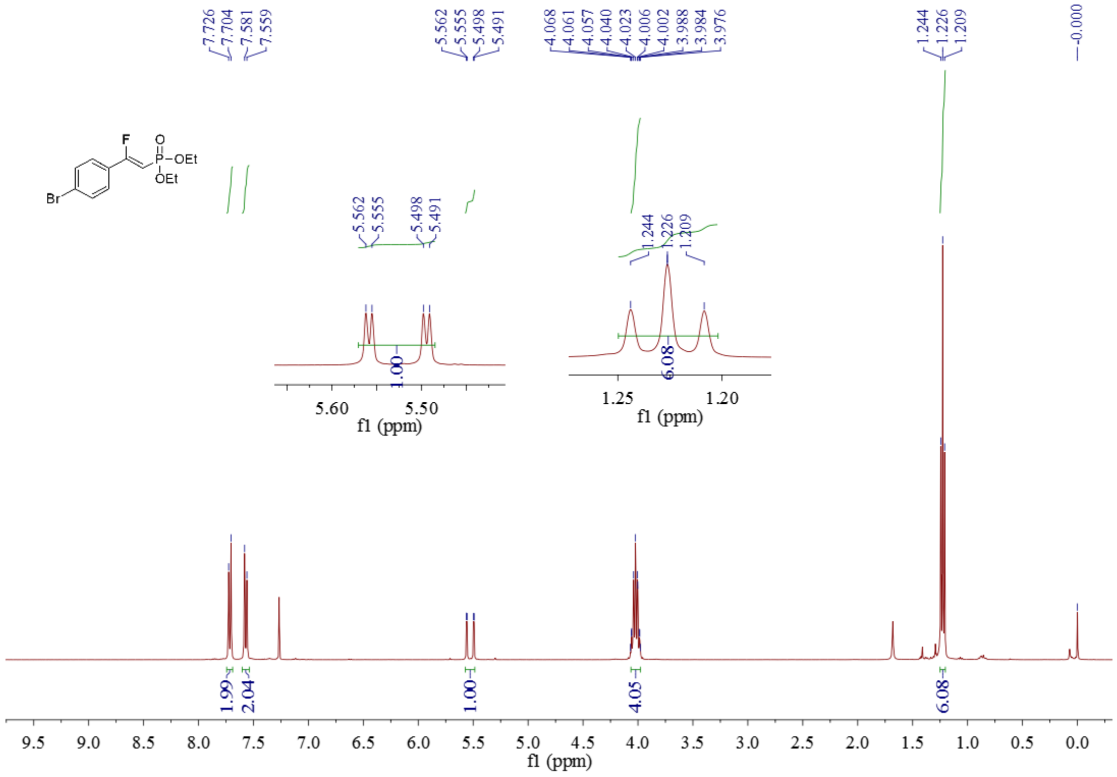

^{13}C NMR

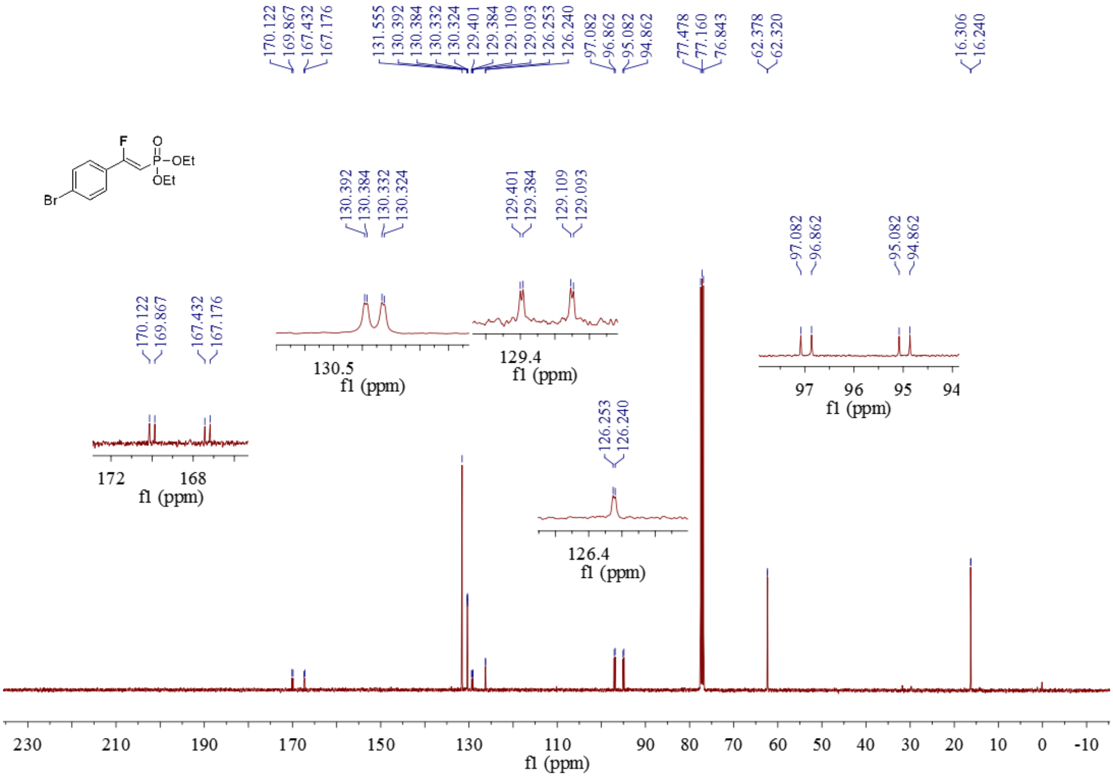

^{19}F NMR

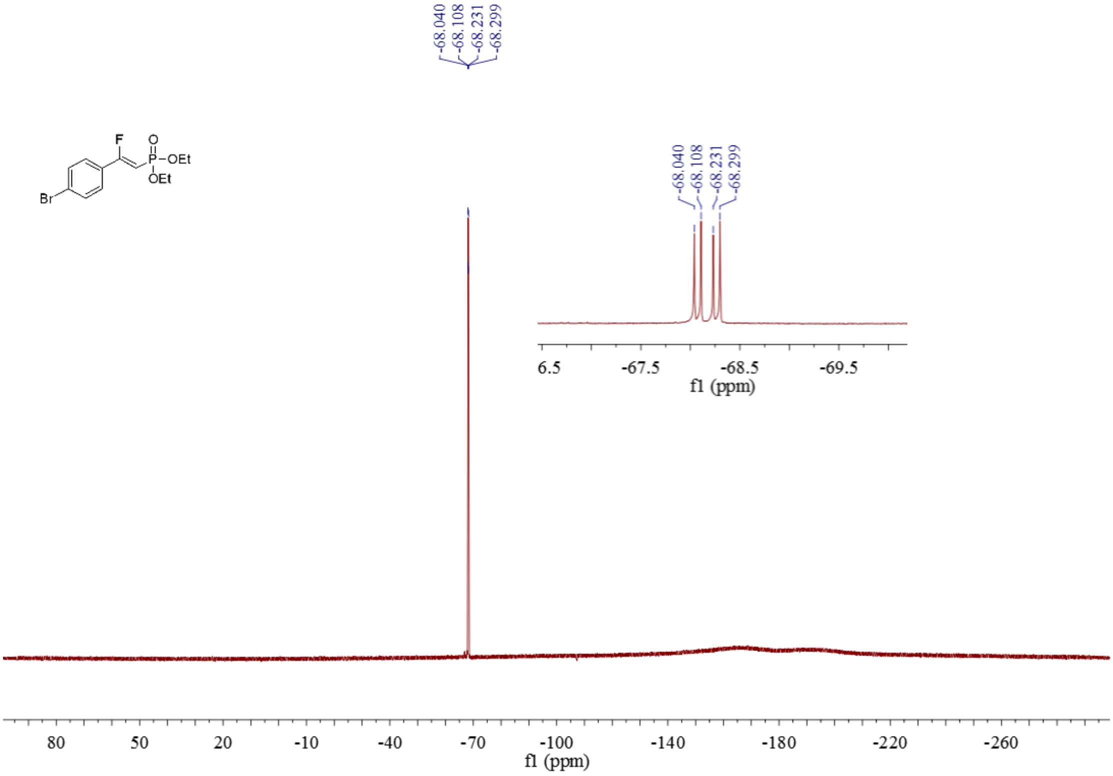

^{31}P NMR

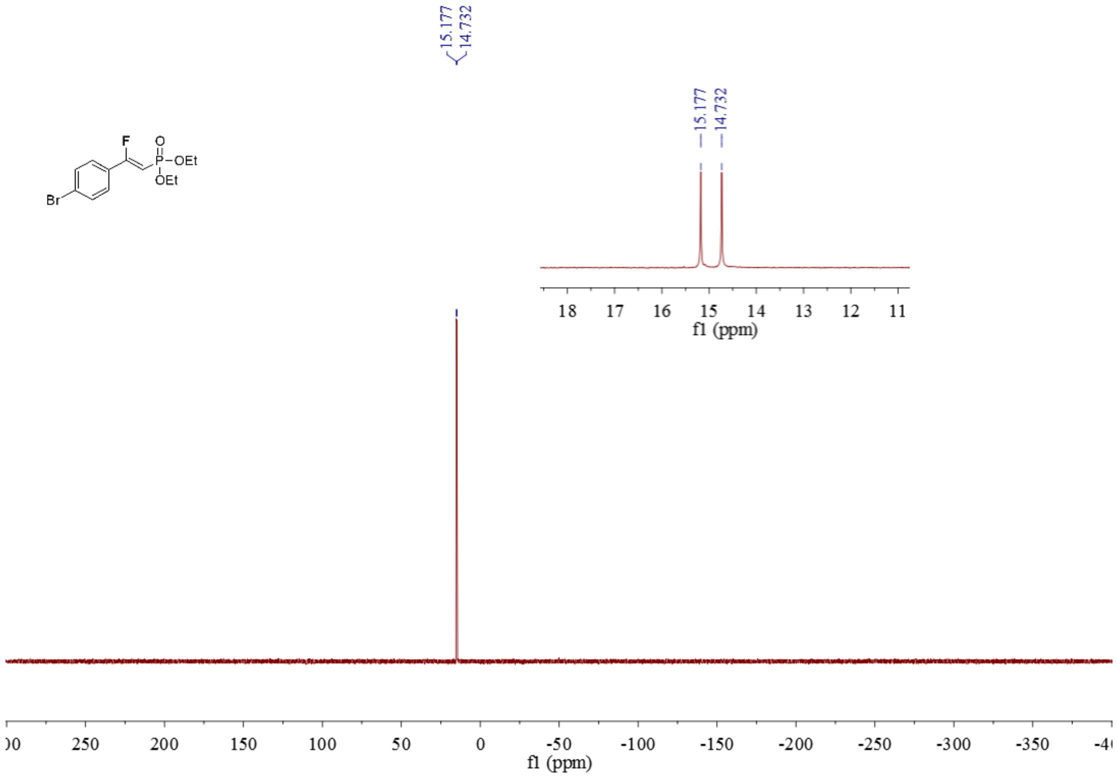

Diethyl (*Z*)-(2-fluoro-2-(4-(trifluoromethyl)phenyl)vinyl)phosphonate (2m)

^{1}H NMR

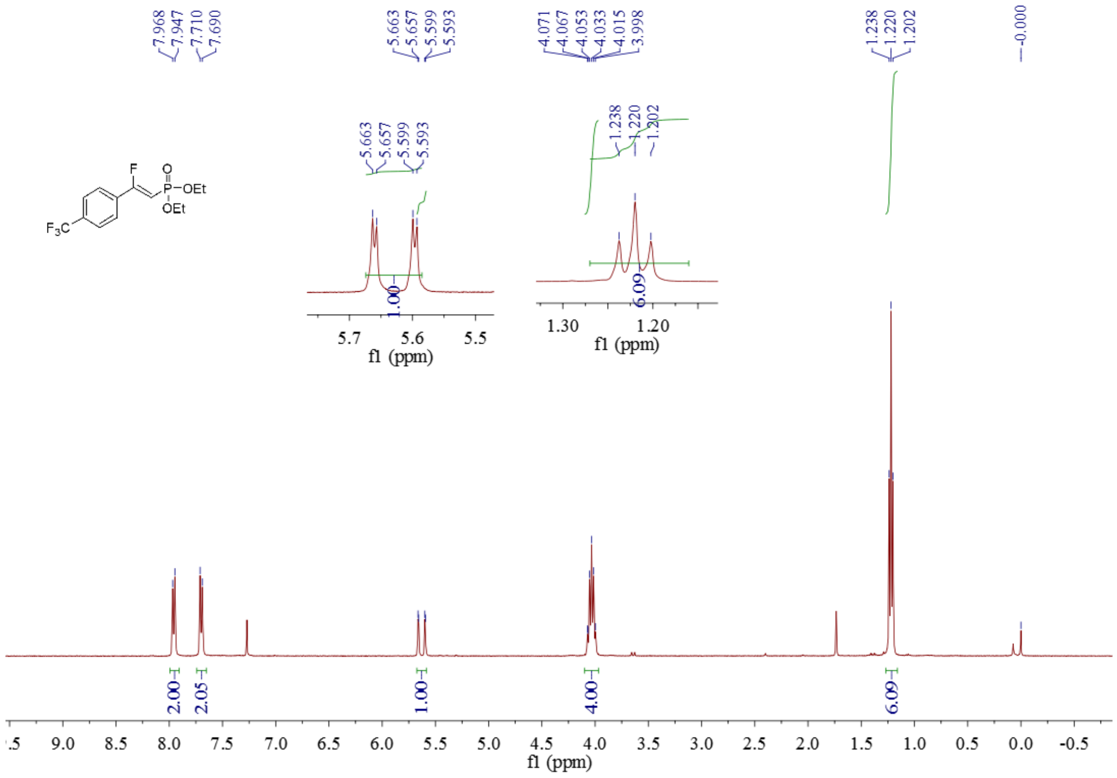

^{13}C NMR

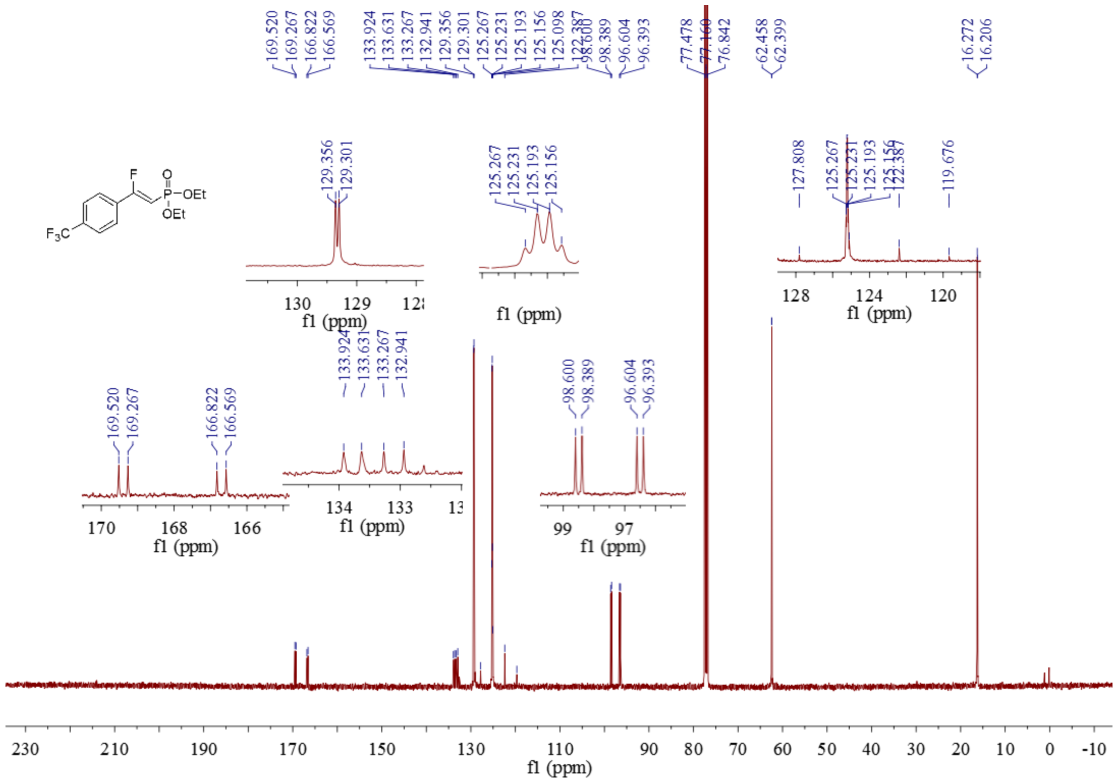

^{19}F NMR

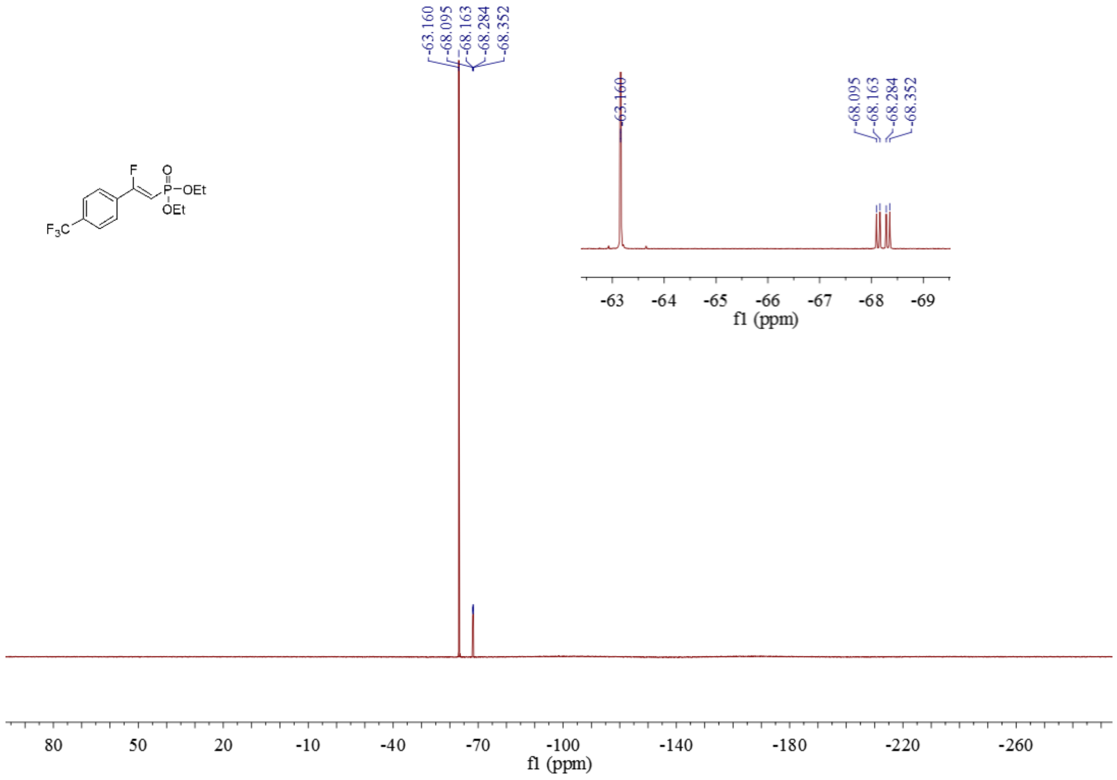

^{31}P NMR

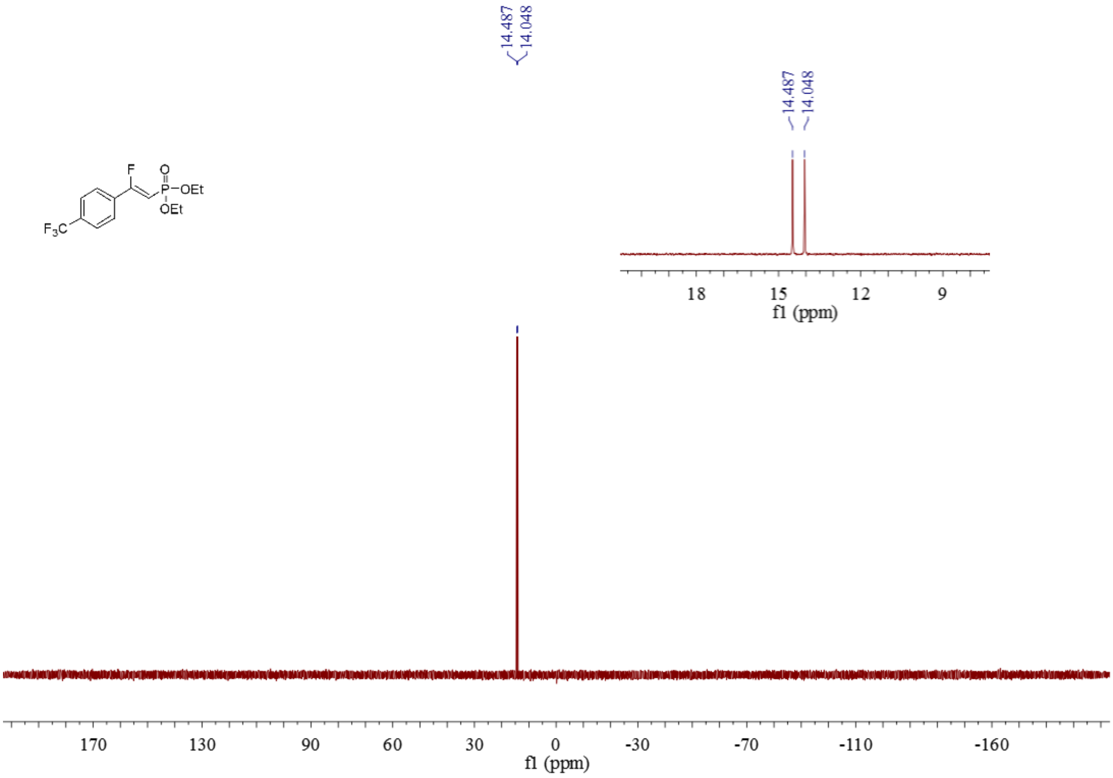

Diethyl (*Z*)-(2-(4-cyanophenyl)-2-fluorovinyl)phosphonate (2n)

^{1}H NMR

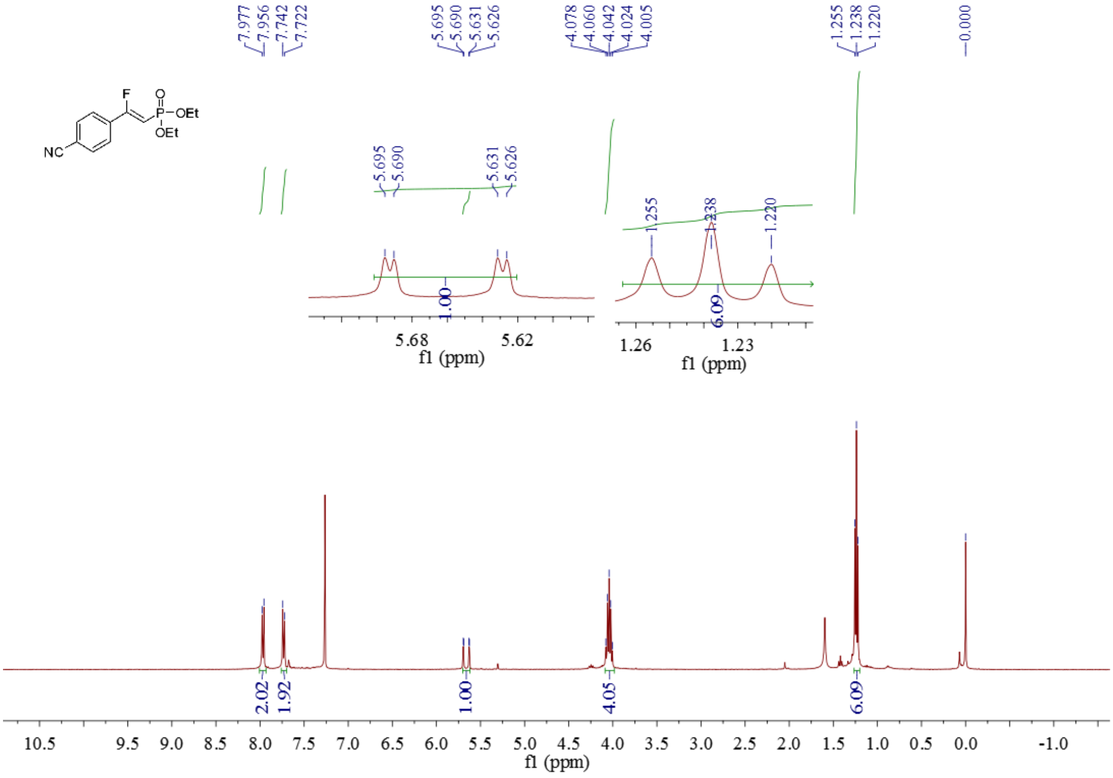

^{13}C NMR

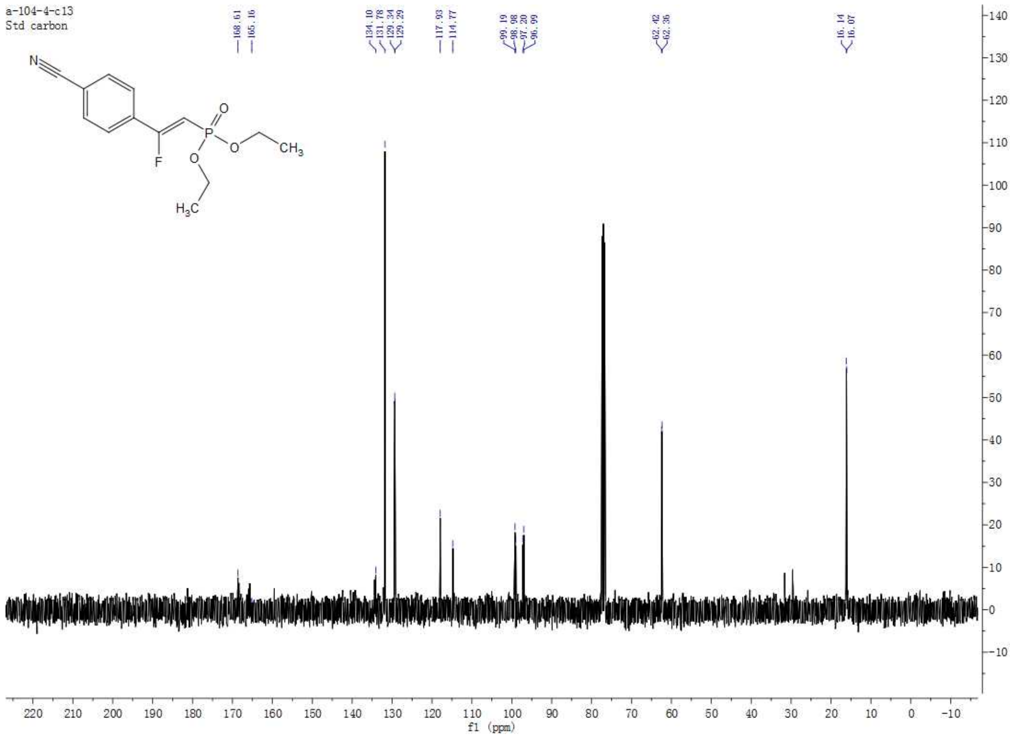

^{19}F NMR

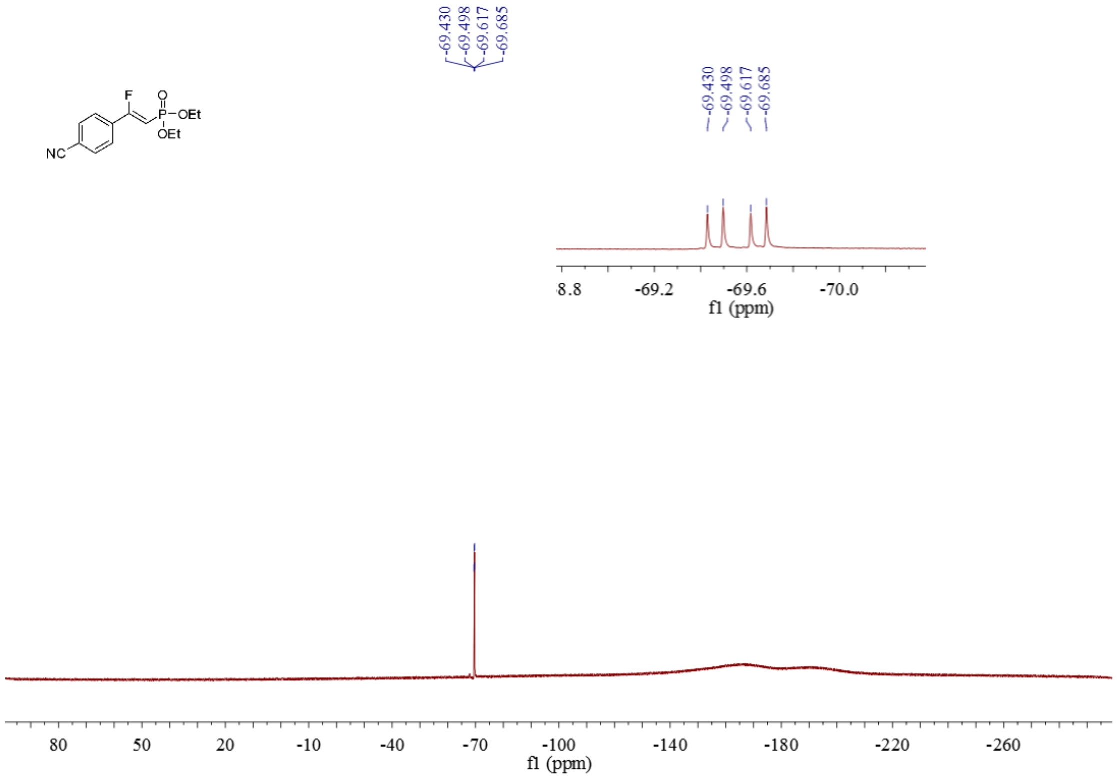

^{31}P NMR

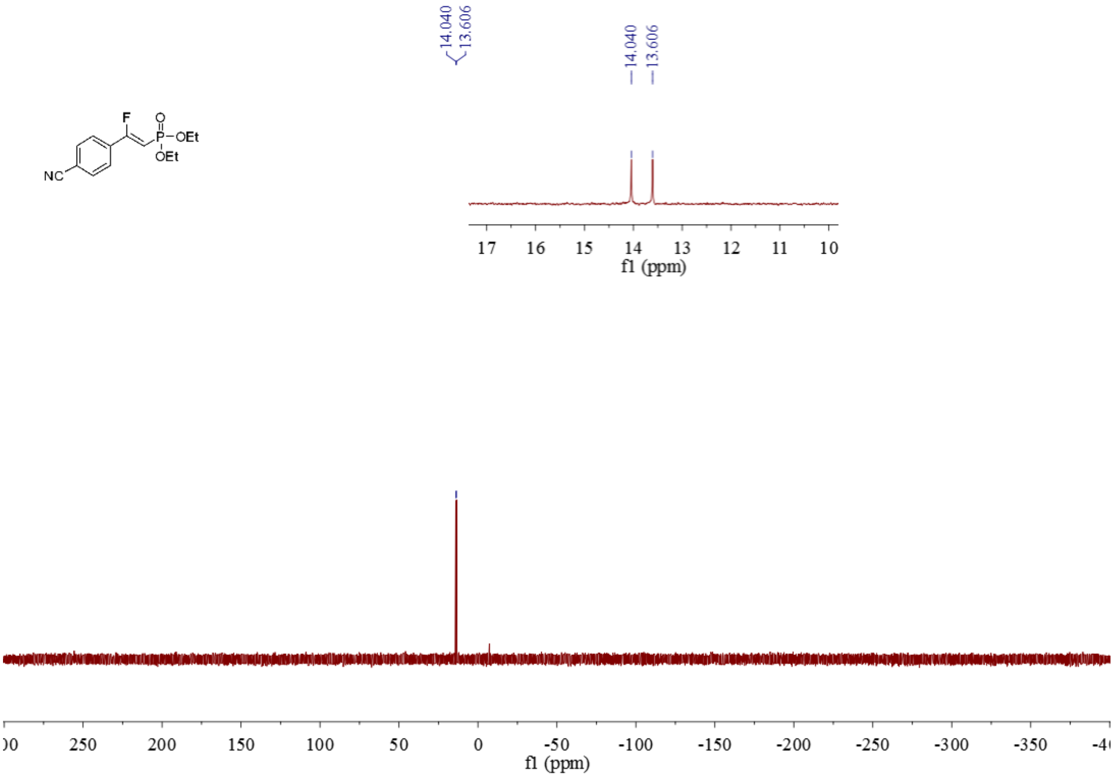

Methyl (*Z*)-4-(2-(diethoxyphosphoryl)-1-fluorovinyl)benzoate (2o)

^{1}H NMR

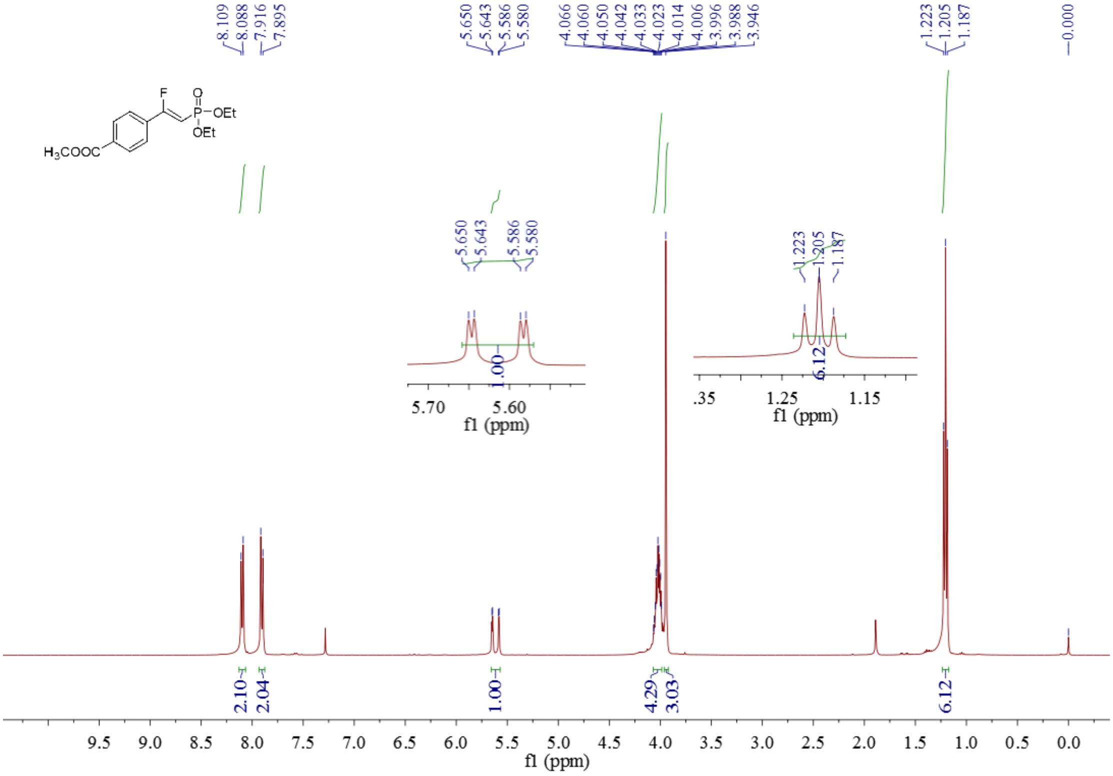

^{13}C NMR

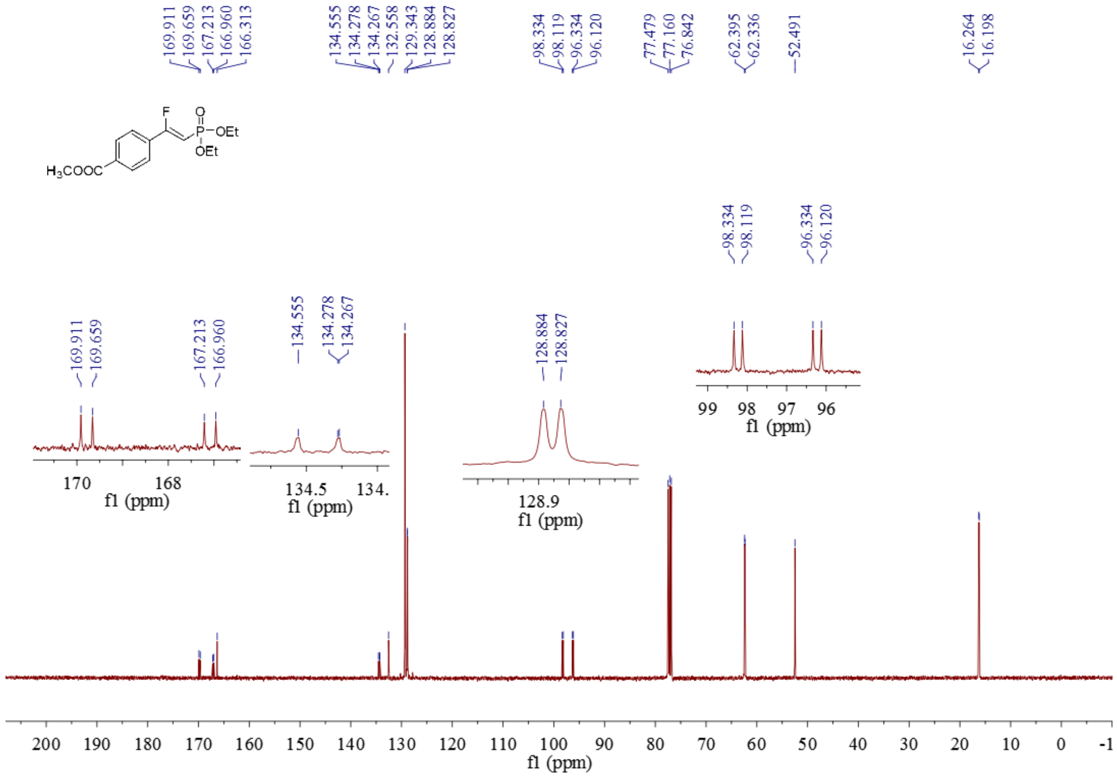

^{19}F NMR

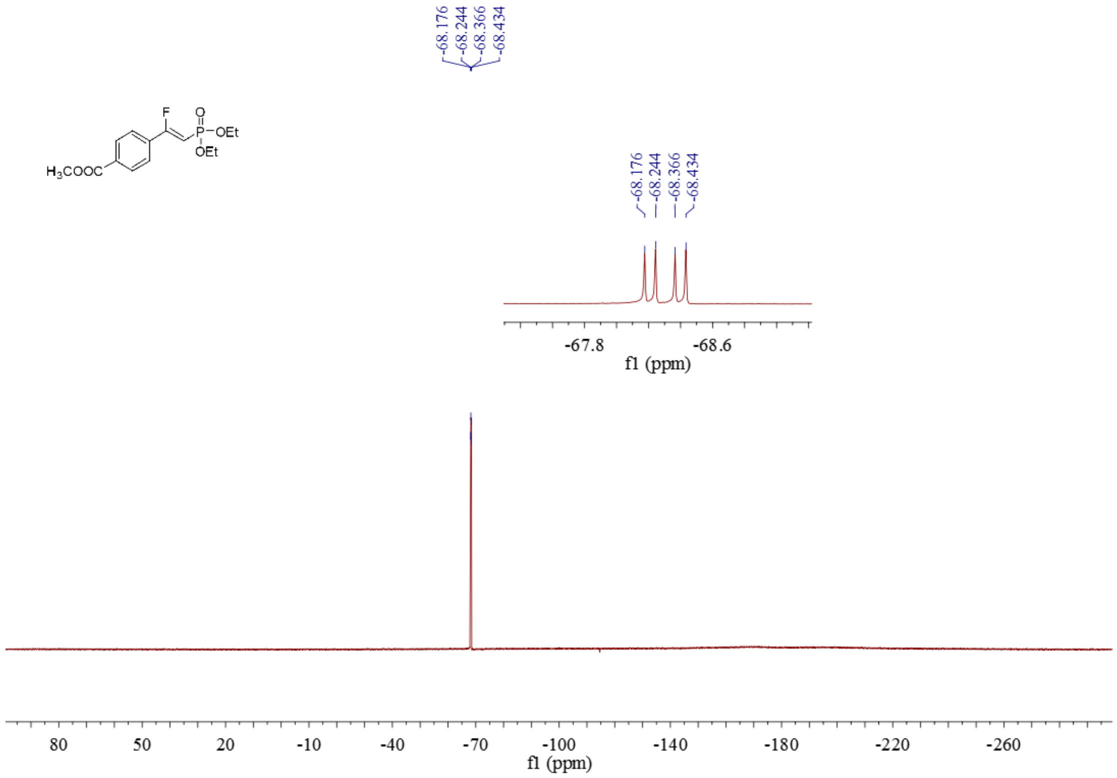

^{31}P NMR

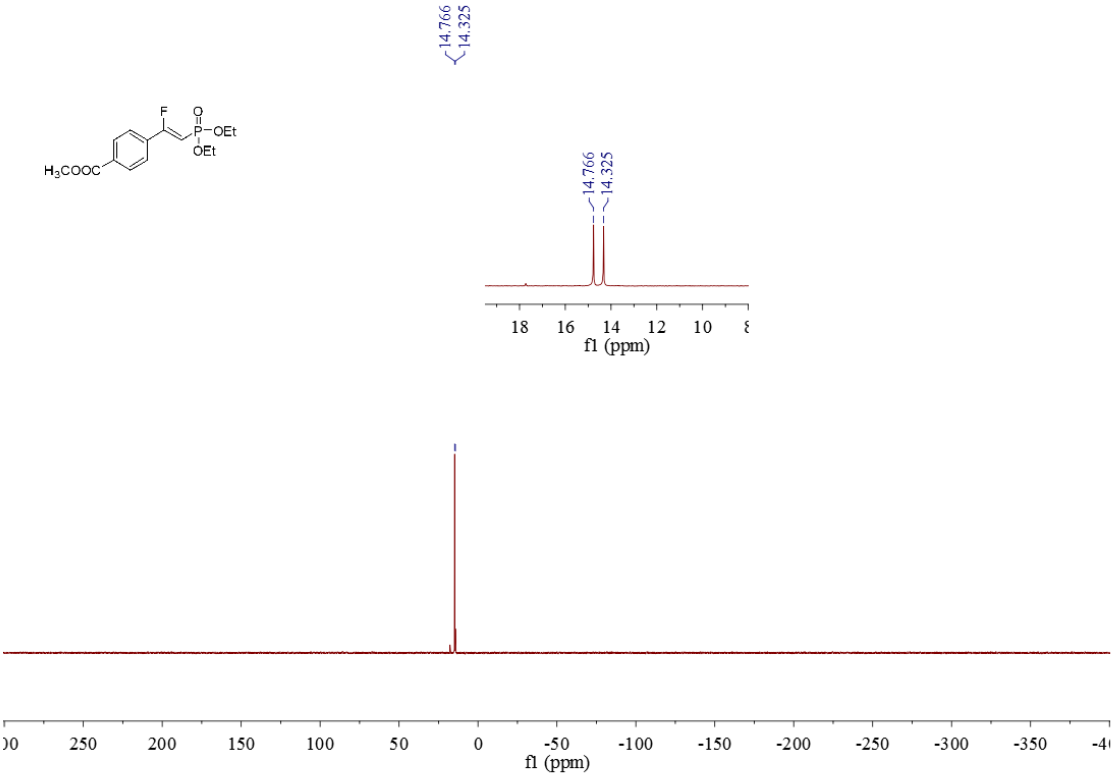

Diethyl (*Z*)-(1-fluoro-1-phenylprop-1-en-2-yl)phosphonate (2p)

^{1}H NMR

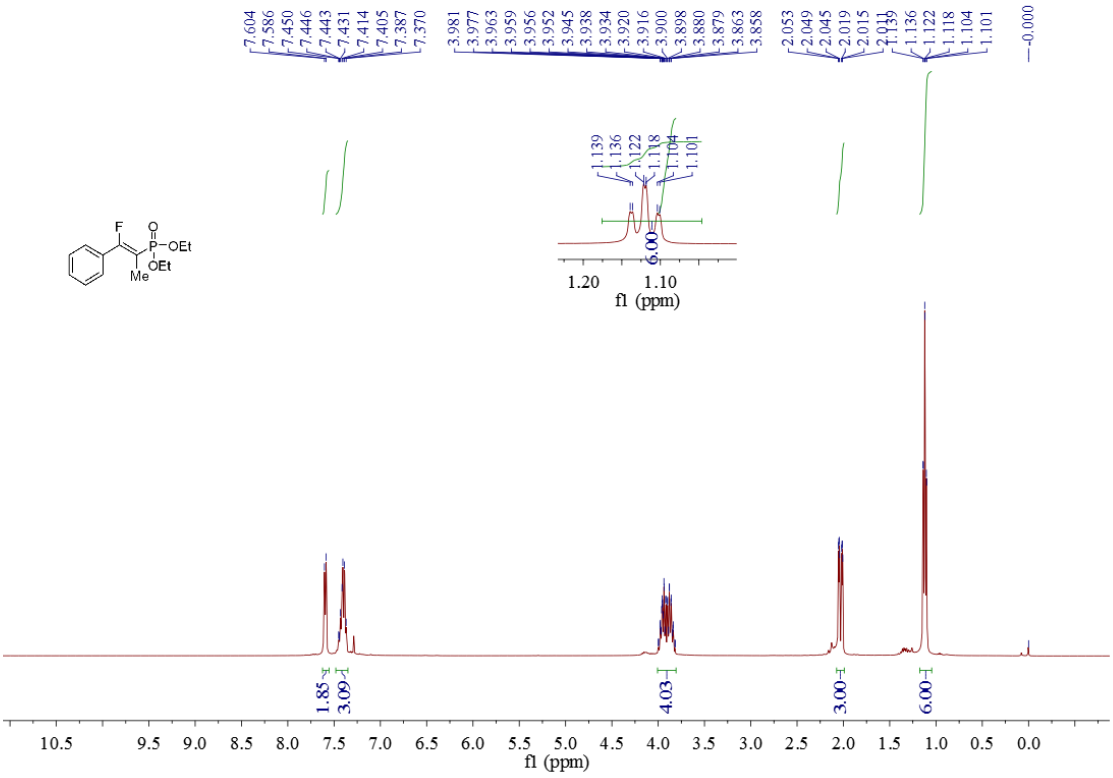

^{13}C NMR

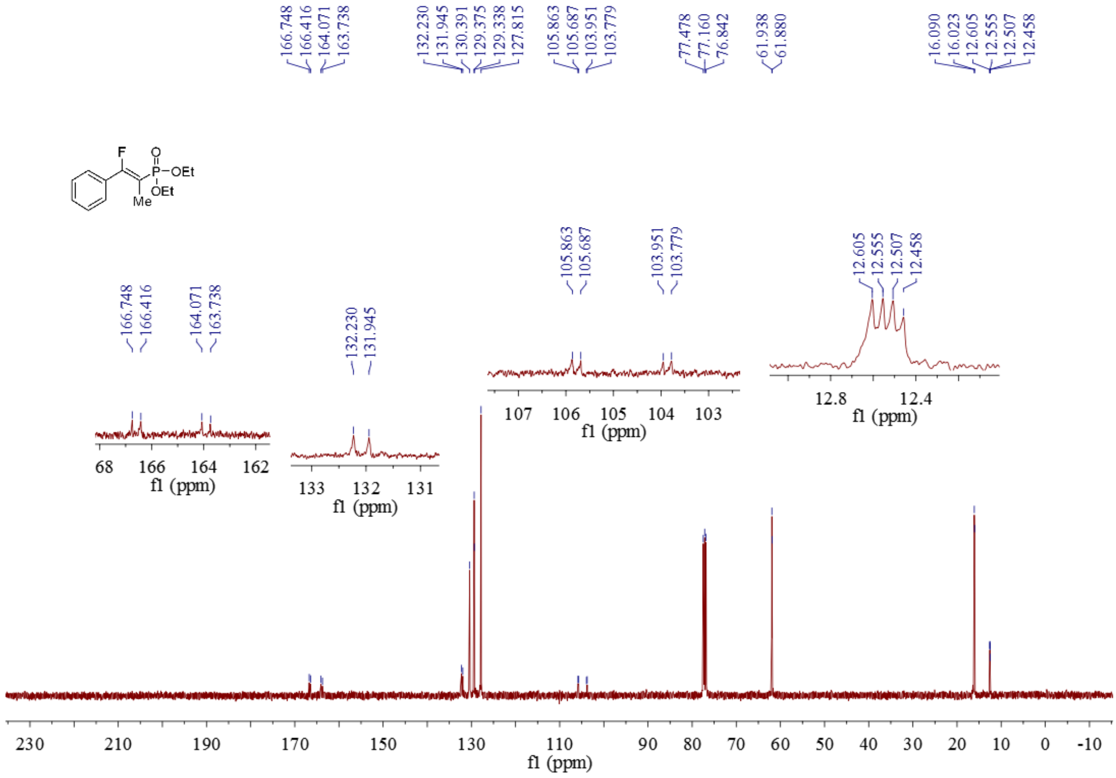

^{19}F NMR

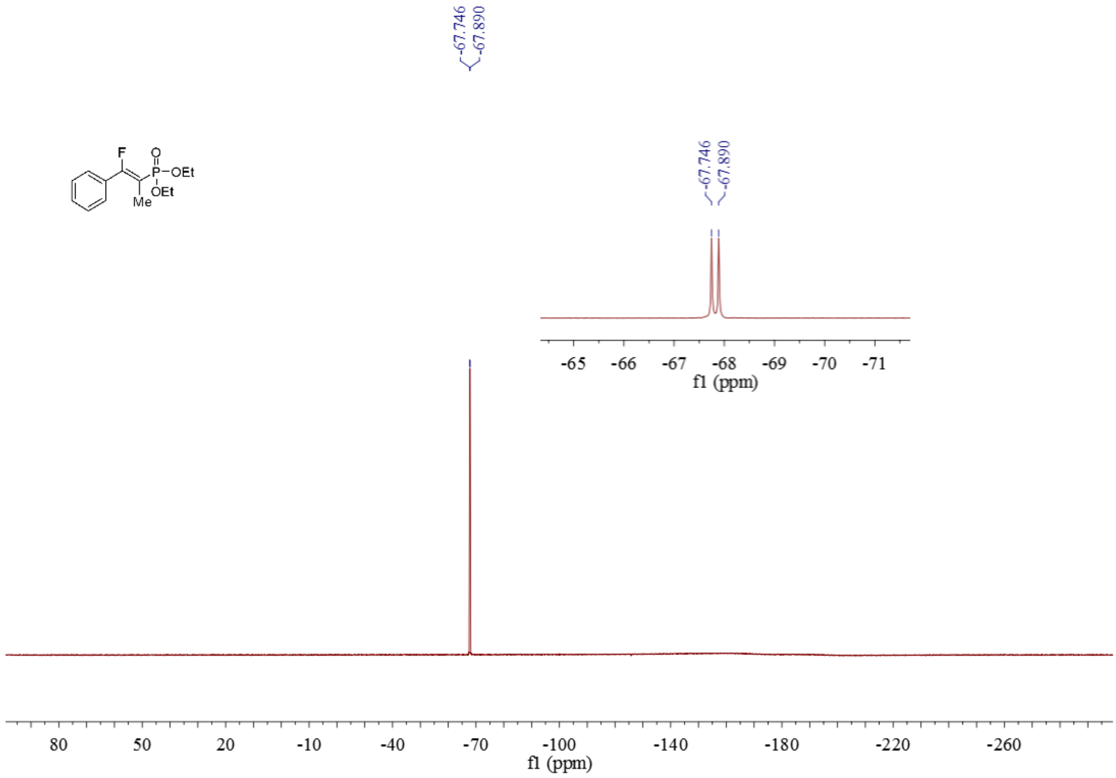

^{31}P NMR

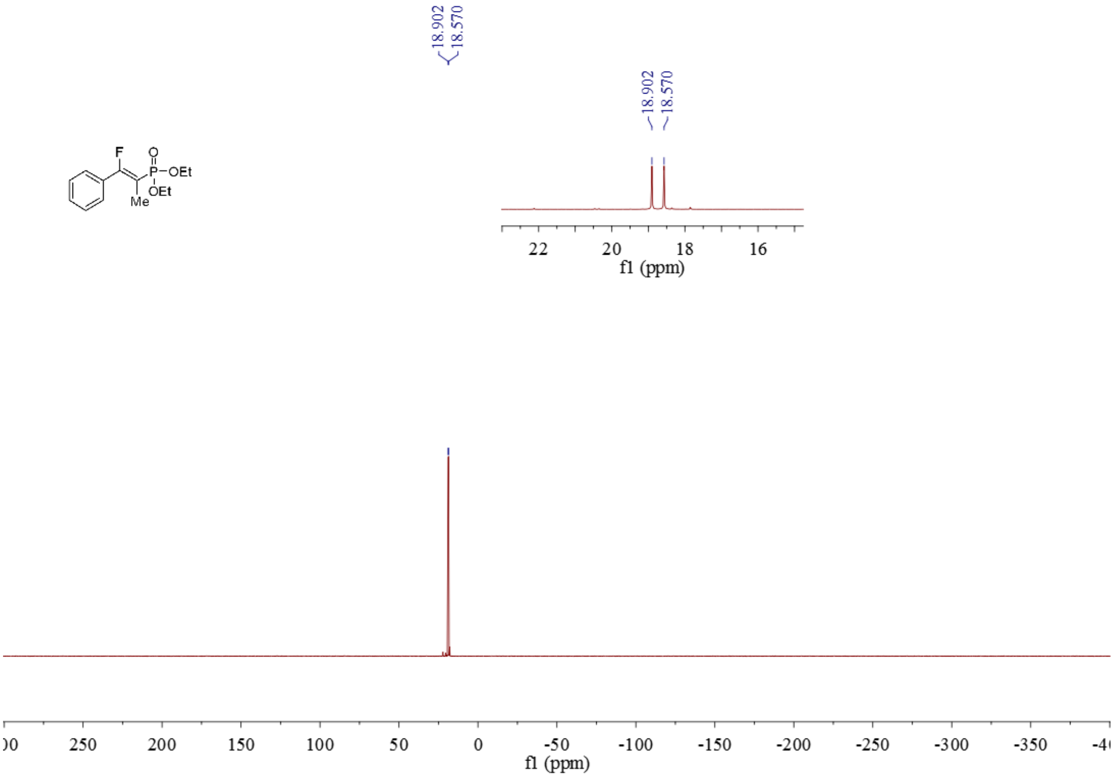

Diethyl (*Z*)-(1-fluoro-1-phenylbut-1-en-2-yl)phosphonate (2q)

^{1}H NMR

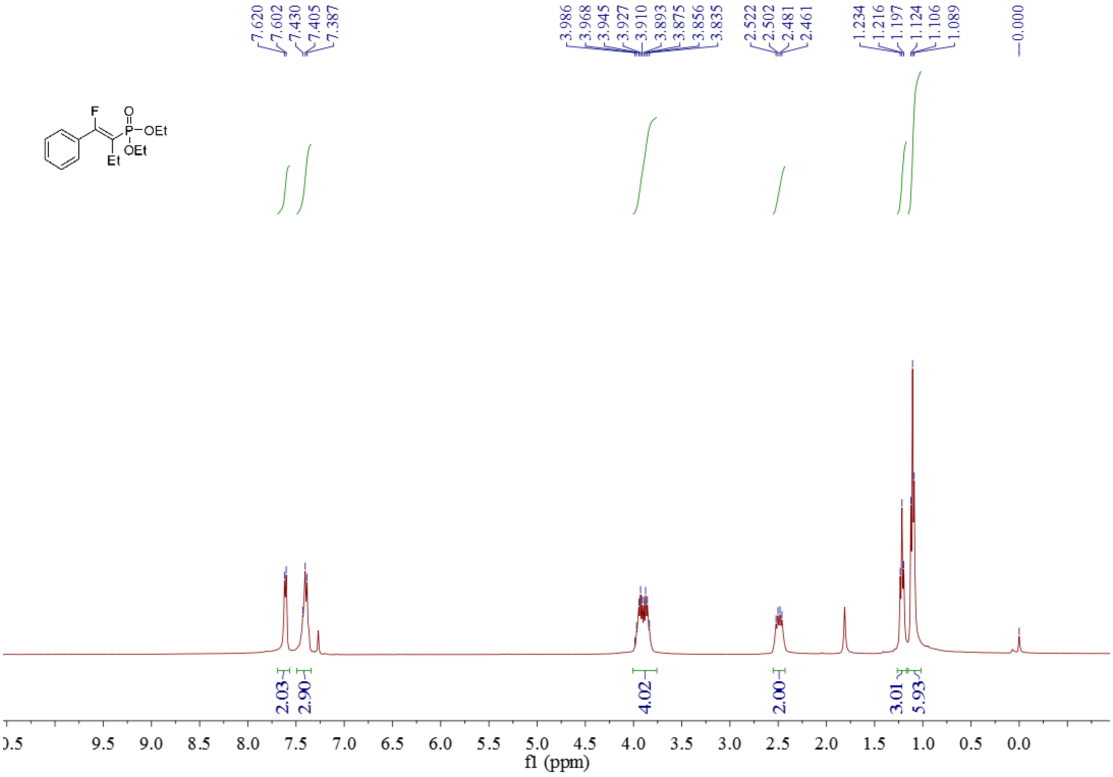

^{13}C NMR

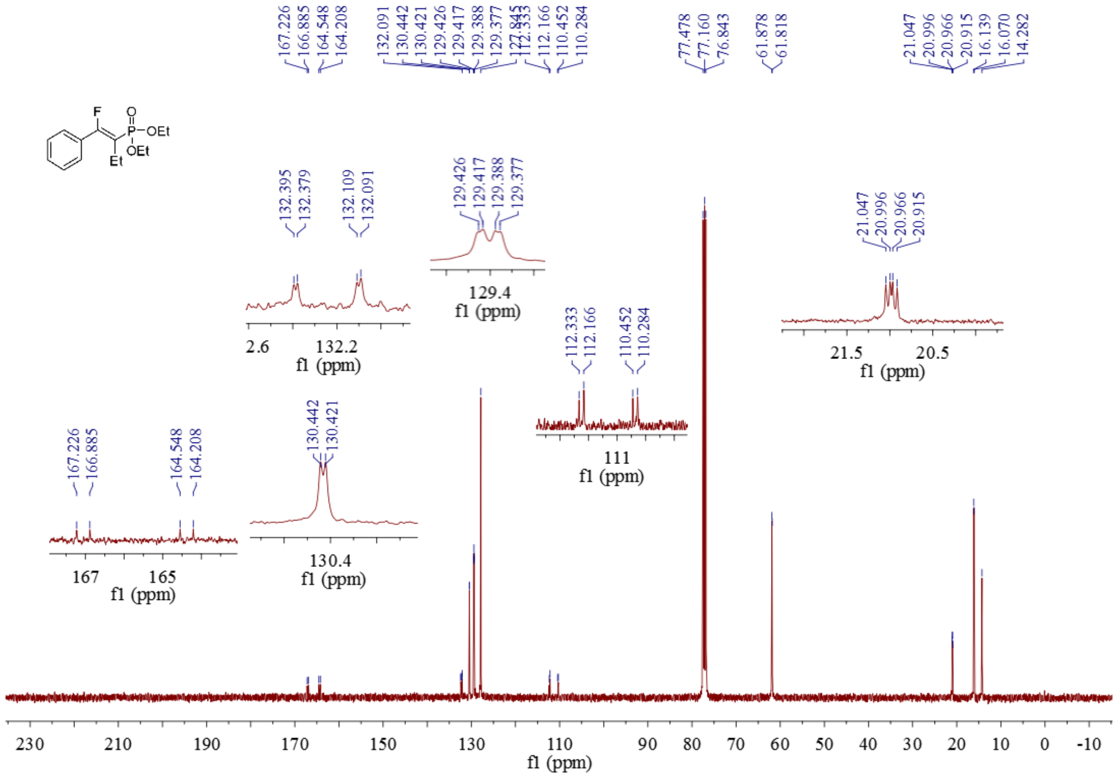

^{19}F NMR

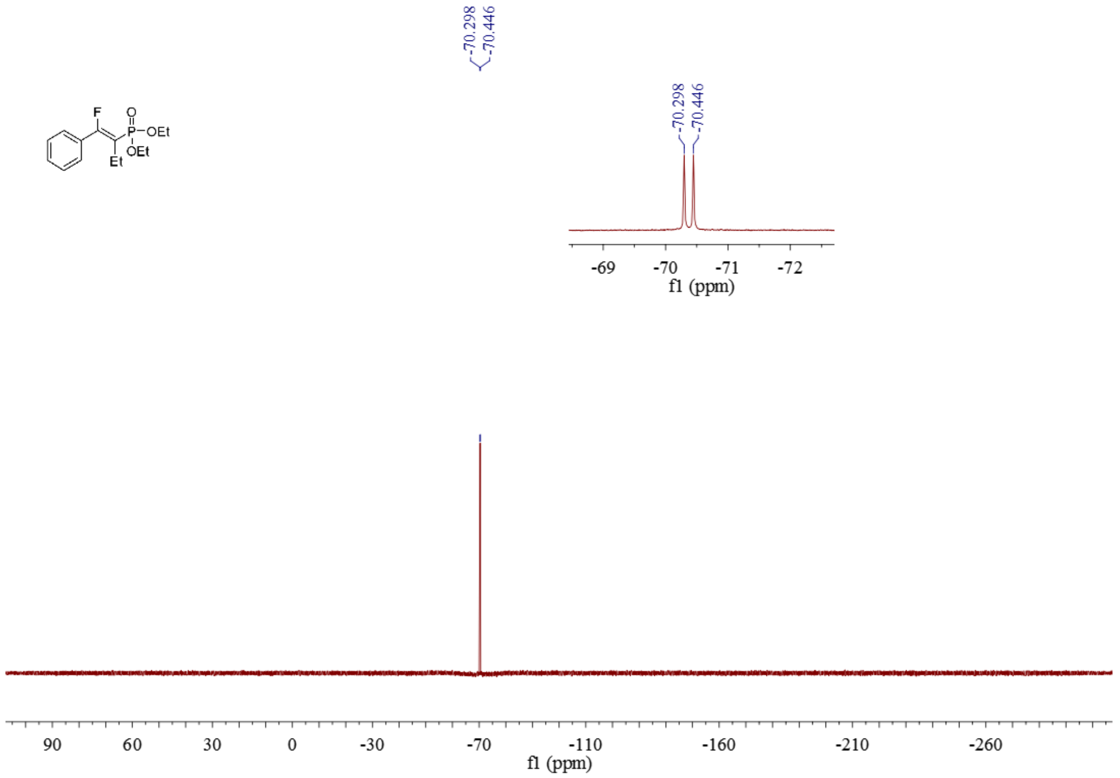

^{31}P NMR

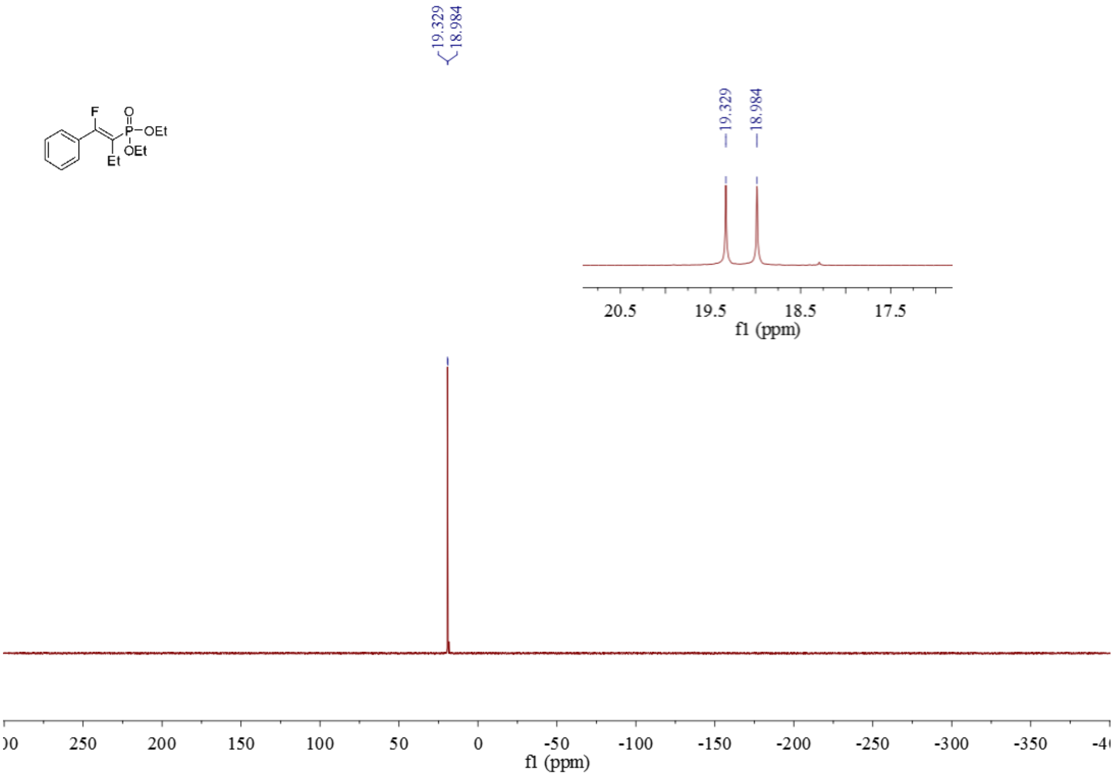

Dimethyl (*Z*)-(2-fluoro-2-phenylvinyl)phosphonate (3a)

^{1}H NMR

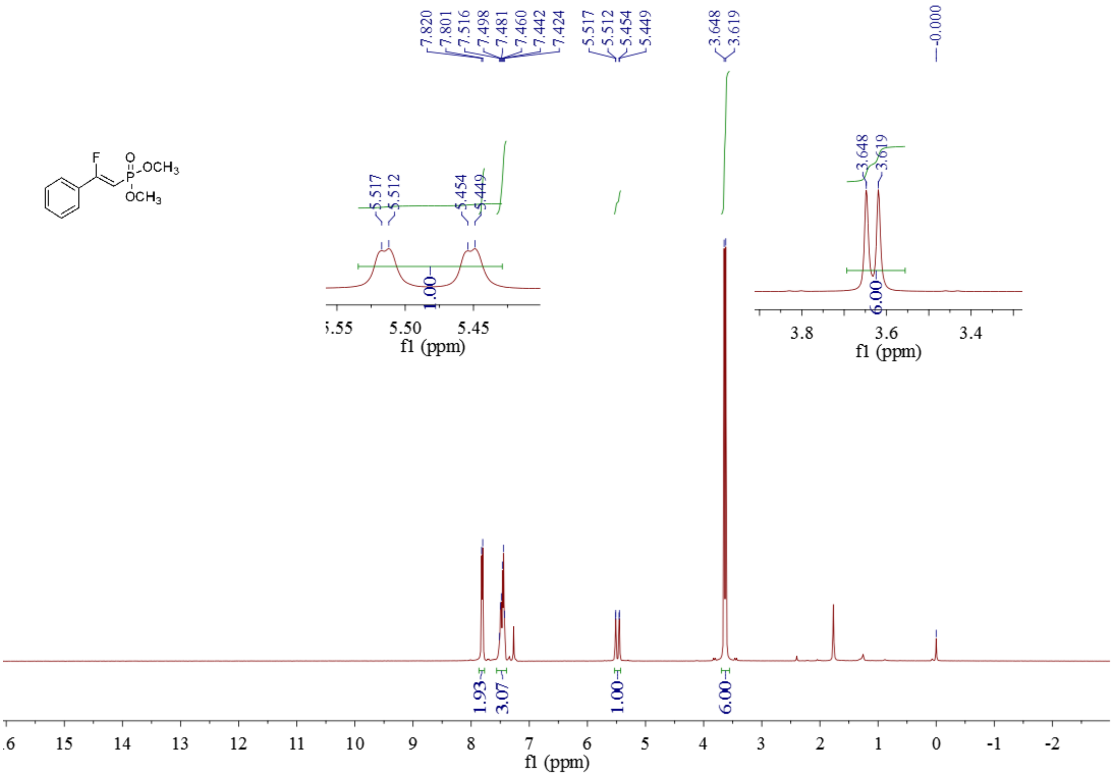

^{13}C NMR

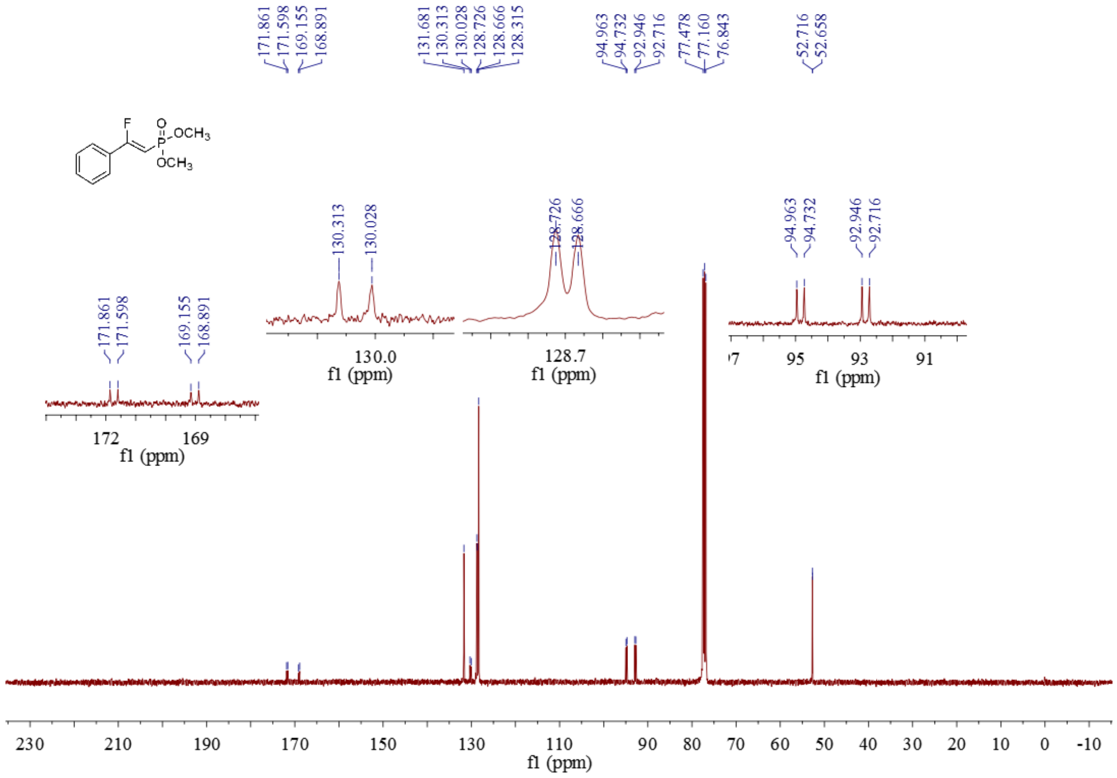

^{19}F NMR

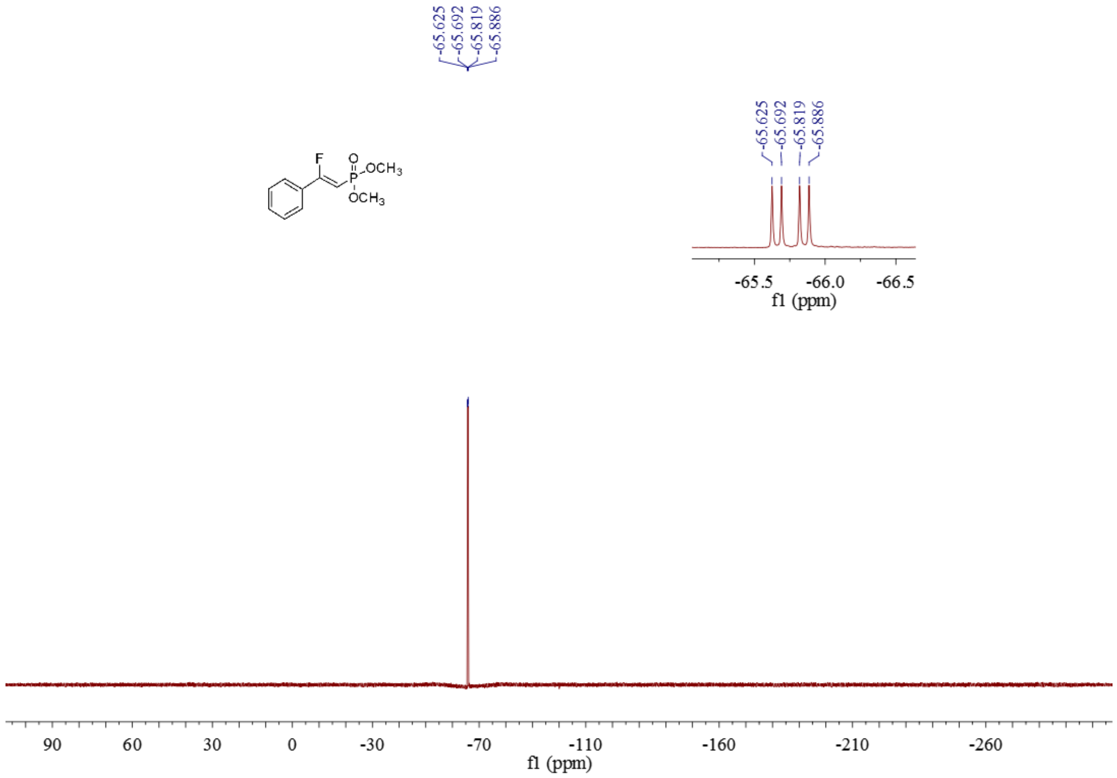

^{31}P NMR

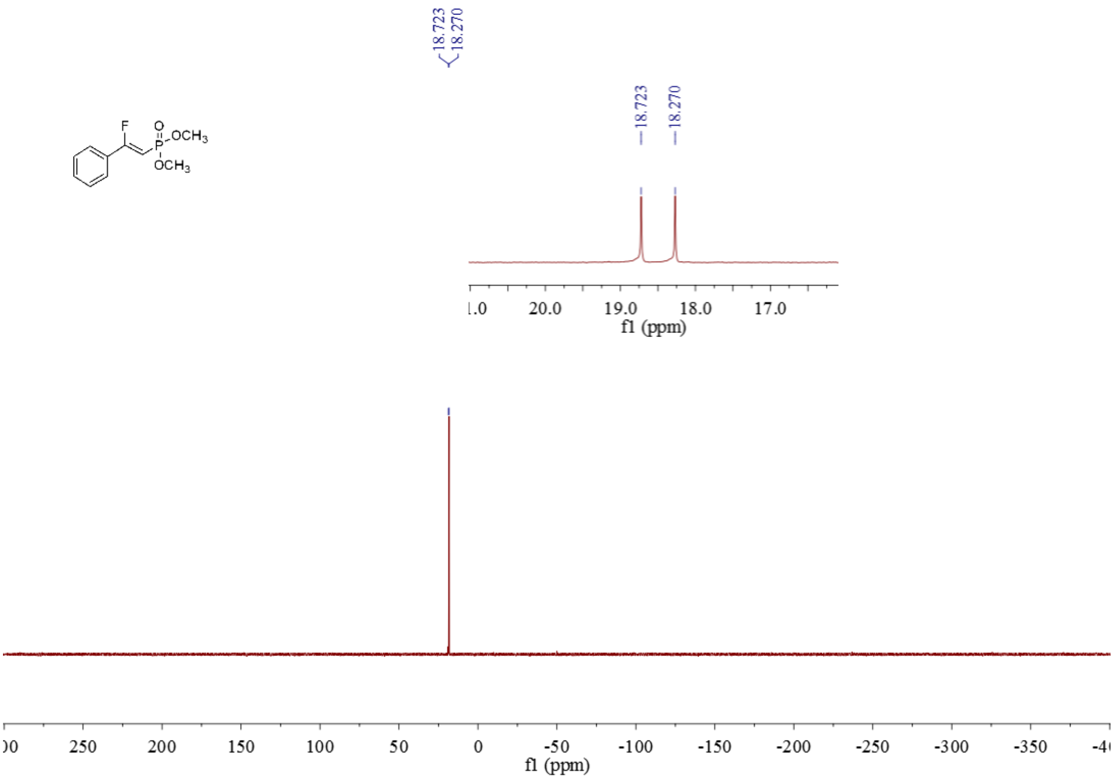

Dimethyl (*Z*)-(2-fluoro-2-(*p*-tolyl)vinyl) phosphonate (3b)

^{1}H NMR

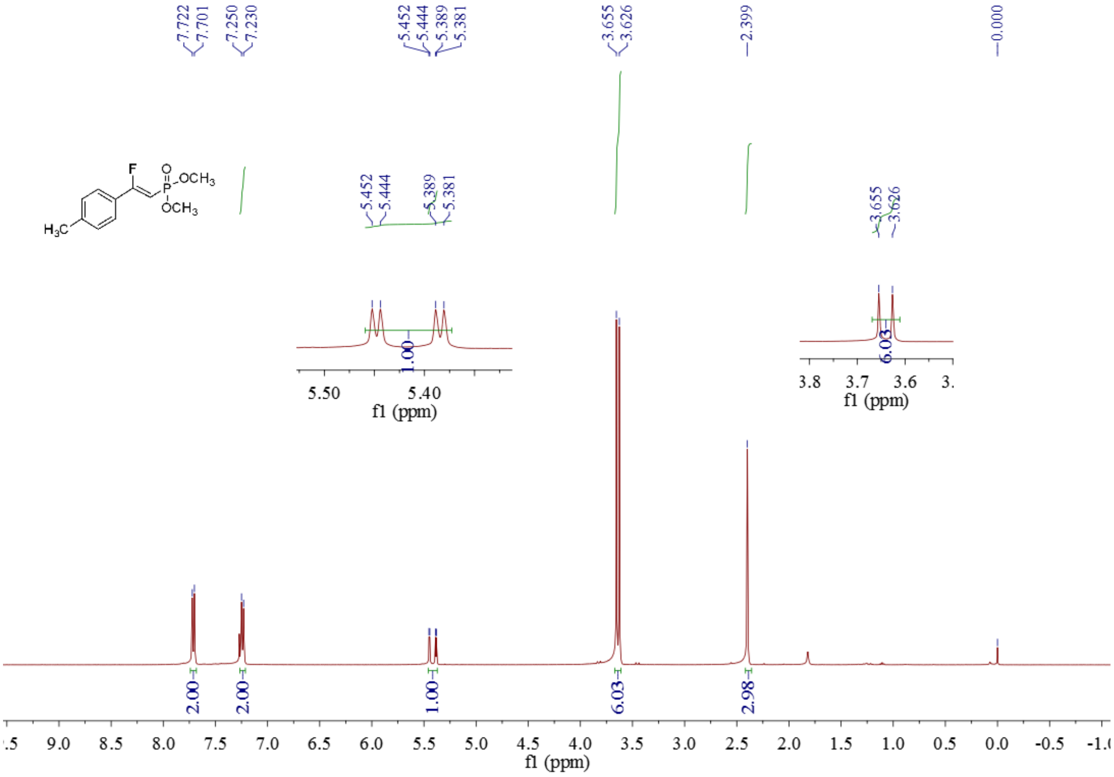

^{13}C NMR

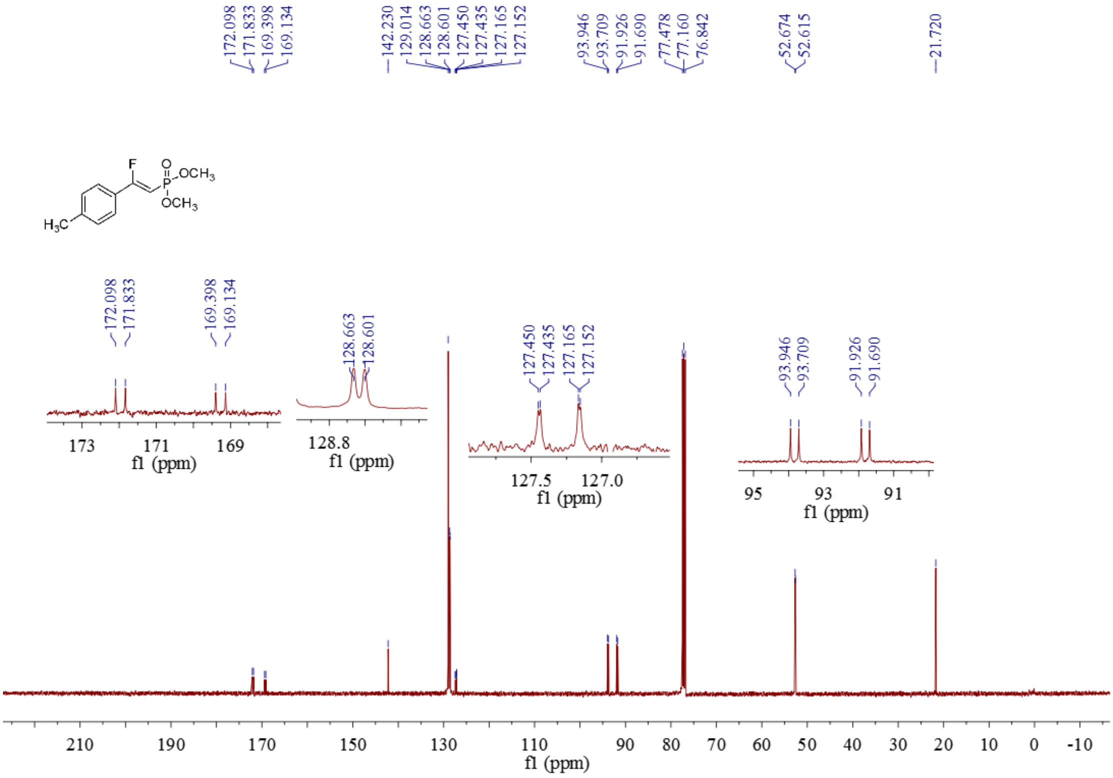

^{19}F NMR

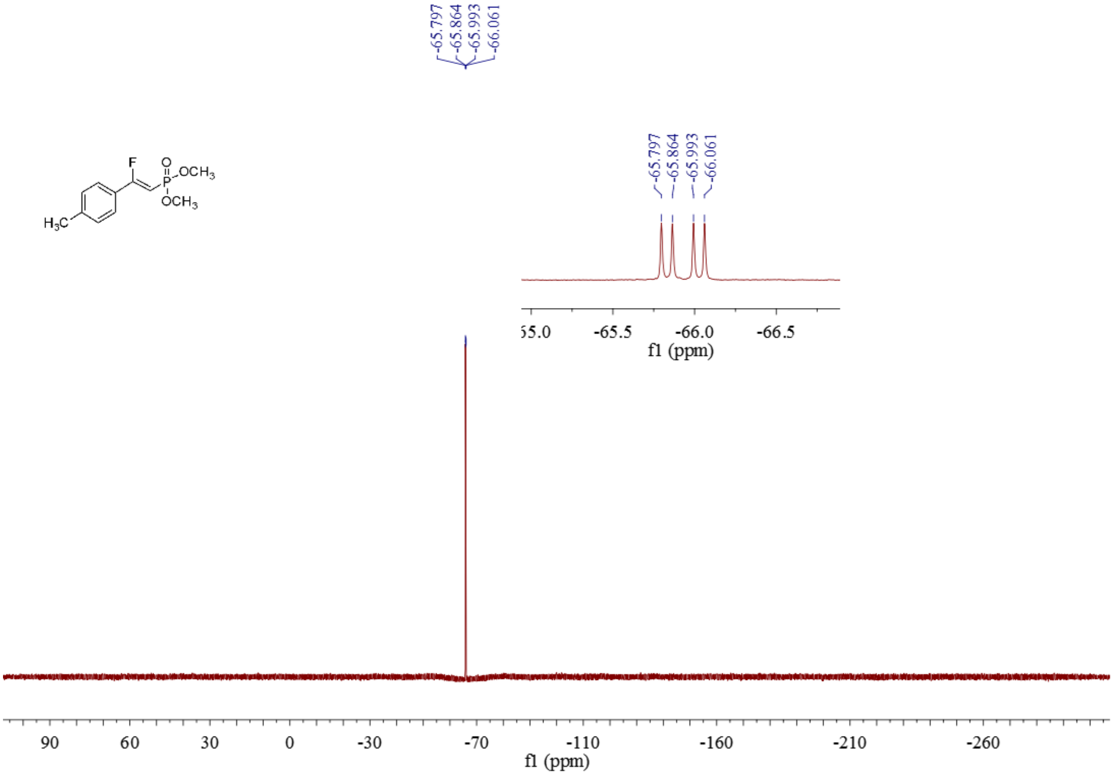

^{31}P NMR

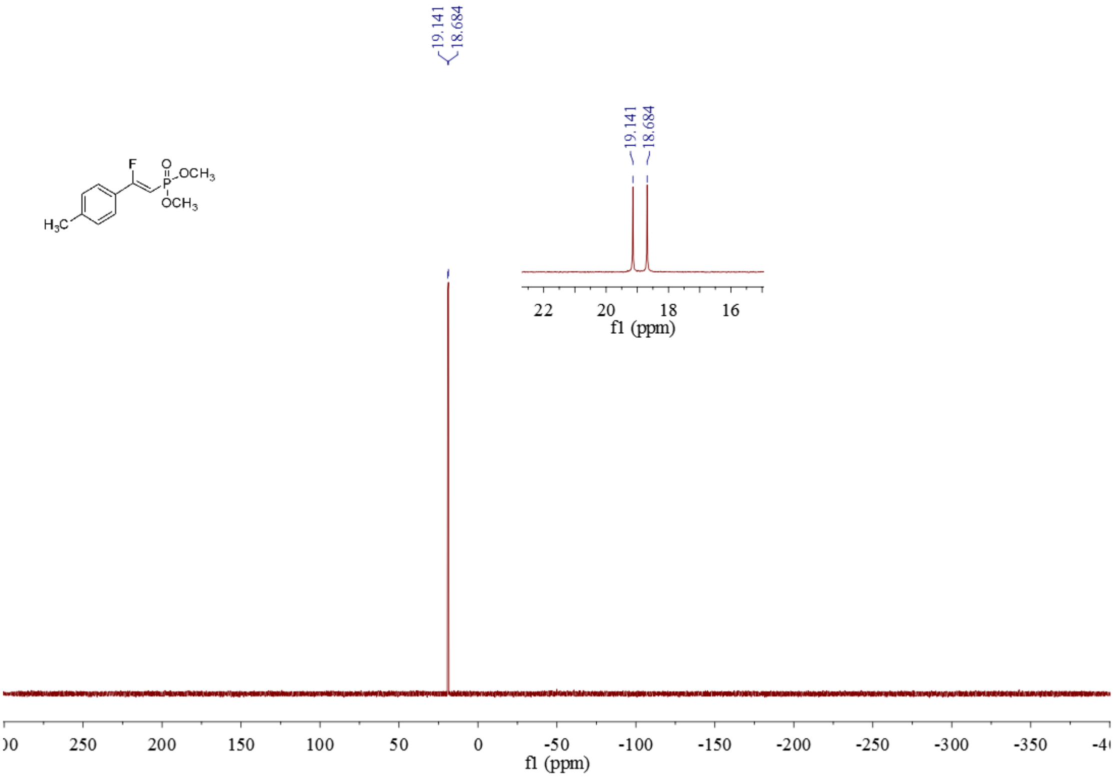

Dimethyl (*Z*)-(2-fluoro-2-(4-fluorophenyl)vinyl) phosphonate (3c)

^{1}H NMR

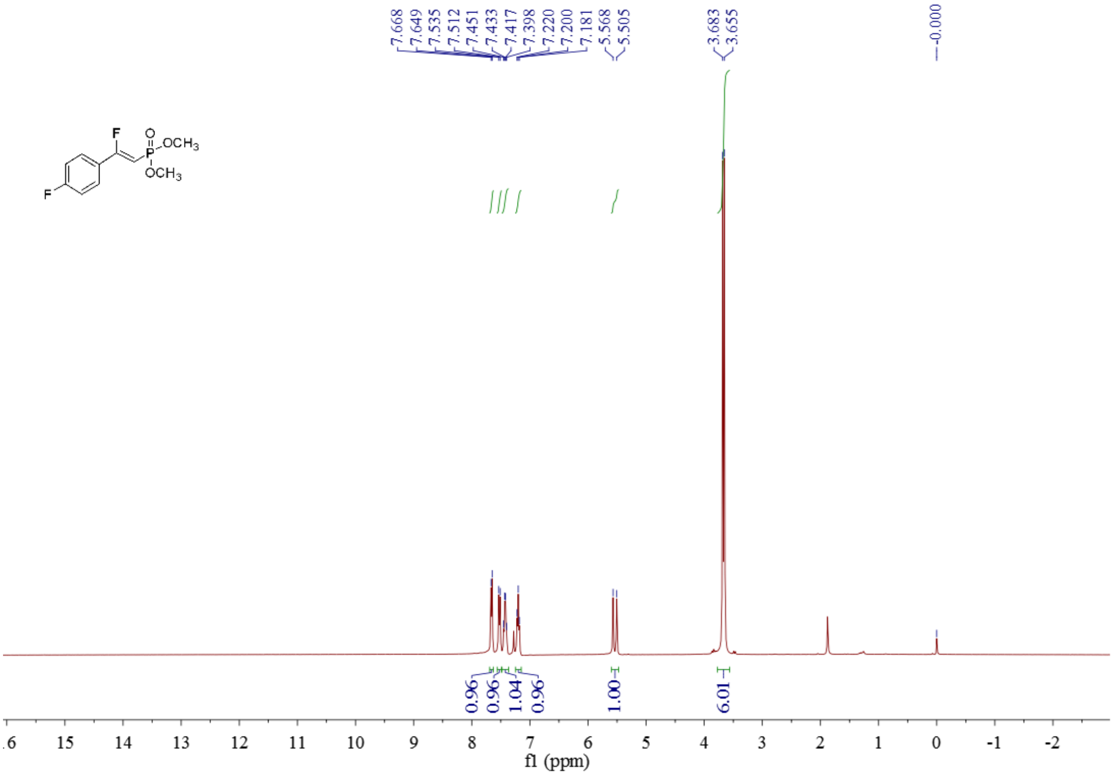

^{13}C NMR

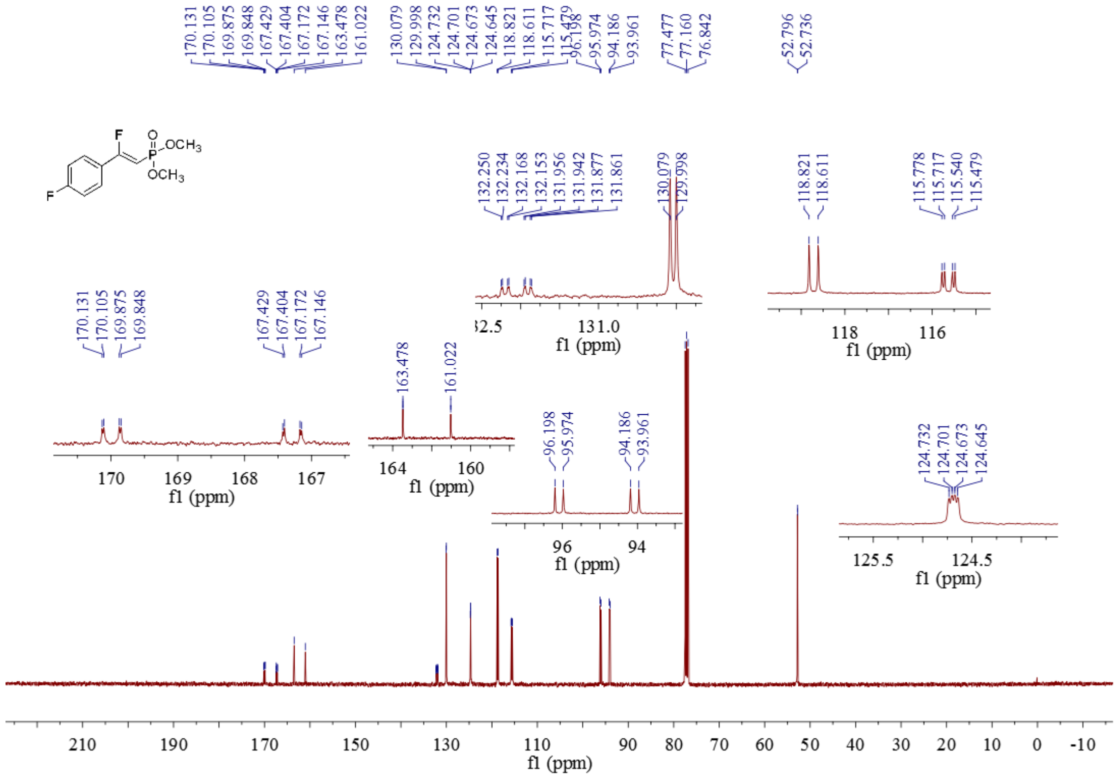

^{19}F NMR

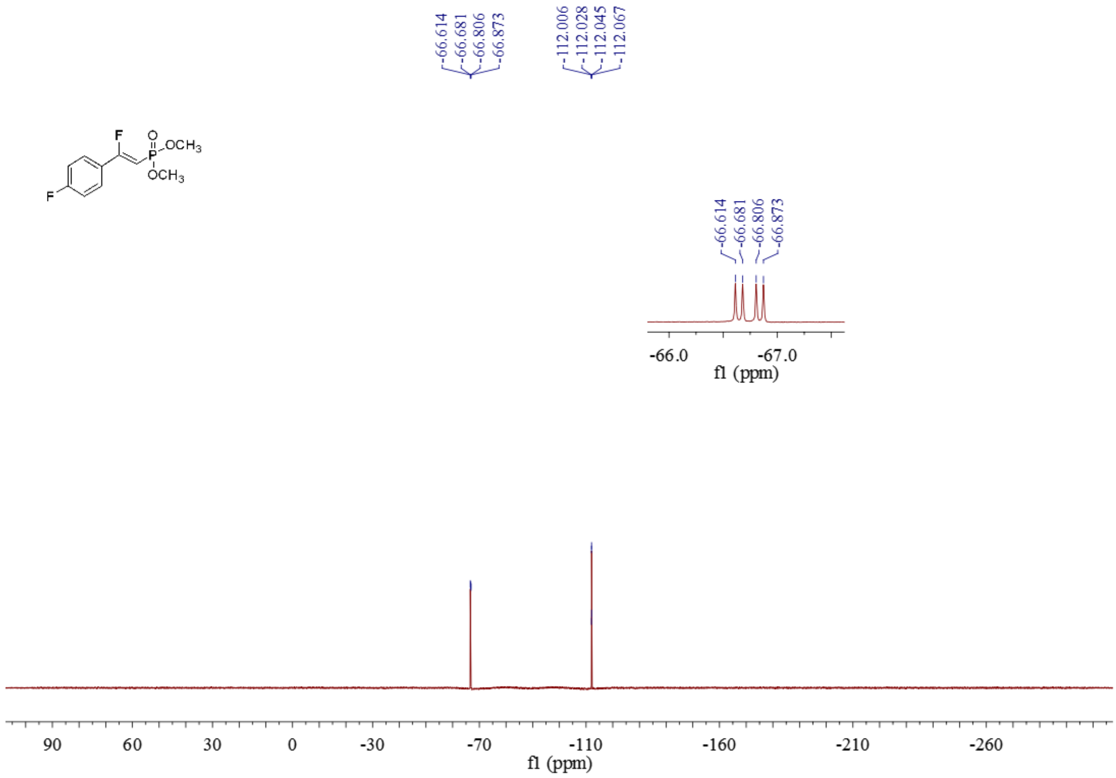

^{31}P NMR

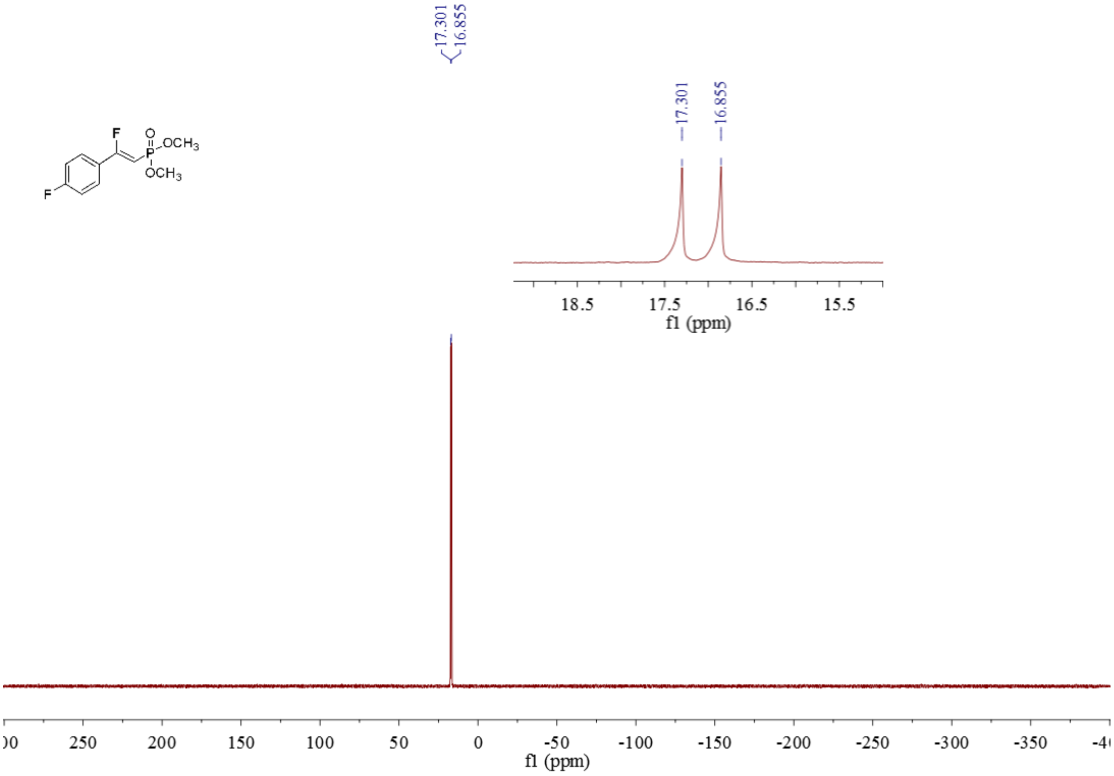

17.301
16.855
F
O
P
OCH3
OCH3
F
17.301
16.855
18.5
17.5
16.5
15.5
f1 (ppm)
250
200
150
100
50
0
-50
-100
-150
-200
-250
-300
-350
f1 (ppm)

Dimethyl (*Z*)-(2-(4-bromophenyl)-2-fluorovinyl)phosphonate (3d)

^{1}H NMR

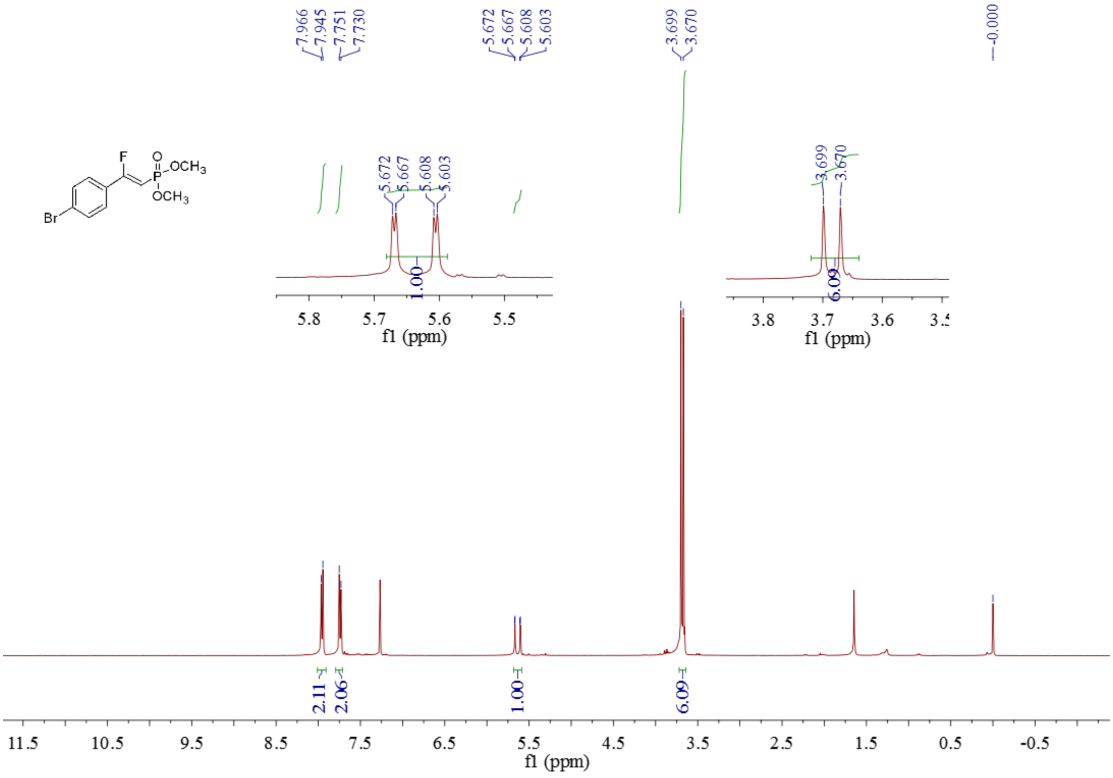

^{13}C NMR

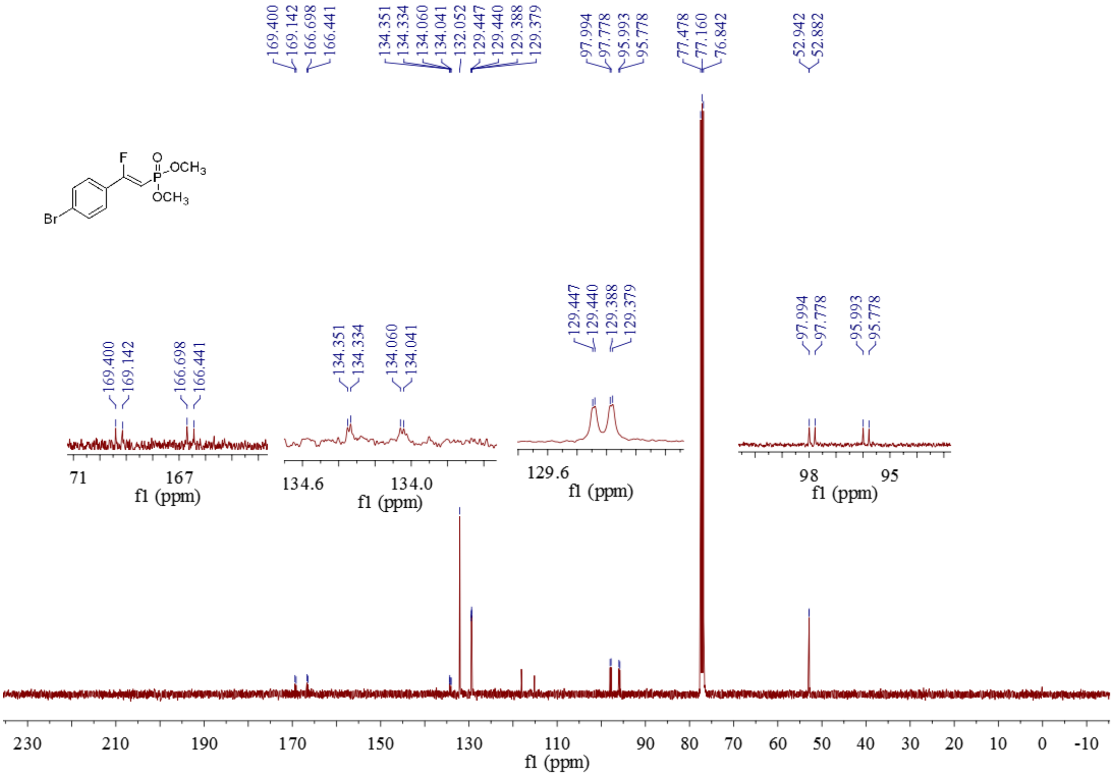

^{19}F NMR

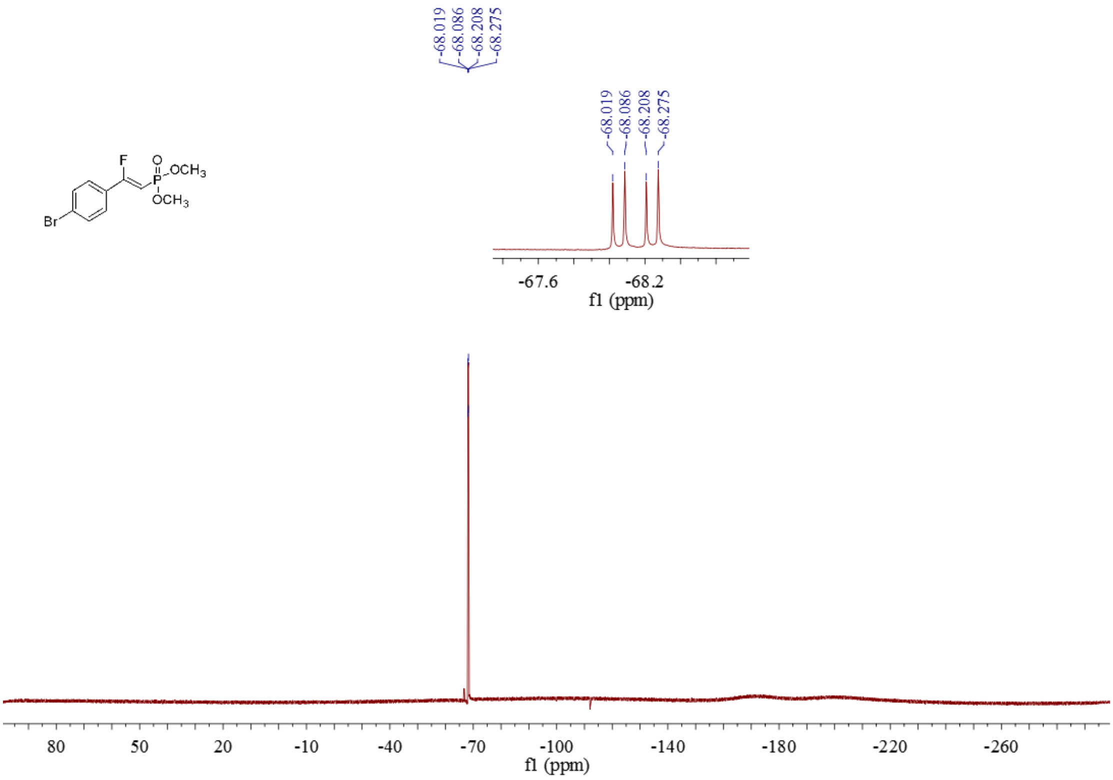

^{31}P NMR

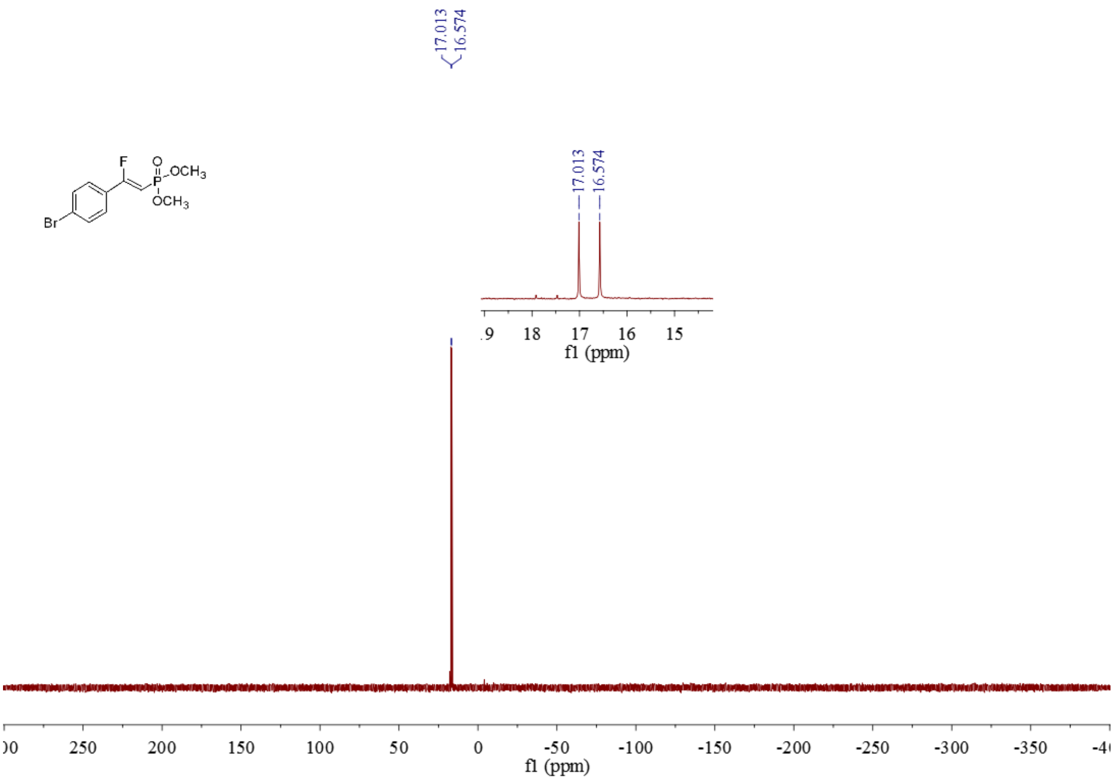

Supplement: File 1 — Experimental procedures, full characterization of products, and NMR spectra. [file Beilstein_J_Org_Chem-16-3086-s001.pdf]
